# Structure-based virtual screening of unbiased and RNA-focused libraries to identify new ligands for the HCV IRES model system†

**DOI:** 10.1039/d3md00696d

**Published:** 2024-03-18

**Authors:** Elisabeth Kallert, Laura Almena Rodriguez, Jan-Åke Husmann, Kathrin Blatt, Christian Kersten

**Affiliations:** a Institute of Pharmaceutical and Biomedical Sciences, Johannes Gutenberg-University Staudingerweg 5 55128 Mainz Germany kerstec@uni-mainz.de; b Institute for Quantitative and Computational Biosciences, Johannes Gutenberg-University BioZentrum I, Hanns-Dieter-Hüsch-Weg 15 55128 Mainz Germany

## Abstract

Targeting RNA including viral RNAs with small molecules is an emerging field. The hepatitis C virus internal ribosome entry site (HCV IRES) is a potential target for translation inhibitor development to raise drug resistance mutation preparedness. Using RNA-focused and unbiased molecule libraries, a structure-based virtual screening (VS) by molecular docking and pharmacophore analysis was performed against the HCV IRES subdomain IIa. VS hits were validated by a microscale thermophoresis (MST) binding assay and a Förster resonance energy transfer (FRET) assay elucidating ligand-induced conformational changes. Ten hit molecules were identified with potencies in the high to medium micromolar range proving the suitability of structure-based virtual screenings against RNA-targets. Hit compounds from a 2-guanidino-quinazoline series, like the strongest binder, compound 8b with an EC_50_ of 61 μM, show low molecular weight, moderate lipophilicity and reduced basicity compared to previously reported IRES ligands. Therefore, it can be considered as a potential starting point for further optimization by chemical derivatization.

## Abstract 2

Using structure-based virtual screening, FRET and MST assays, novel ligands of the hepatitis C virus internal ribosome entry site were identified. This proof-of-concept study demonstrated the feasibility of RNA–ligand docking for hit identification.

## Graphical abstract

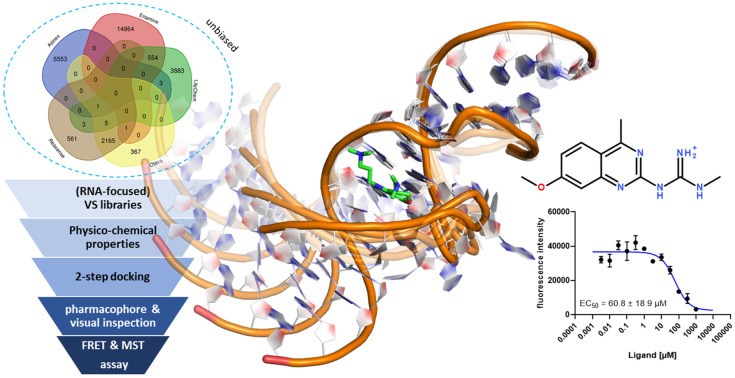

## Introduction

### Hepatitis C virus infections

Hepatitis C virus (HCV) infection can cause acute and chronic inflammation of the liver leading to hepatic fibrosis and cirrhosis in most of the chronic cases. The risk for development of hepatocellular carcinoma (HCC) is significantly increased by chronic HCV infection.^1^ HCC is the most frequent liver cancer, accounting for 70–80% of all cases, and is the fourth leading cause of cancer mortality.^2^ Approximately 290 000 people died from the corresponding disease, hepatitis C (and mostly due to cirrhosis and HCC), in 2019 following WHO estimations.^3^ Currently, around 58 million people are affected by chronic HCV infection worldwide.^3^ Central Asia and central Africa have one of the highest HCV prevalence rates followed by other regions in Asia, Africa, Eastern Europe, Australia, Latin America, and the Middle East identified in a worldwide study.^4^ HCV is a blood-borne virus, it bears high genetic diversity and produces diverse mutant clouds in infected people. Thus, development of an effective vaccine against hepatitis C was not successful so far and still is challenging. Current therapies target three significant non-structural (NS) protein components of the virus. The so-called class of direct acting antivirals (DAAs) belong either to protease or polymerase inhibitors and were subsequently approved for treating HCV infections starting in 2011. The combination of DAAs achieved up to 90% sustained virologic response, which is more than twice as good compared to previous treatments. However, the efficacy of DAAs is perturbed by the emerging resistance-associated amino acid substitution at drug target sites. Drug resistance can lead to treatment discontinuation or failure and is one of the major challenges in DAA treatment.^5^

### HCV IRES

Discovering new treatment options and addressing HCV on the RNA-level could circumvent this emerging resistance sustainably. HCV is an enveloped positive single-stranded RNA virus of the *Flaviviridae* family discovered in 1989.^2^ The translation of its RNA genome is regulated by an internal ribosome entry site (IRES), which is located in the 5′-untranslated region (UTR). IRES represents a virus' “trick” to accelerate the translation of its genome by avoiding most host cell initiation factors. It directly binds the 40S ribosomal subunit and requires only the eukaryotic initiation factors (eIF) 2 and elF3 to assemble into translation-competent ribosomes directly at the start codon.^6^ Thus, translation is initiated through a 5′-cap-independent mechanism. The 5′ UTR of the HCV can be divided into four distinct domains (I–IV), which are connected by flexible linkers. The stem loop in domain I is not essential for IRES activity and domain IV contains the start codon, which becomes exposed during initiation assembly.^7^ Domains II and III are crucial for IRES activity, in which domain III represents the core domain binding to eIF3 and ribosomal subunits.^6^ The release of eIF2 is mediated by domain II which also participates in formation of translation-competent ribosomes.^8^ Structures within domain II to IV can independently fold and the resulting three-dimensional organization of the IRES is crucial for its activity.^7^ The HCV IRES is highly structured, essential for virus survival and is the most sequence-conserved region of the HCV genome, thus being a promising drug target. The development of selective translation inhibitors offers new opportunities for less resistance prone treatment options.^9^

### Small molecule HCV translation inhibitors

**Fig. 1:**
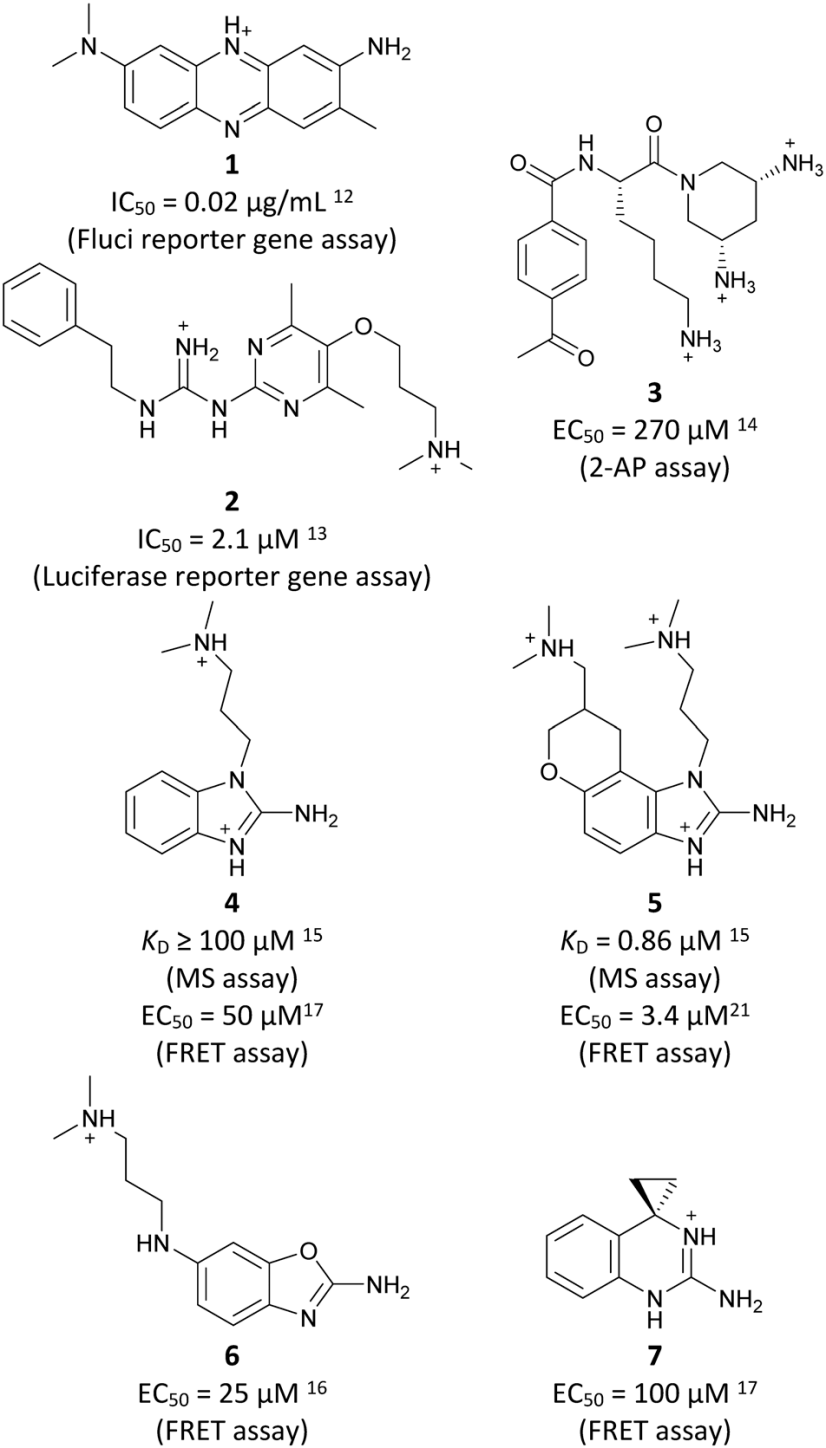
Molecular structures, binding and inhibition results of previously reported HCV IRES ligands. Molecules are depicted as the dominant protomer under physiological conditions. FRET: Förster resonance energy transfer, 2-AP: 2-aminopurine.

**Fig. 2:**
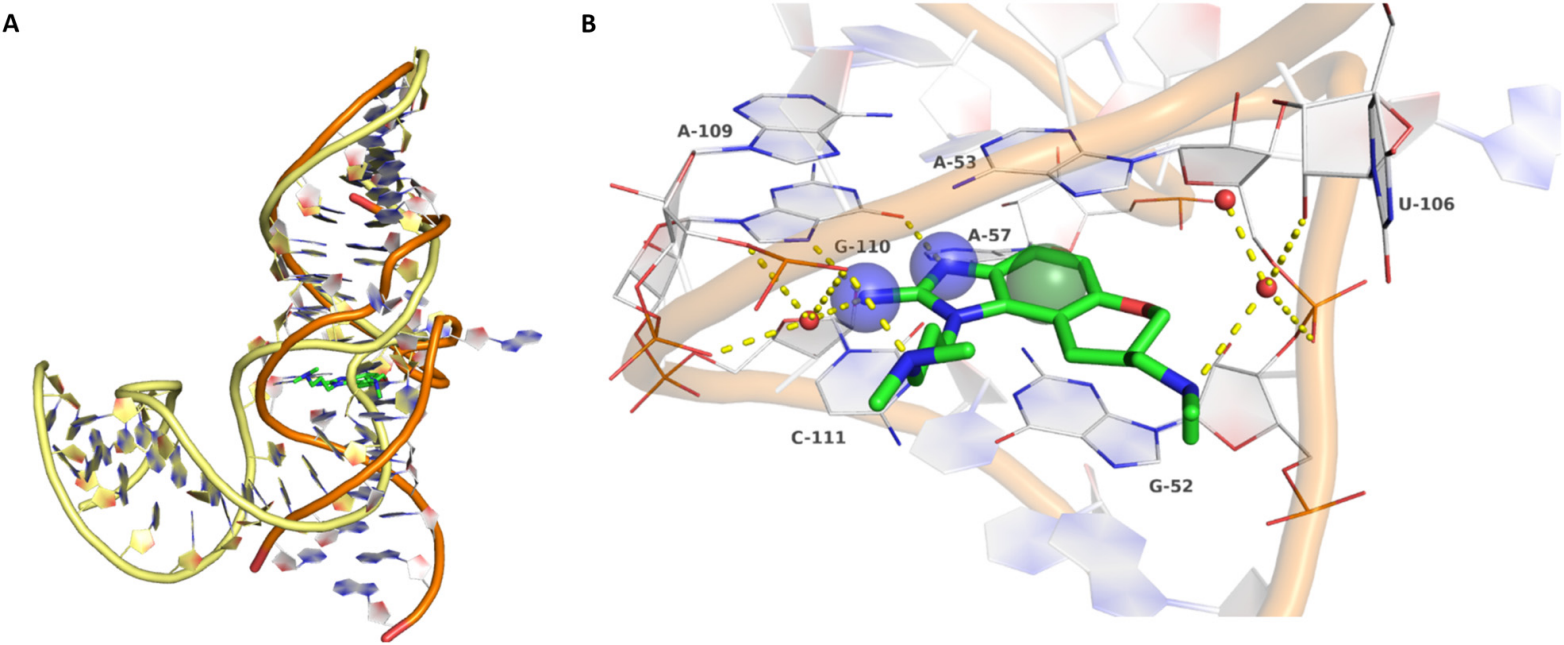
A) Superposition of ligand-free, kinked (yellow backbone, PDB-ID: 1P5M) and compound 5-bound, stretched (orange backbone, PDB-ID: 3TZR) HCV IRES subdomain IIa. B) Crystallographic binding mode of 5 (green carbon atoms) in complex with the HCV IRES subdomain IIa (white carbon atoms, PDB-ID: 3TZR). Direct and water-mediated (red spheres) polar interactions are depicted as yellow dashed lines. Key pharmacophore features are depicted as blue transparent spheres for hydrogen bond donors and a green sphere for an aromatic centre (pharmacophore feature radius 1.2 Å).

Inhibitors targeting different domains of the HCV IRES were identified since its discovery ranging from oligonucleotide-based inhibitors^10^ and peptidic inhibitors^11^ to small molecules.^7^ The field of small molecules covers different substance classes like phenazines^12^ (1), biarylguanidines^13^ (2), diamino-piperidines^14^ (DAP, 3) and 2-aminobenzimidazoles^15^ (4, 5) (Fig. 1). The initial 2-aminobenzimidazole hit (4) showed a *K*_D_-value of 100 μM (determined by a mass spectrometry (MS)-based assay) and subsequent structure–activity-relationship (SAR) analysis and derivatization achieved benzimidazole derivatives with sub-micromolar affinities. One optimized inhibitor is 5 with a *K*_D_-value of 0.86 μM which showed only minimal toxicity and antiviral activity in a cellular replicon assay.^15^ Thus, 5 is the most potent inhibitor reported so far. Further SAR exploration around this scaffold resulted in equally or less potent aminobenzoxazoles^16^ (6) and quinazolines^17^ (7). The classes of 2-aminobenzimidazole, quinazolines and DAP-conjugates were identified to bind to the IIa subdomain of the HCV IRES. DAP conjugates show effects on the IRES conformation upon binding different from 2-aminobenzimidazoles and quinazolines.^14^ X-ray crystallography studies^18,19^ of the native IRES subdomain IIa showed that two closely spaced divalent metal ions stabilize a right-angled bend at the intersection of two helices resulting in an L-shape of the RNA (Fig. 2A). This conformation is essential for IRES-driven translation to position the IIb hairpin at the 40S ribosomal subunit.^18–20^ Nuclear magnetic resonance (NMR) structural analysis of subdomain IIa in complex with 2-aminobenzimidazole 5 provided insights into an induced conformational switch. The inhibitor changes the approximately 90° bend to a more stretched shape with 23° widening of the interhelical angle. This provides a potential reason for translation inhibition (Fig. 2A).^8^ In addition, X-ray analysis and comparison of subdomain IIa with and without 5 bound revealed that three magnesium ions are present in both structures. They are regarded as intrinsic structural components which stabilize both, the free and the ligand-bound, states. The ions are not displaced upon ligand binding and are necessary for high-affinity interactions. Moreover, the ions undergo adaptive reorganization during the binding process.^21^ In contrast, DAP conjugates bind in competition with structural divalent metal ions and immobilize the L-shape of the RNA, which might be an alternative mechanism of inhibition.^14^ These conformational changes were monitored and confirmed *via* two different fluorescence assays.^14,22^

Binding of 2-aminobenzimidazole 5 to the highly conserved subdomain IIa induces a conformational change which forms a deep and well-defined binding pocket. An X-ray structure of subdomain IIa in complex with 5 at 2.2 Å resolution shows how the pocket encapsulates the ligand (Fig. 2B, PDB-ID 3TZR).^21^ The roof and the floor of the binding pocket are formed by stacking interactions between A53 and G52 with the benzimidazole scaffold. Further important RNA–ligand interactions comprise hydrogen bonding of the 2-amino-imidazole group to the guanine Hoogsteen edge of G110 as well as hydrogen bonding of the protonated dimethylamino-propyl side chain to the phosphate group of A109. If these key interactions with G110 and A109 are missing, no or weak binding to the HCV IRES subdomain IIa was observed.^17^ Thus, the 2-aminobenzimidazole and the dimethylaminopropyl functionality are crucial for this type of inhibitors. In addition, a basic substituent at the tetrahydropyran site is considered as beneficial and further substituents attached to the aromatic ring are not tolerated due to the tight fit into the pocket.^17^

The binding motif of 2-aminobenzimidazole translation inhibitors with the Hoogsteen edge of G110 and the intercalation between G52 and A53, may mimic interactions of arginine or guanosine with RNA nucleobases. A statistical evaluation of the amino acid–nucleotide interaction (AANT)^23^ database showed that the most likely interaction between an amino acid and any RNA nucleotide includes arginine or lysine residues. In the case of arginine, interactions with cytidine or guanosine were occurring more often compared to the other nucleotides.^23^ In addition, Hoogsteen pseudo pairs were found for G and arginine forming two hydrogen bonds. Arginine has three donor nitrogen atoms and is able to form four distinct pseudo pair geometries for both types of interactions. Interestingly, the Hoogsteen edge of A could be addressed by various amino acids, but the Hoogsteen edge of G was only recognized by arginine.^24^ In a more concrete example, the human immunodeficiency virus Tat protein can bind specifically to an RNA stem-loop structure (TAR) mediated by a single arginine residue. Arginine-rich sequences can also inhibit translation. Evidence can be found in a similar case, in which an arginine attenuator peptide can regulate its own translation by interacting with ribosomes.^25^ Arginine and G have been found to interact similarly with G:C base pairs.^26^ Hydrogen bond networks between G:C pairs with arginine or C:G–G triplets are similar to the observed Hoogsteen edge interactions between C58:G110 and 2-aminobenzimidazole 5 (Fig. S1A–C†).^21,27,28^ In a previous study, arginine, G and their derivatives were tested for HCV IRES subdomain IIa binding. While for arginine no binding could be observed, G and its derivatives showed some weak binding behavior (G, EC_50_ = 1.05 mM).^29^ Like 2-aminobenzimidazoles, G and its derivatives induced a conformational change by stretching the L-shape of the RNA and inhibited IRES-driven translation. Other nucleobases were inactive. It was hypothesized that a guanosine of the same RNA strand located in domain IV autoregulates HCV IRES activity. At the beginning, trigger guanosine is sequestered in a hairpin loop in domain IV to allow ribosomal assembly. Positioning of the start codon at the decoding site induces movement in domain IV which unmask the trigger guanosine. Guanosine induces a conformational change by stretching subdomain IIa, which facilitates IRES release from the ribosome.^29^

In this work, a virtual screening was performed to identify new scaffolds with reduced basicity (compared to 5 and derivatives), binding to the HCV IRES subdomain IIa. This will not only obtain alternative molecules as starting points for optimization in terms of HCV DAA resistance-mutation preparedness, but also serve as a proof-of concept model system for structure-based RNA–ligand design for RNA targets which do not have a native small molecule ligand like riboswitches.^30–34^ The crystal structure of the HCV IRES subdomain IIa in complex with 5 and previous SAR studies were used as a starting point for the structure-based virtual screening.

## Results

### Structure-based virtual screening

Despite the increasing interest^35–38^ in RNA-targeting small molecules, structure-based virtual screenings for RNA-targets are still in its infancy.^39–41^ One challenge is the high flexibility of RNA, which is simplified in this study by using the RNA–ligand complex structure for docking, assuming similar conformational changes upon ligand binding as observed for compound 5. Likewise, while unbiased screening libraries against RNAs showed hit-rates comparable to screenings against proteins,^33,34,42,43^ several studies identified privileged scaffolds and physicochemical properties for RNA-targeted chemical spaces.^44–49^ To account for this recent development, additionally to our unbiased in-house virtual library, also RNA-focused libraries of different chemical suppliers were separately considered in the virtual screening against the HCV IRES subdomain IIa. Except for Otava and Reaxense, the RNA-focused libraries showed only little overlap (Fig. S2A†). Physicochemical properties were comparable to the unbiased VS library and showed only slight differences in moderately increased numbers of aromatic and basic (N) atoms in line with previous observations from R-bind (Table S1†).^45,50^ Higher formal charges were found for the Asinex and Enamine libraries, while reduced log *P*-values or higher numbers of hydrogen bond donors as described previously^50^ for RNA–ligands, were not found. After further filtering for target-specific physicochemical properties (Table S2†), molecules were subjected to a two-step docking process (Fig. S2B†). The molecular docking workflow was validated by re-docking and binder *vs.* non-binder and decoy discrimination (Fig. S3A–I†). During prospective docking, not only docking scores, but also generated binding poses were evaluated. Especially matching of the crystallographic reference ligand's key interactions (Fig. 2B) was heavily weighted in this process. As indicated in previous SAR studies, aromatic stacking between G52 and A53, as well as two hydrogen bond donor functionalities to interact with the Hoogsteen edge of G110 are common themes in many binders. Exemplarily, the exchange of the 2-aminobenzimidazole (5) moiety to 2-aminobenzoxazole (6) resulted in a loss of potency^16^ (Fig. 1) as the hydrogen bond donor functionality of the protonated 2-aminobenzimidazole is lacking in the less basic and non-protonated 2-aminobenzoxazole. 18 molecules (8–25, Table S3†) with high docking scores and best mimicking of the interaction profile of 5 were selected for *in vitro* testing. Notably, even though the fraction of molecules passing each triage step was higher for RNA-focused libraries compared to the unbiased library, in the end only molecules from the unbiased library were selected for testing. Some derivatives of obtained compounds were also present in the Enamine RNA-focused library, as well as molecules from this supplier in the unbiased library that were not part of the RNA-focused library. For the given example case of the HCV IRES subdomain IIa, this finding indicates that RNA-focusing can increase virtual hit rates, but current commercial libraries are too small to result in overall high hit numbers (Fig. S2B†).

### Binding studies and SAR analysis

None of the molecules selected for *in vitro* testing were flagged as pan-assay interference compounds (PAINs).^51,52^ However, so far there is no unified “RNA-PAINs” or promiscuous binder concept available. Therefore, HCV IRES subdomain IIa interactions were assessed with two independent assays. These assays were validated with reported HCV IRES ligands 4 and 5. In a FRET-assay, the conformational change of subdomain IIa upon ligand binding (Fig. 2A) was monitored as described previously (Fig. S4, S6 and S7†).^22,53,54^ In an MST-assay, binding affinity was measured (Fig. S5 and S8†). To exclude interference with the Cy3- and Cy5-labels, excitation and emission spectra of all compounds were recorded showing no interference. Identity and purity >95% of most compounds was verified by LC-MS and NMR analytics (Table S3, ESI:† 4. Analytical data of virtual screening hits). However, from the molecules identified as hits in the assays, 9, 10 and 12 showed purity below 95%. After an initial screening at four different ligand concentrations (Fig. S4 and S5†), dose–response curves were recorded for compounds that showed binding in the initial screening (Table 1, Fig. S6 and S8†). In some cases, EC_50_- and *K*_D_-values could not unambiguously be determined due to weak binding affinity or limited solubility over 1 mM concentrations (indicated as a value lower limit *via* ≥).

**Table 1:** FRET and MST results for HCV IRES reference ligands 4 and 5, virtual screening hits 8–13, 8-derivatives 8a–8c and amiloride (26). Pre-screening, MST time-traces, dose–response curves, and selectivity assays are summarized in Fig. S4–S10.† Molecules are depicted in their dominant protomeric/tautomeric state under physiological conditions. For 12, 1*H*-pyrimidine protomers/tautomers are also reasonable. N.b. no binding observed. Me: methyl, Et: ethyl

Compound	FRET EC_50_ [μM]	MST *K*_D_^a^ [μM]	Selectivity^b^
4	78 ± 35	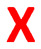	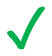 c
5	4.9 ± 2.6	5.2 ± 0.8	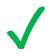 c
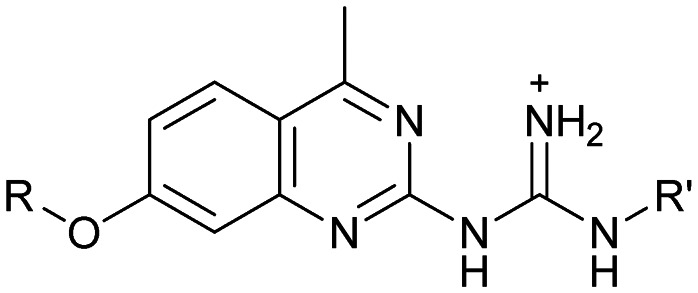			
8 (R: Et, R′: Me)	413 ± 97	≥440	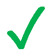
8a (R: Et, R′: H)	88 ± 12	189 ± 20	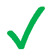
8b (R: Me, R′: Me)	61 ± 19	88 ± 2	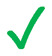
8c (R: Me, R′: H)	120 ± 24	134 ± 43	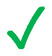
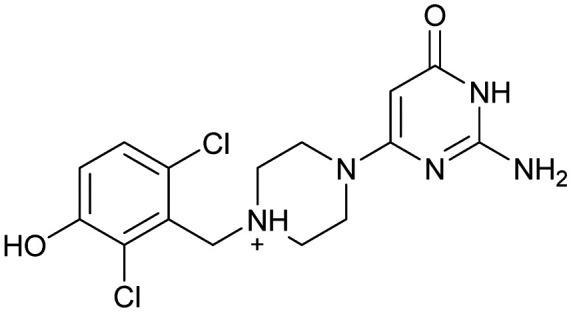	217 ± 41	n.b.	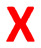 c (*K*_D_ = 7.8 ± 3.3 μM preQ_1_ riboswitch)
9^e^			
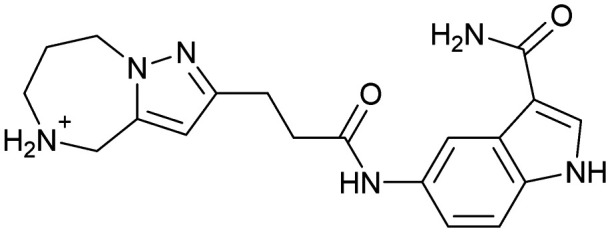	≥500^d^	n.b.	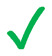
10^e^			
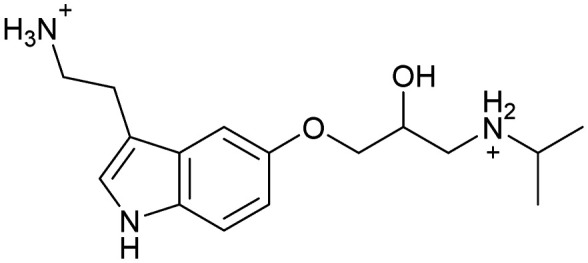	≥500	≥100	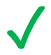
11			
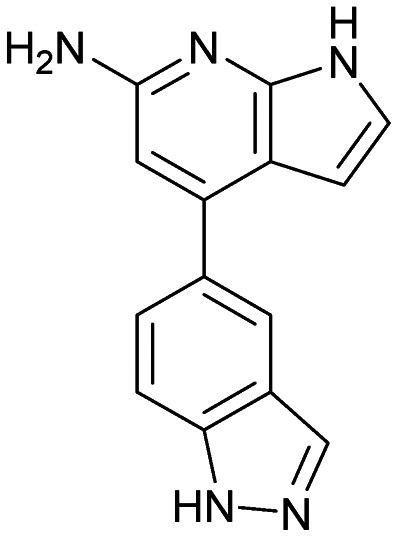	≥300	≥50	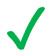
12^e^			
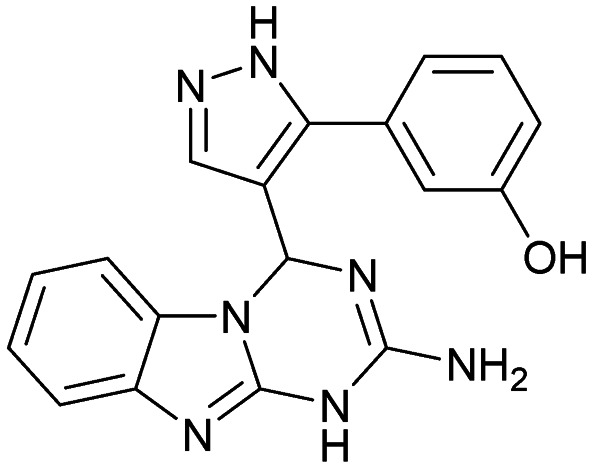	≥1000	≥100	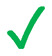
13			
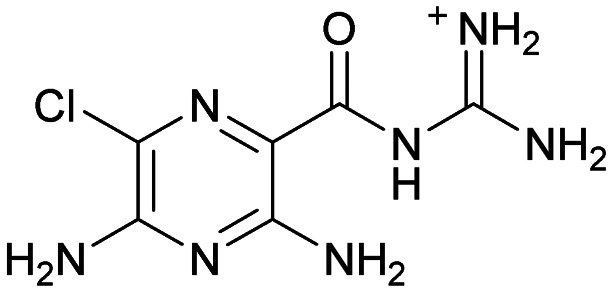	≥500	≥100	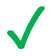
**26 (amiloride)**			

a MST binders are defined as molecules that clearly show a concentration-dependent MST-shift, but do not reach saturation due to solubility limitations and therefore no upper plateau in the dose–response curve. This is indicated by the presentation of *K*_D_-values as a lower limit (≥).

b Molecules are defined to be selective if no MST-shift is observed for the unrelated preQ_1_-riboswitch (Fig. S9†) and the FRET signal in presence of competing total RNA is vastly unaffected (Fig. S10†).

c 5 and 9 showed MST-shifts in the preQ_1_-riboswitch MST assays at high concentrations, but not for SAM-VI-riboswitch binding. EC_50_-values of 4 and 9 were reduced in presence of total RNA and did not reach saturation.

d Based on decreased Cy5-emission. Cy3-emission did not increase for cpd. 10.

e Purity of 9, 10 and 12 was below 95% determined by LC-MS and NMR analytics.

**Fig. 3:**
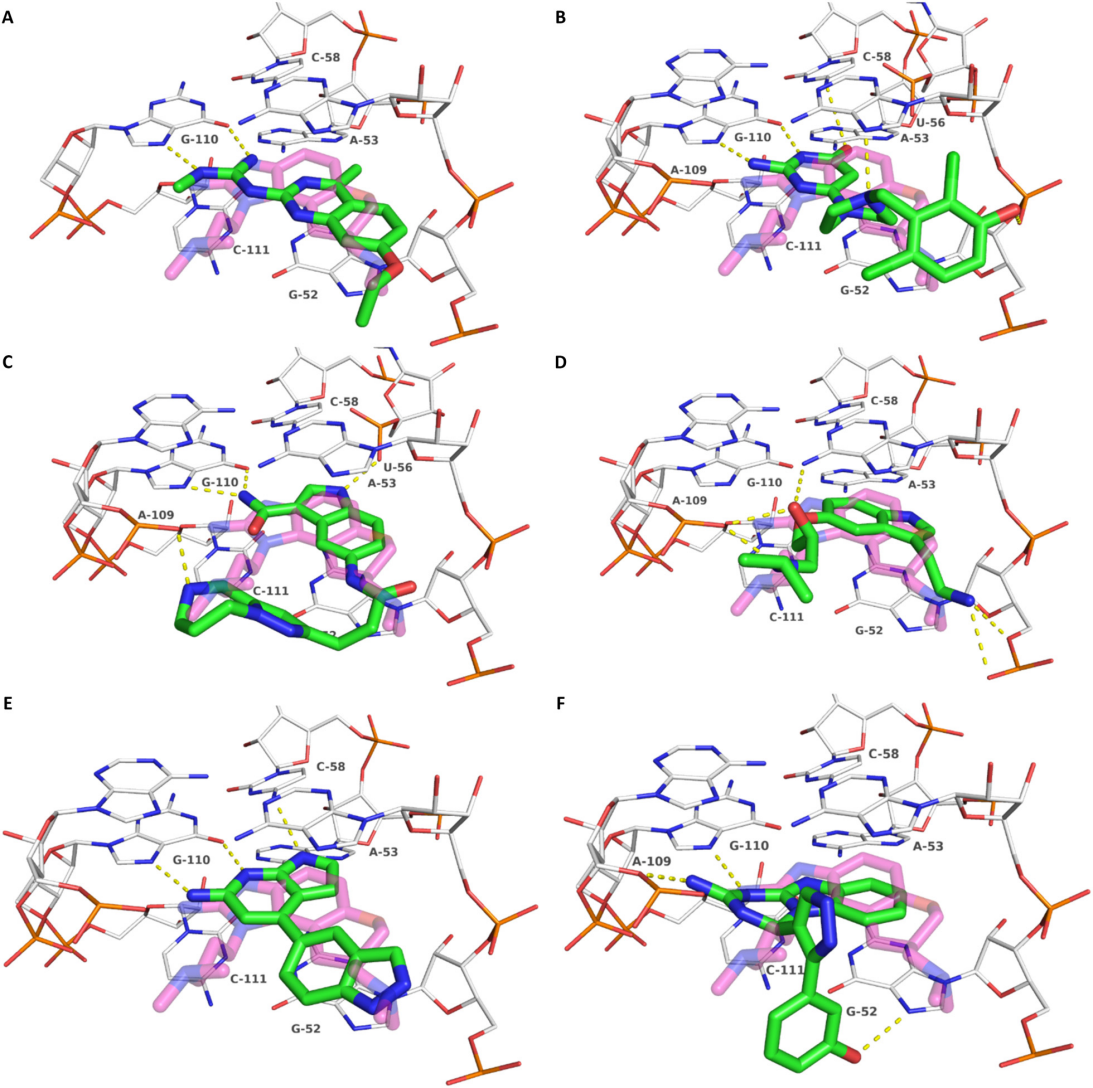
Docking-predicted binding modes of 8 (A), 9 (B), 10 (C) and 11 (D), 12 (E) and 13 (F). Ligands are depicted with green carbon atoms, HCV IRES with white carbon atoms (PDB-ID: 3TZR). The crystallographic reference ligand (5) is shown with transparent, magenta-colored carbon atoms for orientation. Polar interactions are depicted as yellow dashed lines.

To exclude promiscuous binding behavior, two selectivity assays were performed. First, interactions with an unrelated and assayable RNA, the *Thermoanaerobacter tengcongensis* (*Tte*) preQ_1_-riboswitch aptamer domain, was evaluated for all compounds that showed binding to the HCV IRES *via* MST (Fig. S9A–O†). For compounds 5 and 9, which showed binding to the preQ_1_-riboswitch at very high concentrations, an additional selectivity MST assay with the *B. angulatum* SAM VI-riboswitch aptamer domain was conducted (Fig. S9P and R–T†). There, no MST shift and hence, no general RNA-binding promiscuity for these compounds was found. Second, the FRET assay was repeated for hit compounds in presence of a 5-fold (m/m) excess of competing total RNA (Fig. S10†). Six VS hits (8–13, Table 1, Fig. 3) were identified as specific binders of the HCV IRES subdomain IIa.

Compound 8 with EC_50_ = 413 μM and *K*_D_ ≥ 440 μM contains a 2-guanidino-pyrimidine moiety, a feature already reported in HCV IRES ligand 2 (Fig. 1) which is reminiscent of the hypothesized G110–arginine interaction (Fig. 3A and S1B†).^7^ Consequently, further derivatives of compound 8 were obtained. Derivatives 8a–8c showed binding to the HCV IRES subdomain IIa in the MST assay, induction of the conformational change in the FRET assay, and selectivity both in the total RNA competition assay and preQ_1_ binding assay (Fig. S6–S10†). From these molecules, 8b showed the highest potency with EC_50_ = 61 μM and *K*_D_ = 88 μM. A small, fragment-like size (18 heavy atoms, ligand efficiency LE 0.31 kcal mol^−1^), moderate lipophilicity (log *P* 1.1,^55^ lipophilic ligand efficiency LLE 2.9)^56^ and reduced basicity (one basic center compared to three in compound 5 and 2-aminobenzimidazole derivatives) makes this molecule a potential starting point for further optimization. Guanidinium-containing molecules like in several amiloride derivatives, were repeatedly reported as RNA–ligands.^57–63^ Adding amiloride (26) to the testing panel showed some weak activity with EC_50_ ≥ 500 μM and *K*_D_ ≥ 100 μM (Fig. S6L and S8L†) as well as selectivity over the preQ_1_-riboswitch (Fig. S9O†). Camostat (27, Fig. S11A†), a benzylguanidine, revealed no binding to subdomain IIa (Fig. S4Y and S5Y†). This indicates that, despite their reoccurrence as RNA–ligands, guanidine moieties attached to aromatic systems might be considered as privileged scaffolds rather than promiscuous RNA-binders. Except compounds 8, 8a–8c, hits from the virtual screening show only low affinity in the FRET and MST assays. While still being selective over unrelated RNAs (Fig. S9 and S10†), only limited insights in interaction patterns with the HCV IRES subdomain IIa are possible from these compounds 9–13. Compound 9 with EC_50_ = 217 μM, but no significant MST shift, contains a Watson–Crick-like edge as found in G within its 2-amino-1*H*-pyrimidin-6-one substructure. Based on the predicted binding mode (Fig. 3B), this moiety mimics interactions as found for G–G:C interactions (Fig. S1C†). Further molecules containing this substructure (14, 15, Table S3†) showed no binding. As the essential structural difference between 9 and 14/15, the basic moiety of 9 in the tertiary aliphatic amine of the piperazine ring interacting with A53 is assumed. 14 and 15 contain no basic nitrogen, but an amide. Notably, differently from the other hits, 9 also binds to the preQ_1_-riboswitch with a *K*_D_-value of 7.8 μM (Fig. S9G and P†). This lack of selectivity can be explained by the preference of that riboswitch to G-like compounds.^31,64^ In a recent study,^31^ compound 28 (Fig. S11B†) with high similarity to 9 was identified as a preQ_1_-riboswitch ligand. Cross-testing of 28 against the HCV IRES subdomain IIa, however, showed no binding (Fig. S5Z†). Additional selectivity tests of 9 and 28 against the unrelated SAM-VI-riboswitch also showed no binding (Fig. S9S and T†). Thus, 9 seems to be not generally promiscuous, but eventually preferred in RNA recognizing G-like Watson–Crick edges. This is also reflected in the FRET competition assay in presence of total RNA, where the EC_50_-value of 9 is reduced, but the reached plateau for Cy5-fluorescence shows higher values, indicative for off-target RNA-binding. Compounds 10 and 11 share an indole core with two substituents at the 3- and 5-position connecting to one (10) or two (11) basic centres resulting in EC_50_-values of ≥ 500 μM, respectively. Molecular docking poses show different indole orientations (Fig. 3C and D) and cannot satisfy all pharmacophore features. Predicted binding modes still resemble an overall similar shape to 2-aminobenzimidazole 5. While 10's and 11's basic centers are close to the phosphate backbone of A109, the docking pose of 11 additionally shows an ionic interaction with the backbone of G52. Only 11, but not 10 also showed binding in the MST-assay with a *K*_D_-value of ≥ 100 μM. Compound 12 with an EC_50_ ≥ 300 μM and *K*_D_-value ≥ 50 μM, contains a slightly basic 4-substitued pyrrolo[2,3-*b*]pyridine-6-imine moiety well meeting the desired pharmacophore features (Fig. 3E). 3,4-Dihydrobenzo[4,5]imidazo[1,2-*a*][1,3,5]triazin-2-amines (13, 16–22, Table S3†), as well as benzamidines (23 and 24) are structurally related and reoccurring scaffolds among the best scoring molecules from the virtual screening. However, these showed no to very weak HCV IRES subdomain IIa binding. Only compound 13 from the 3,4-dihydrobenzo[4,5]imidazo[1,2-*a*][1,3,5]triazin-2-amine series (Fig. 3F, S6K and S8H†) showed very weak binding in both FRET (EC_50_ ≥ 1 mM) and MST (*K*_D_ ≥ 100 μM) assays. 18, 21 and 22 showed only slight, concentration-dependent MST-shifts (Fig. S5P, S and T†), but no large changes in FRET-signals (Fig. S4†) were observed. Overall, limited ligand potency hinders strong conclusions except for compounds 8 and 8a–8c. Additionally, identified hits do not represent the molecules with the highest docking scores (Table S3†), but those well representing interactions observed in the HCV IRES IIa – compound 5 crystal structure (Fig. 2B) which is indicative for the requirement of some (human introduced) ligand-bias during hit selection for RNA-targeting molecular docking screens.

## Discussion and conclusions

A virtual screening of several RNA-focused and an unbiased library of drug-like small molecules against the HCV IRES subdomain IIa was performed. VS hit triage revealed that a larger fraction of molecules from the RNA-focused libraries is passing the respective filter steps compared to the unbiased library (Fig. S2B†). However, the overall numbers of commercially available compounds for focused libraries were too small for final hit selection. Thus, all molecules selected for testing originate from the unbiased library. We hypothesize that focusing of larger VS libraries or even make-on-demand chemical spaces^65–68^ has the potential to result in higher hit rates and numbers. Eventually, the general concept of RNA-focusing might be insufficient. A more refined approach for the specific target or target class of interest should be used like for proteins rather than thinking of “RNA-focused”. Instead of “protein-focused” libraries, kinase-, GPCR- or serine protease-focused libraries are used there. While this study is only a one-target test case, further studies are needed to refine our understanding of RNA-targeting (or riboswitch-, three-way junction-, internal loop-, triple helix-targeting, …) chemical space. Likewise, the knowledge of key-pharmacophore features of previously reported HCV IRES subdomain IIa ligands (Fig. 2B) was exploited during pose inspection to select VS hits for testing. Using two independent assays, an MST-binding assay and a FRET-based assay to monitor conformational changes of the target RNA, several VS hits could be confirmed as binders of the HCV IRES subdomain IIa with low to moderate potency (Table 1, Fig. 3). From six FRET- and seven MST-hits, four compounds (8, 11–13) were identified in both assays. For the 2-guanidino-quinazoline compound 8, further derivatives (8a–8c) were obtained for testing, representing the strongest binders in this study. The most potent ligand, compound 8b (EC_50_ = 61 μM, *K*_D_ = 88 μM), shows structural similarities with the previously reported ligands 2 and 7 (Fig. 1), reminiscent of the interaction profile of arginine with G Hoogsteen edges (Fig. 3A and S1B†). Testing of amiloride (26) due to structural analogy, a privileged RNA-binding scaffold,^57–63^ showed some weak binding as well. From the less potent single-assay hits, on the one hand, 9 and 10 showed signals in the FRET assay, but not in MST. Notably, MST-shifts are highly ligand-dependent and even though being a very sensitive^69^ method, MST can therefore miss hits (false negatives). Hence, 9 might still be considered a weak IRES binder that induces a conformational change, especially when considering the prevalence of G-like moieties for G Hoogsteen edge binding in G–G:C motifs (Fig. S1C†).^29^ This is also reflected in the observed preQ_1_-riboswitch binding and altered FRET-behaviour in presence of competing RNA (Fig. S10G†). Compound 10 only showed a decrease in the Cy5-signal, but no increase of Cy3-emission (Fig. S7H†) which might be an indicator for unspecific binding behavior. On the other hand, compounds 18, 21 and 22 induced a slight MST-shift, but no changes in the FRET-assay, hinting for no to very weak or even unspecific binding that does not effectively induce conformational changes of the IRES subdomain IIa. A selectivity counter-screen with the structurally unrelated preQ_1_-riboswitch and a competition assay in presence of a five-fold (m/m) excess of total RNA, showed selectivity of all VS hits except 9. However, G-derivatives^31,64^ are known binders of the preQ_1_-riboswitch recognizing the 1*H*-pyrimidin-6-one moiety of compound 9. This site might be recognized by other RNAs present in the used total RNA as well. Additional testing with the SAM-VI-riboswitch showed no general RNA-promiscuity of compound 9. Finally, from the virtual screening, most hits showed only very low potency (9–13). Only compound 8 and derivatives 8a–8c reached saturation in dose–response curves for both FRET and MST assays while being selective in the competition assay and the preQ_1_-riboswitch counter screen. Therefore, only these compounds can unambiguously be described as specific HCV IRES subdomain IIa ligands. Identified 2-guanidino-quinazoline hits like compound 8b (LE: 0.3 kcal mol^−1^, LLE: 2.9) from this study can be considered as a potential starting point for derivatization and optimization. None of the hit compounds reaches the potency of previously reported 2-aminobenzimidazole ligands.^15,17,21,22,53,70–72^ However, the reduced basicity of hit compounds compared to three basic nitrogen atoms in compound 5, might result in beneficial physico-chemical properties. Additionally, this study can be considered as a proof-of-concept for the so far very rare field of structure-based virtual screenings against RNA-targets. Using protein-based tools and additional care during hit selection by pharmacophore elucidation, small molecular weight binders of the HCV IRES subdomain IIa were identified. Consequently, findings from this study can help to improve the field of RNA-targeting molecular docking screens hinting towards the implementation of bias from known ligands.

## Material and methods

### Virtual screening

For molecular docking, the HCV IRES subdomain IIa in complex with the 2-aminobenzimidazole ligand 5 was derived from the protein data bank (PDB,^73^ PDB-ID: 3TZR^21^). FRED^74,75^ (FRED 4.0.0.0 OpenEye Scientific Software, Santa Fe, NM. http://www.eyesopen.com), LeadIT (LeadIT-2.3.2, BioSolveIT GmbH, St. Augustin, Germany, 2017, https://www.biosolveit.de/) and FlexX^76^ (FlexX Version 4.5.1, BioSolveIT GmbH, St. Augustin, Germany, 2020, https://www.biosolveit.de/FlexX) were demonstrated to be suitable for RNA–ligand docking without further modifications and treating the target RNA as rigid previously.^31^ Docking setups were validated by re-docking and binder-decoy discrimination using 40 reported binders and 15 non-binders^15–17^ as well as 1200 decoys generated using the database of useful decoys enhanced (DUD-E).^77^ For LeadIT and FlexX docking, molecules were protonated with MOE (Molecular Operating Environment (MOE); 2020.09; Chemical Computing Group ULC: 1010 Sherbrooke St. West, Suite #910, Montreal, QC, Canada, H3A 2R7, 2020. https://www.chemcomp.com/index.htm) and energetically minimized using OMEGA^78^ (OMEGA 4.1.0.0: OpenEye Scientific Software, Santa Fe, NM, USA. http://www.eyesopen.com, 2019). For FRED docking, conformers were generated with OMEGA using the “pose” keyword. For FRED and FlexX docking, water molecules and magnesium ions were removed from the binding site. Two magnesium ions (MG68 and MG70) close to the reference ligand were only indirectly considered by this removal to avoid overestimation of ion-complexing ligand moieties. However, ligands expanding deeply into the MG68 sub-pocket were also removed from hit-selection as binding strength/impact of displacement of Mg^2+^ to RNA is difficult to estimate. For LeadIT docking, water molecules that form at least three interactions with the ligand or target, namely water molecules HOH-6, HOH-71 and HOH-92 from PDB-ID 3TZR (Fig. 2B), were kept, but allowed to be displaceable or re-oriented during docking to allow poses with both direct or indirect, water-mediated interactions. Re-docking root-mean-square-deviation (RMSD) values of 1.22 Å, 1.23 Å and 2.08 Å (Fig. S3A–C†), receiver operating characteristics (ROC) area under the curve (AUC) of 0.88, 0.97 and 0.70 (Fig. S3D–F†) and adjusted ROC–log AUC_0.1–100%_^79–81^ of 0.44, 0.35 and 0.09 (Fig. S3G–I†) for FRED, LeadIT and FlexX, respectively were obtained. Due to the weak performance of FlexX, likely caused by automatic deprotonation of 2-aminobenzimidazole-moieties during docking (Fig. S3C, F and I†), the prospective virtual screening was continued with FRED as a first step, followed by LeadIT, where pre-defined protonation states from MOE were used instead of using the Protoss^82^ routines of LeadIT/FlexX. RNA-focused libraries were obtained from the respective supplier homepages of Asinex (combined focused libraries: “RNA-targeting”, “dinucleoside mimetics”, “fragments for RNA”, “macrocycles for RNA”, https://www.asinex.com/screening-libraries, accessed: 2021/08/13), Enamine (https://enamine.net/compound-libraries/targeted-libraries, accessed: 2021/08/13), Life Chemicals (https://lifechemicals.com/screening-libraries/pre-plated-focused-libraries, accessed: 2021/08/13), Otava (https://www.otavachemicals.com/products/targeted-libraries-and-focused-libraries/other-focused-libraries, accessed: 2021/08/13) and Reaxense (https://www.reaxense.com/products/focused-libraries/rna-targeted-focused-library, accessed: 2021/08/13). Molecules were “washed” to remove ions and protonated with MOE. PAINs^51,52^ removal and physicochemical property filters (Table S2†) were applied using FILTER (OMEGA 4.1.0.0: OpenEye Scientific Software, Santa Fe, NM, USA. http://www.eyesopen.com, 2019). Molecules were docked with FRED as described for the model validation. Molecules with a ChemGauss score of ≤−13.0 kcal mol^−1^ or matching at least two pharmacophore features were processed and subsequently docked with LeadIT prior visual pose inspection^83^ and selection for testing.

### Chemical analytics

Reference compound 4 was purchased from Chembridge/Hit2Lead. Compound 5 was synthesized according to published procedures^70,72^ (see extended Material and methods section of the ESI†). Virtual screening hits were purchased from Chembridge/Hit2Lead (9–12, 14–16, 25), Enamine Ltd. (17, 18, 20–23) and Vitas-M (8, 13, 19). All purchased compounds were provided with confirmed identity and purity by LC-MS or ^1^H-NMR analytics. Identity and purity of all compounds were re-determined in-house by HPLC-ESI/MS (Table S3†) using a 1100 series HPLC system from Agilent with a Poroshell 120 EC-C18 150 × 2.10 mm, 4 μm column if not stated differently and ^1^H- and ^13^C-NMR analytics (ESI,† 4. Analytical data of virtual screening hits) on a Bruker Avance Neo 400 MHz NMR spectrometer. The LC-MS measurements were conducted with a gradient of acetonitrile and water (+0.1% formic acid) ranging from 10% to 90% acetonitrile over 10 min with a flow rate of 0.7 mL min^−1^. Signals were detected at 254 nm with quantification by area under curve (AUC). The molecular mass was detected using an Agilent 1100 series mass-selective detector trap with positive mode electron spray ionization. The LC-MS chromatograms and their corresponding mass spectra were analyzed using MestReNova (version 12.0.4, Mestrelab Research S.L. Feliciano Barrera 9B, Bajo, 15706 Santiago de Compostela, Spain. http://www.mestrelab.com). To exclude interference with the Cy3- and Cy5-labels, excitation and emission spectra of all compounds were recorded. None of the molecules showed excitation or emission in the range of Cy3 (excitation: 480 nm, emission: 535 nm) and Cy5 (excitation: 633 nm, emission: 670 nm).

### FRET assay

For the FRET assay, a two stranded HCV IRES subdomain IIa carrying a 5′-Cy3-label on one and 5′-Cy5-label on the other strand (Cy3-UCGGAGGAACUACUGUCUUCACGCC, Cy5-UGCGUGUCGUGCAGCCUCCGG, Biomers.net GmbH) was used as described previously.^22,53^ All samples containing labelled RNA were treated under light protection. FRET experiments were performed on a Tecan Spark 10 M plate reader (excitation/emission: 480/670 nm) using black half area 96 well microplates (Greiner Bio-One), based on a protocol by Zhou *et al.*^53^ The 5′-Cy3- and 5′-Cy5-labelled single strands were annealed by heating to 75 °C in FRET buffer (10 mM HEPES pH 7.0, 2 mM MgCl_2_) and cooled down to room temperature for at least 20 min. The 5′-Cy3/Cy5-labelled RNA was diluted to 100 nM in FRET buffer and supplemented with compounds 8–28 in appropriate concentrations. Compounds 4 and 5 were used as positive controls. For the initial screening, compounds were added in final concentrations of 1000, 100, 10 and 0.001 μM (Fig. S4†). If a concentration dependent reduction of the FRET signal (Cy5-emission) was observed, EC_50_-values were determined using ligand concentrations from 1 mM to 100 nM in 3.16-fold (semi-logarithmic) dilution series. For those compounds, the Cy3 emission was measured at 535 nm to confirm a conformational change dependent reduction in the Cy5 emission signal *via* Cy3 emission increase (Fig. S7†). For each experiment, one DMSO negative control was carried out. The final DMSO concentration was always 2%. Excitation and emission spectra were recorded for the Cy3/Cy5-labelled RNA (100 nM) in FRET buffer to confirm excitation (Cy3) and emission (Cy5) wavelength as well as for each compound (1 μM) in FRET buffer to exclude any interference with the cyanine dyes. All measurements were performed at least as triplicates for screening and EC_50_-determination.

For the HCV IRES-binding compounds (4, 5, 8–13, 8a–8c), a selectivity FRET assay in presence of total RNA from yeast (Roche) was performed. The ratio of the FRET HCV IRES subdomain IIa construct and the total RNA was set to 1 : 5 m/m. The preparation of the labelled RNA and the compounds was the same as in the binding assay. The yeast RNA was diluted to 7.88 mg L^−1^ and supplemented with 100 nM HCV IRES and appropriate concentrations of the compounds (3.16-fold dilution series) in FRET buffer (Fig. S10†). For each compound, triplicate measurements were performed. For each experiment, one DMSO negative control was carried out. The final DMSO concentration was always 2%.

FRET experiments were analyzed using GraphPad Prism (version 8.0.1., GraphPad Software, Boston, Massachusetts USA, https://www.graphpad.com).

### MST assay

For the MST assay, the same sequence of the two stranded HCV IRES subdomain IIa as for the FRET assay was used. One strand was 5′-Cy5-labelled and the other strand was unmodified (UCGGAGGAACUACUGUCUUCACGCC, Cy5-UGCGUGUCGUGCAGCCUCCGG, Biomers.net GmbH). All samples containing labelled RNA were treated under light protection. MST experiments were performed on a Monolith NT.115 from NanoTemper Technologies using standard uncoated capillaries. The strands were annealed by heating to 75 °C in MST buffer (10 mM HEPES pH 7.0, 2 mM MgCl_2_) and cooled down to room temperature for at least 20 min. The 5′-Cy5-labelled HCV IRES domain IIa RNA was diluted to 30 nM in MST buffer and supplemented with compounds 8–25 in appropriate concentrations. Compounds 4 and 5 were used as positive controls. For the initial screening, compounds were added to final concentrations of 1000 (or 316 if limited by solubility), 100, 10 and 0.001 μM (Fig. S5†). If a concentration-dependent shift of thermophoresis was observed, *K*_D_-values were determined using concentrations ranging from 1 mM (if permitted by solubility, otherwise starting from 316 or 100 μM) to 0.01 μM in a 3.16-fold (semi-logarithmic) dilution series. For each experiment, one DMSO negative control was carried out. Final DMSO concentration was always 2%.

Off-target counter screens by MST were performed as described previously.^31,84^ Briefly, the 5′-Cy5-labelled *Tte* preQ_1_-Riboswitch aptamer domain (Biomers.net GmbH) was heated to 75 °C for 5 min, cooled down to room temperature for at least 20 min and then diluted to 20 nM in MST buffer (50 mM Tris-HCl pH 7.5, 100 mM KCl, 25 mM MgCl_2_) and supplemented with compounds 4, 5, 8–13, 18, 21, 22 and 26 to final concentrations of 1000, 100, 10 and 0.001 μM. PreQ_1_ was used as a positive control. The 5′-Cy5-labelled *B. angulatum* SAM VI-Riboswitch aptamer domain (Eurofins Genomics GmbH) was heated to 75 °C for 5 min, cooled down to room temperature for at least 20 min and then diluted to 20 nM in MST buffer (50 mM Tris-HCl pH 7.5, 100 mM KCl, 25 mM MgCl_2_) and supplemented with compounds 5, 9 and 28 to final concentrations of 1000, 100, 10 and 0.001 μM. SAM was used as a positive control. For each experiment, one DMSO negative control was carried out. The final DMSO concentration was 2%. All measurements were performed at least as duplicates for pre-screening and triplicates for *K*_D_-determination. MST measurements were analyzed from signals after 18 s laser on-time using the MO.Affinity Analysis software (version 2.3) and exported for statistical analysis and plotting in GraphPad Prism (version 8.0.1., GraphPad Software, Boston, Massachusetts USA, https://www.graphpad.com).

## Author contributions

EK and LAR: investigation, methodology, formal analysis, visualization, validation, writing – original draft, writing – review & editing; JÅH: investigation, software; KB: investigation; CK: conceptualization, formal analysis, funding acquisition, investigation, methodology, project administration, resources, software, supervision, visualization, writing – original draft, writing – review & editing.

## Conflicts of interest

There are no conflicts to declare.

## Supplementary Material

MD-015-D3MD00696D-s001
