# Supplementary material for: Structure-based virtual screening of unbiased and RNA-focused libraries to identify new ligands for the HCV IRES model system
Source: RSC Med Chem. 2024 Mar 18;15(5):1527–38. doi: 10.1039/d3md00696d (PMC11110755; doi:10.1039/d3md00696d)
Supplement: MD-015-D3MD00696D-s001 [file MD-015-D3MD00696D-s001.pdf]

Electronic Supplementary Material (ESI) for RSC Medicinal Chemistry.
This journal is © The Royal Society of Chemistry 2024

Supporting Information:

Structure-based virtual screening of unbiased and RNA-focused libraries to identify new ligands for the HCV IRES model system

Elisabeth Kallert,[a,†] Laura Almena Rodriguez,[a,†] Jan-Åke Husmann,[a] Kathrin Blatt,[a] and Christian Kersten[a,b,*]

[a]Institute of Pharmaceutical and Biomedical Sciences, Johannes Gutenberg-University, Staudingerweg 5, 55128 Mainz, Germany.
[b]Institute for Quantitative and Computational Biosciences, Johannes Gutenberg-University, BioZentrum I, Hanns-Dieter-Hüsch.Weg 15, 55128 Mainz, Germany
† EK and LAR contributed equally to this manuscript.
*correspondence CK: kerstec@uni-mainz.de

Table of Contents

1. Figures S1-11 2
2. Tables S1-3 15
3. Extended material and methods: synthesis of compound 5 19
4. Analytical data of virtual screening hits 28
5. References 72

1. Figures S1-11

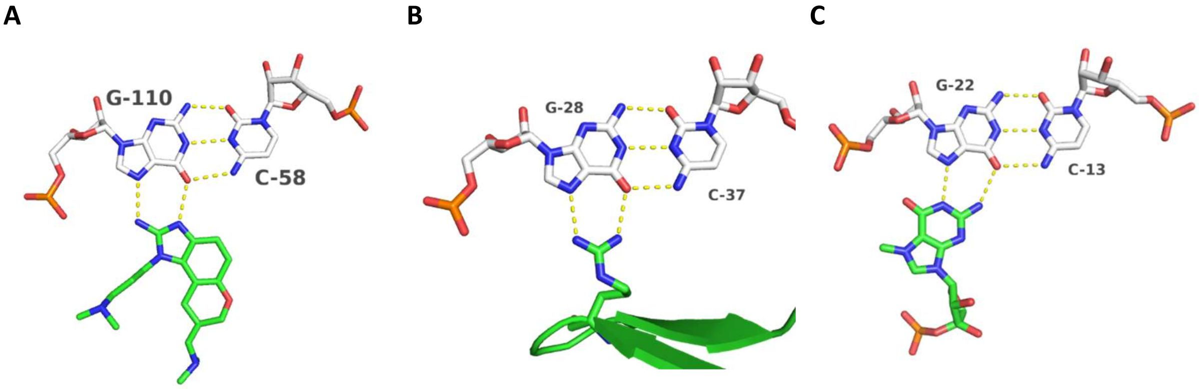

Figure S 1: Hoogsteen edge interactions of G:C pairs (white carbon atoms) with **A)** 2-aminobenzimidazole ligands (HCV IRES, PDB-ID: 3TZR), **B)** arginine (HIV trans-activation response element - peptidic macrocycle complex, PDB-ID 6D2U) and **C)** 7-methylguanosine (HIV reverse-transcription primer tRNA, PDB-ID: 1FIR).

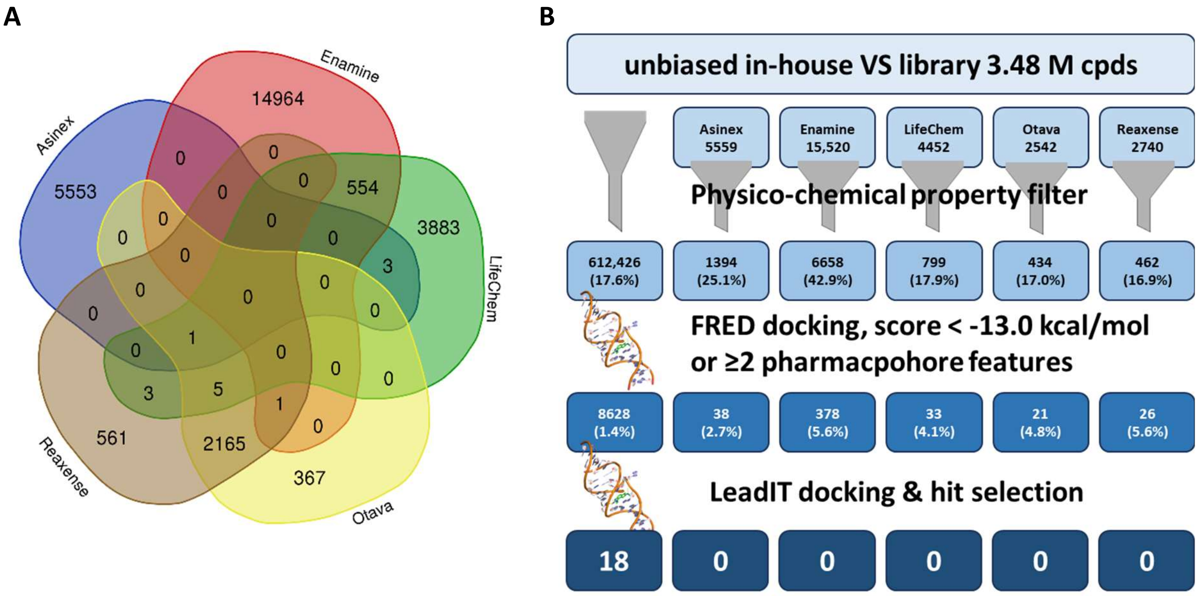

Figure S 2: A) Venn diagram showing little overlap of RNA-focused libraries from different suppliers (except Otava/Reaxense). Figure was made with the Bioinformatics & Evolutionary Genomics web tool (https://bioinformatics.psb.ugent.be/webtools/Venn/). **B)** Schematic representation of virtual screening triage workflow covering physicochemical property filtering, a two-step docking process and human pose inspection for hit selection. Values in parentheses indicate faction of molecules passing the previous step in %.

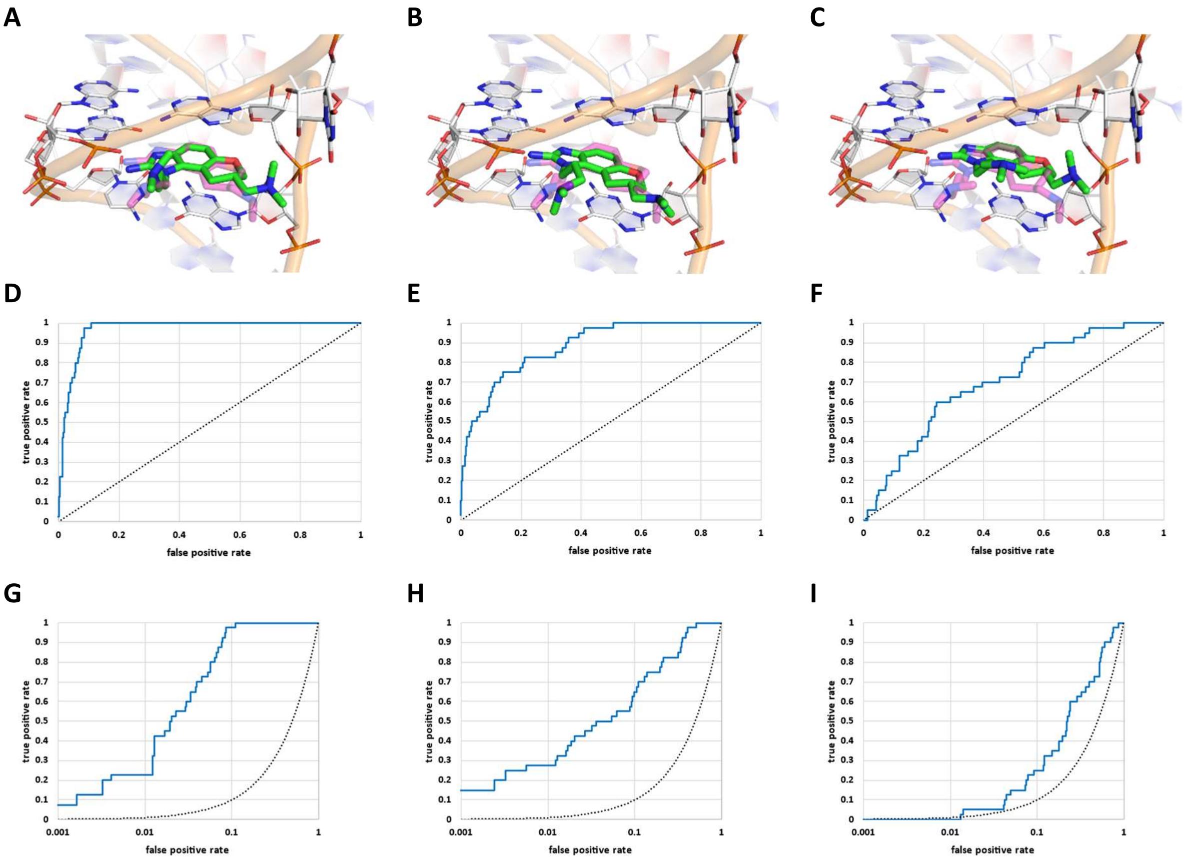

Figure S 3: Re-docking poses of FRED (**A**, RMSD = 1.23 Å, ChemGauss-score = -13.59 kcal/mol), LeadIT (**B**, RMSD = 1.22 Å, FlexX-score = -37.3 kJ/mol) and FlexX (**C**, RMSD = 2.08 Å, FlexX-score = -27.7 kJ/mol). HCV IRES is depicted with white carbon atoms and transparent cartoon (PDB-ID: 3TZR), docking poses with green carbon atoms and the crystallographic reference ligand with transparent, magenta-colored carbon atoms. Receiver operating characteristics curves for FRED (**D**, ROC-AUC = 0.97), LeadIT (**E**, ROC-AUC = 0.88) and FlexX (**F**, ROC-AUC = 0.70). Semilogarithmic ROC curves between 0.1 % and 100 % for FRED (**G**, ROC-logAUC = 0.44), LeadIT (**H**, ROC-logAUC = 0.36) and FlexX (**I**, ROC-logAUC = 0.09). ROC-logAUC values are adjusted to obtain values of 0 for random distribution to 0.855 for prefect discrimination. FlexX was not followed up for the prospective virtual screening.

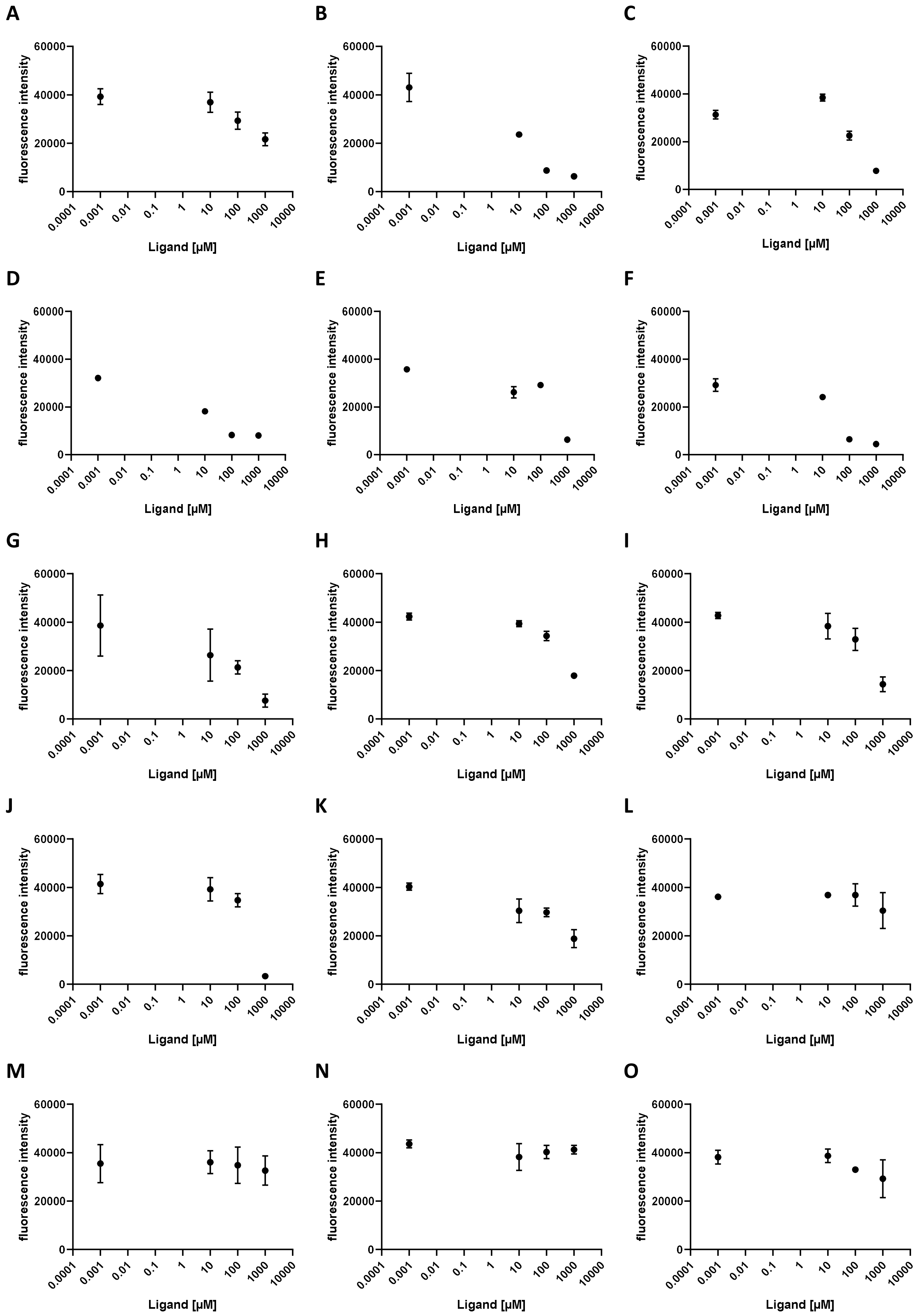
A
B
C
D
E
F
G
H
I
J
K
L
M
N
O
fluorescence intensity
60000
40000
20000
0
0.0001
0.001
0.01
0.1
1
10
100
1000
10000
Ligand [μM]

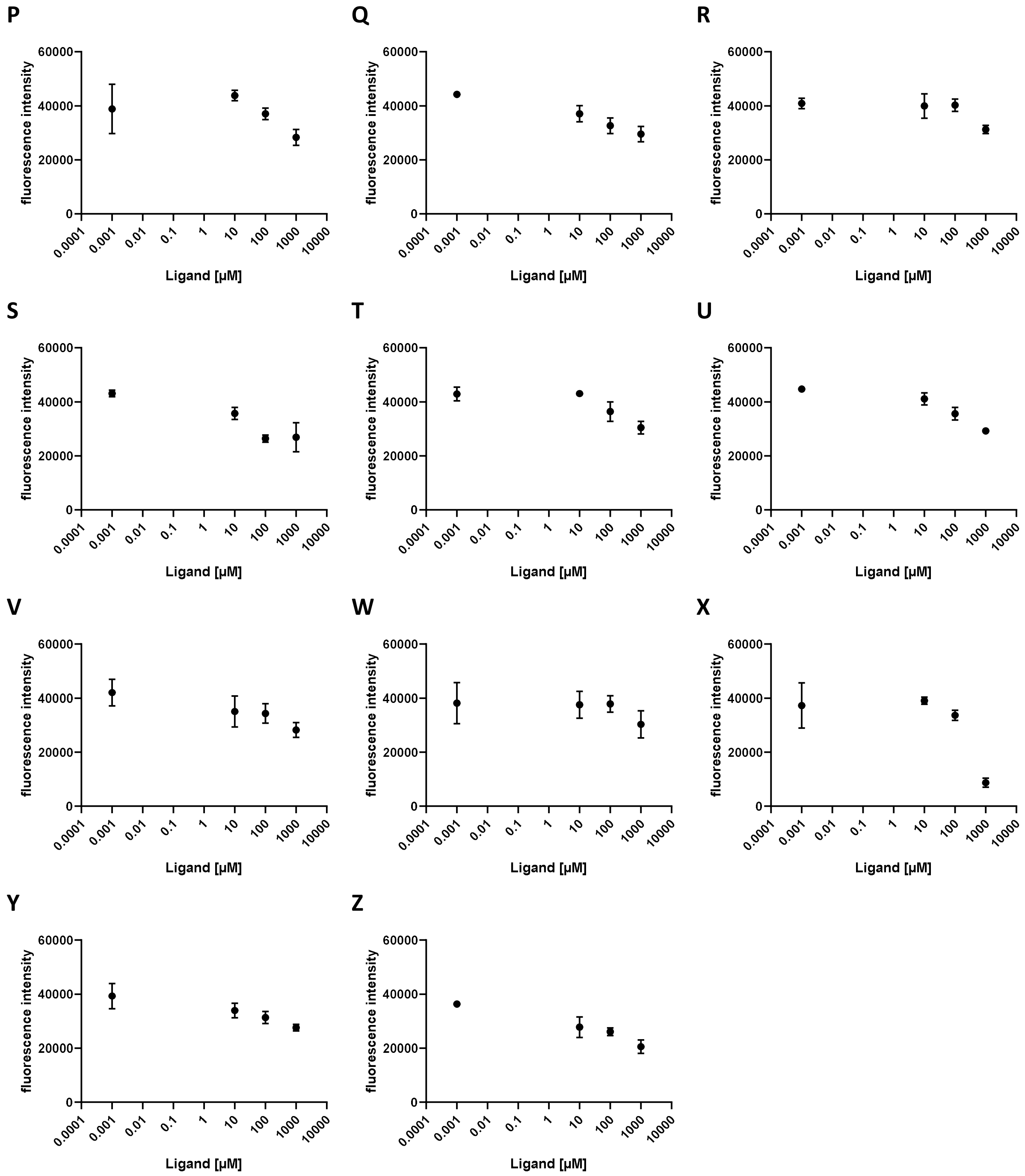

Figure S 4: FRET pre-screening results (Cy5 emission at 670 nm) at concentrations of 1000, 100, 10 and 0.001 µM for compounds **4** (A), **5** (B), **8** (C), **8a** – **c** (D-F), **9** – **28** (G-Z).

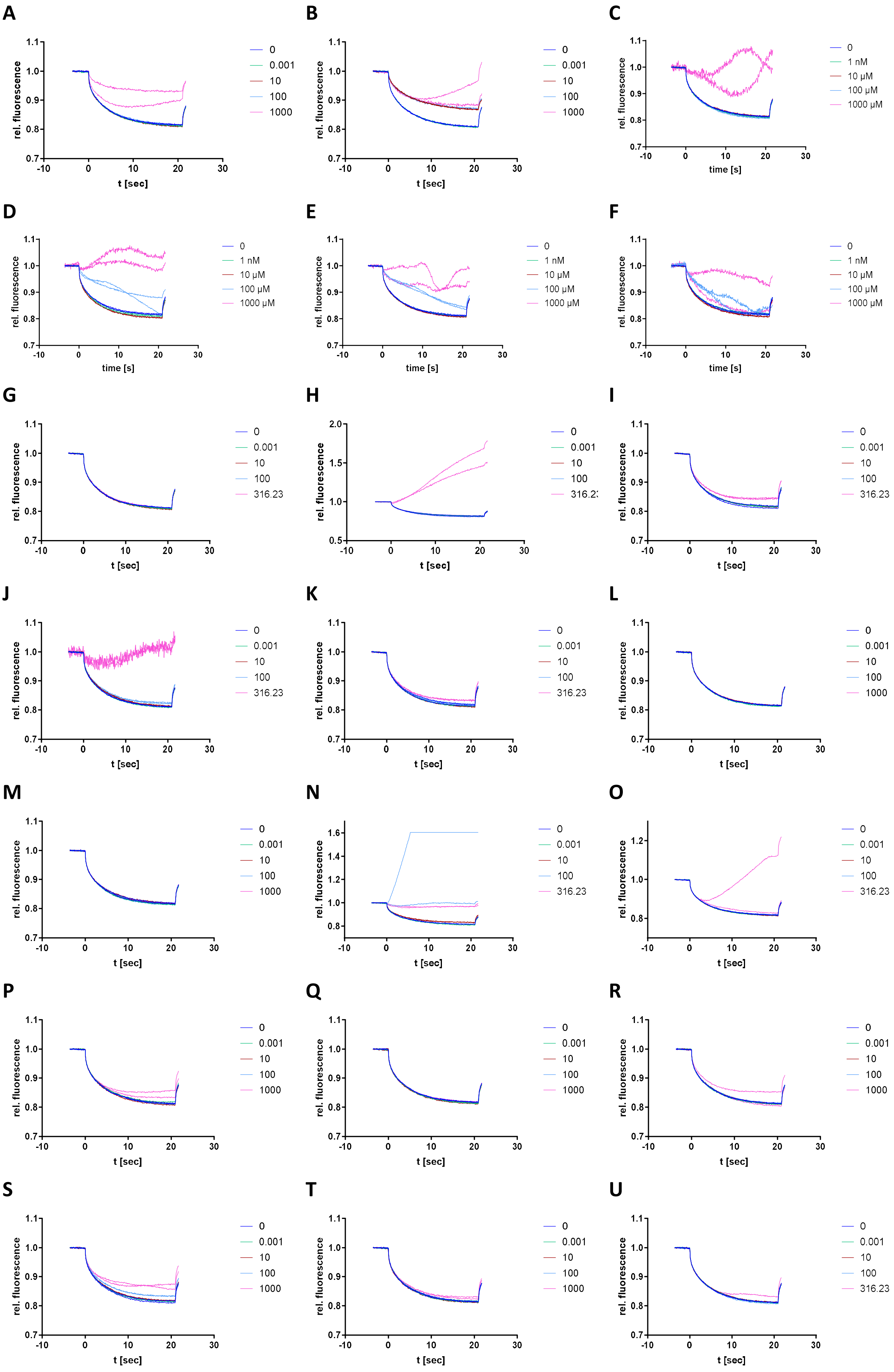
A
rel. fluorescence
t [sec]
0
0.001
10
100
1000
B
rel. fluorescence
t [sec]
0
0.001
10
100
1000
C
rel. fluorescence
time [s]
0
1 nM
10 µM
100 µM
1000 µM
D
rel. fluorescence
time [s]
0
1 nM
10 µM
100 µM
1000 µM
E
rel. fluorescence
time [s]
0
1 nM
10 µM
100 µM
1000 µM
F
rel. fluorescence
time [s]
0
1 nM
10 µM
100 µM
1000 µM
G
rel. fluorescence
t [sec]
0
0.001
10
100
316.23
H
rel. fluorescence
t [sec]
0
0.001
10
100
I
rel. fluorescence
t [sec]
0
0.001
10
100
316.23
J
rel. fluorescence
t [sec]
0
0.001
10
100
316.23
K
rel. fluorescence
t [sec]
0
0.001
10
100
316.23
L
rel. fluorescence
t [sec]
0
0.001
10
100
1000
M
rel. fluorescence
t [sec]
0
0.001
10
100
1000
N
rel. fluorescence
t [sec]
0
0.001
10
100
316.23
O
rel. fluorescence
t [sec]
0
0.001
10
100
316.23
P
rel. fluorescence
t [sec]
0
0.001
10
100
1000
Q
rel. fluorescence
t [sec]
0
0.001
10
100
1000
R
rel. fluorescence
t [sec]
0
0.001
10
100
1000
S
rel. fluorescence
t [sec]
0
0.001
10
100
1000
T
rel. fluorescence
t [sec]
0
0.001
10
100
1000
U
rel. fluorescence
t [sec]
0
0.001
10
100
316.23

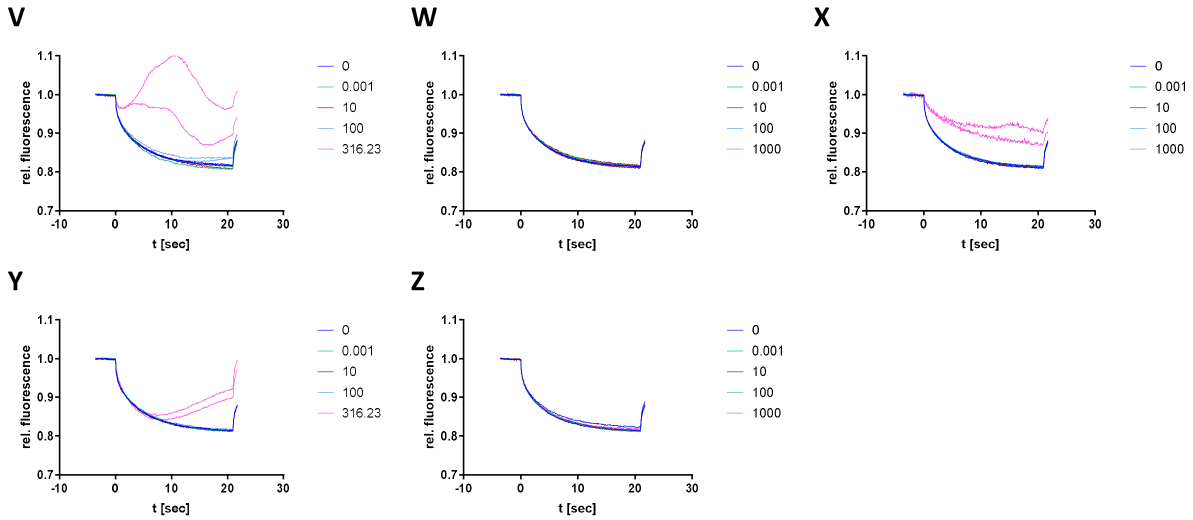

Figure S 5: MST pre-screening results at concentrations of 1000 (316.2 in case of poor solubility), 100, 10 and 0.001 µM for compounds **4** (A), **5** (B), **8** (C), **8a** (D), **8b** (E), **8c** (F), **9** – **28** (G-Z). Aggregation at high concentrations was observed for compounds **5** (B), **8** (C), **8a** (D) **8b** (E), **8c** (F), **10** (H), **12** (J), **16** (N), **17** (O), **24** (V), **26** (X), **27** (Y).

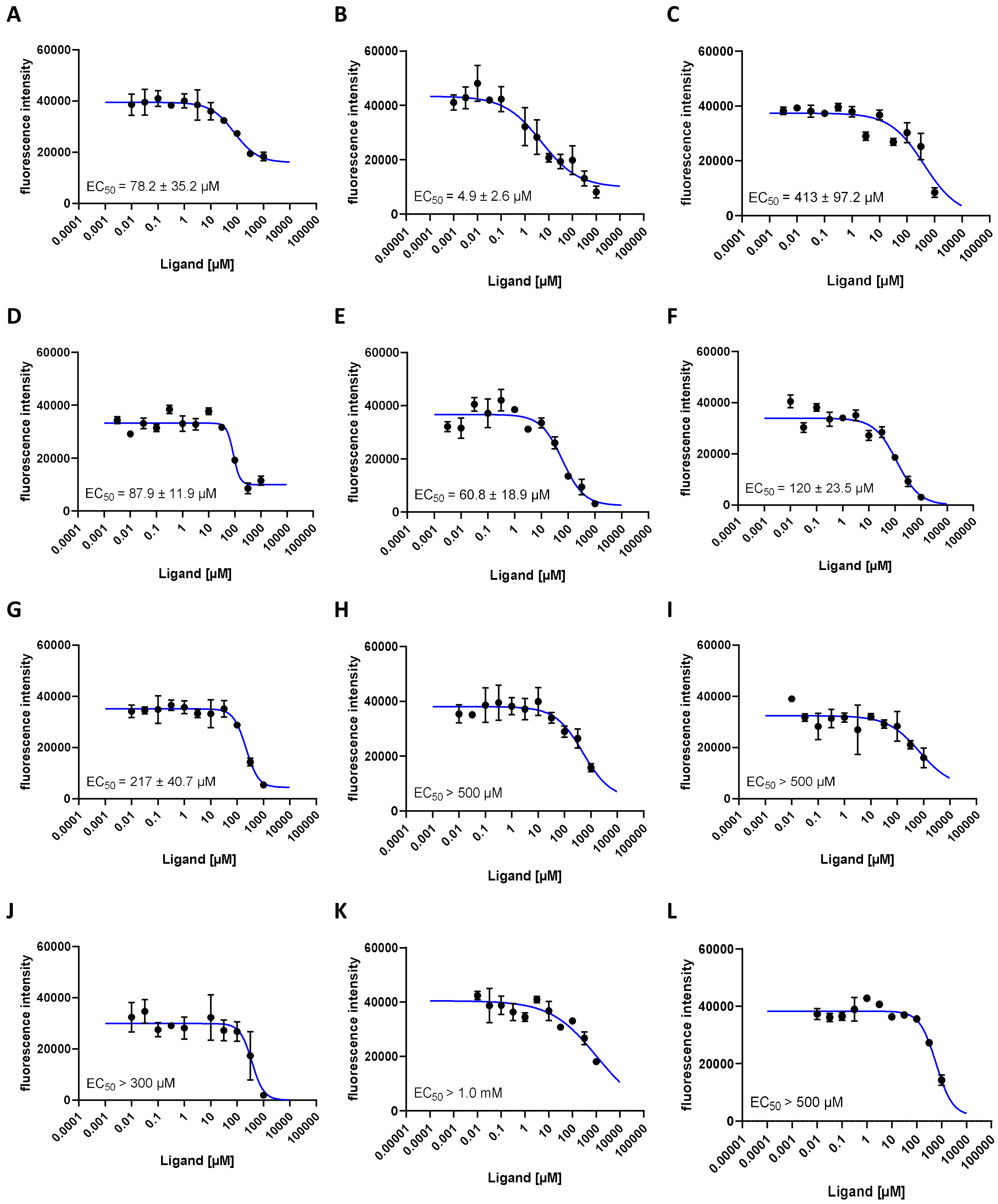

Figure S 6: FRET dose-response curves (Cy5 emission at 670 nm) for compounds **4** (A), **5** (B), **8** (C), **8a** (D), **8b** (E), **8c** (F), **9** (G), **10** (H), **11** (I), **12** (J), **13** (K) and amiloride (**26**, L).

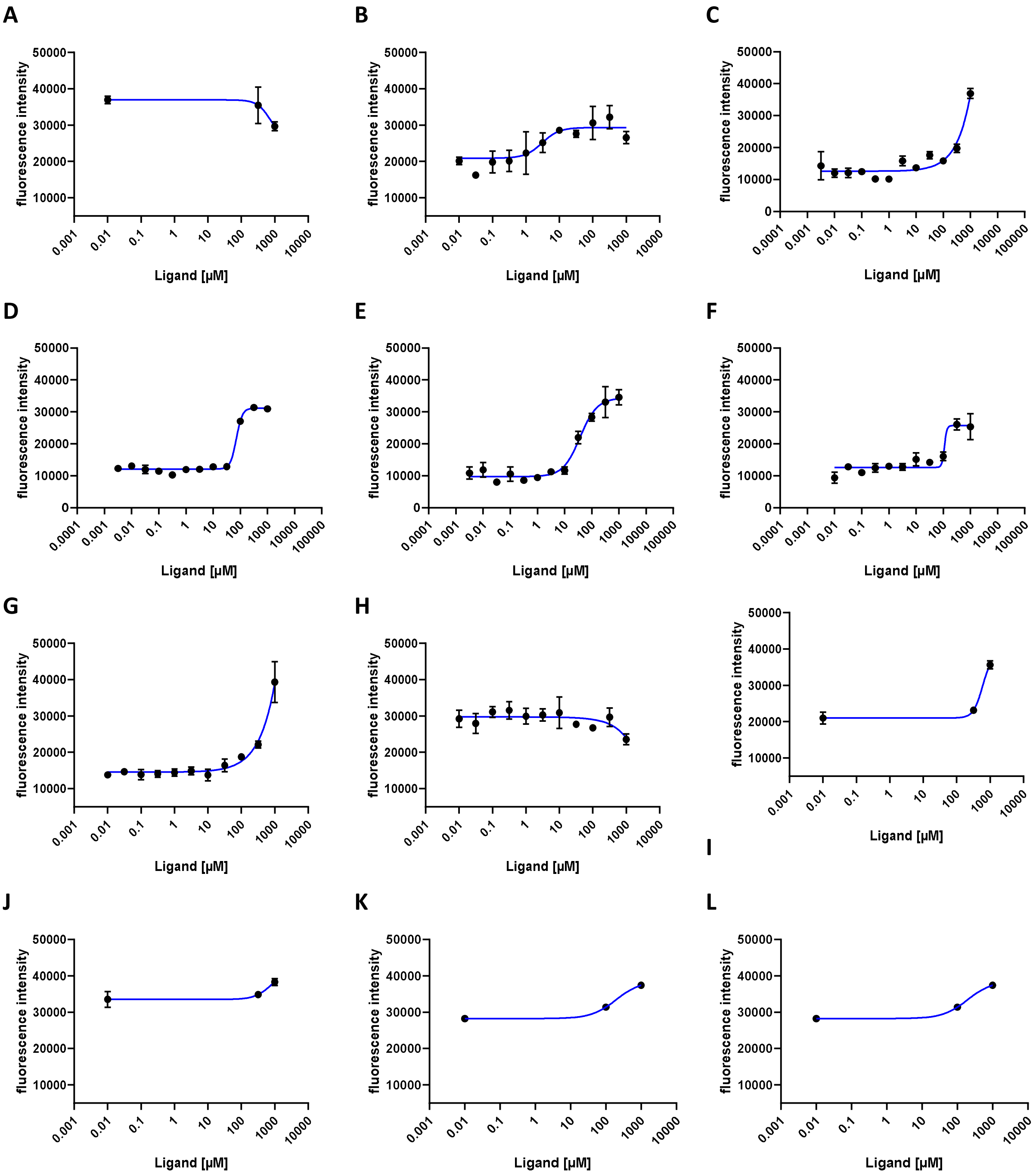

Figure S 7: Cy3 emission fluorescence intensity (535 nm) dose-response curves for compounds **4** (A), **5** (B), **8** (C), **8a** (D), **8b** (E), **8c** (F), **9** (G), **10** (H), **11** (I), **12** (J), **13** (K) and amiloride (**26**, L).

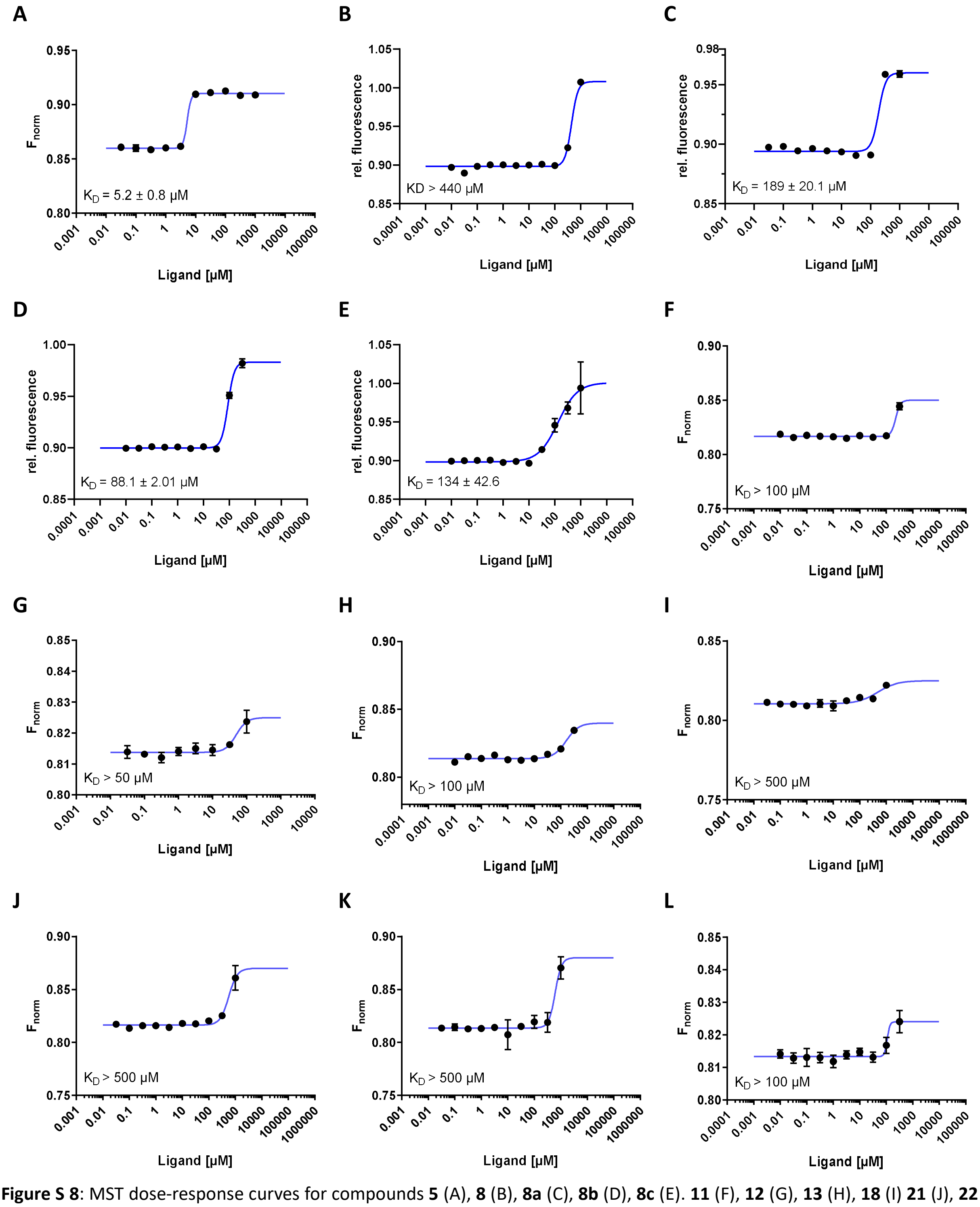

Figure S 8: MST dose-response curves for compounds **5** (A), **8** (B), **8a** (C), **8b** (D), **8c** (E). **11** (F), **12** (G), **13** (H), **18** (I) **21** (J), **22** (K) and amiloride (**26**, L).

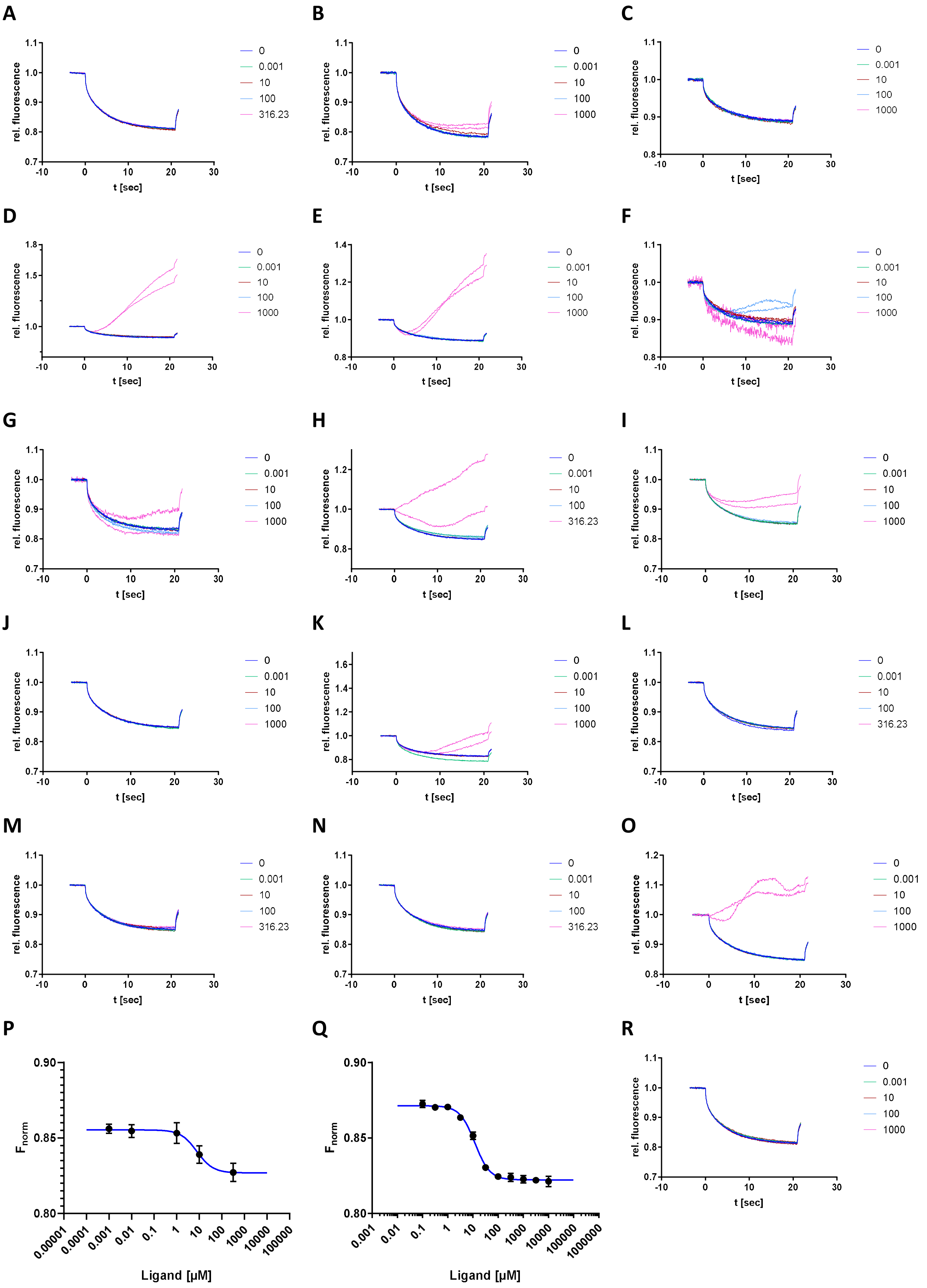
A
B
C
D
E
F
G
H
I
J
K
L
M
N
O
P
Q
R
rel. fluorescence
t [sec]
0
0.001
10
100
316.23
1000
F_{norm}
Ligand [µM]

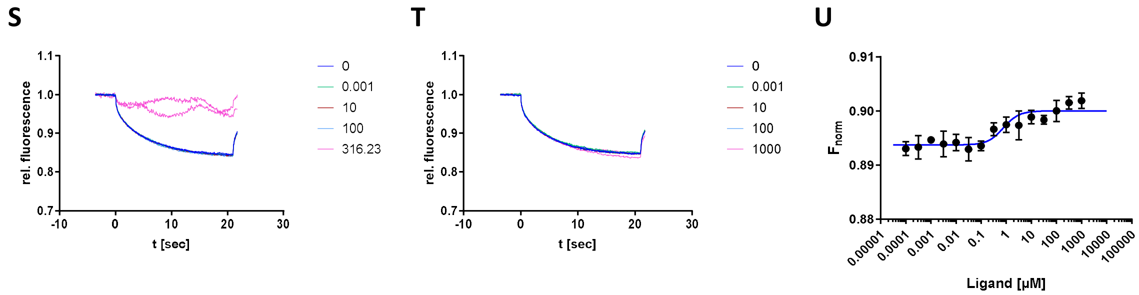

Figure S 9: MST-traces from the selectivity counter-screening against the preQ$_1$-riboswitch. Compound **4** (A), **5** (B), **8** (C), **8a** (D), **8b** (E), **8c** (F), **9** (G), **10** (H), **11** (I), **12** (J), **13** (K), **18** (L), **21** (M), **22** (N) and amiloride (**26**, O). Aggregation was observed for high concentrations of compounds **8a** (D), **8b** (E), **8c** (F), **9** (G), **10** (H), **11** (I), **13** (K) and **26** (O). Weak preQ$_1$-riboswitch binding was observed for compound **5** (B) and **9** (G). P) Dose-response curve of compound **9** binding to the preQ$_1$-riboswitch (K_D = 7.8 µM). Q) Dose-response curve of preQ$_1$ binding to its riboswitch (positive control, K_D = 12 nM). MST-traces of SAM-VI-riboswitch in presence of compound **5** (R), **9** (S) and **28** (T) show no binding. Aggregation was observed for high concentrations of compound **9** (S). U) Dose-response curve of SAM binding to the SAM-VI-riboswitch (positive control, K_D = 772 nM).

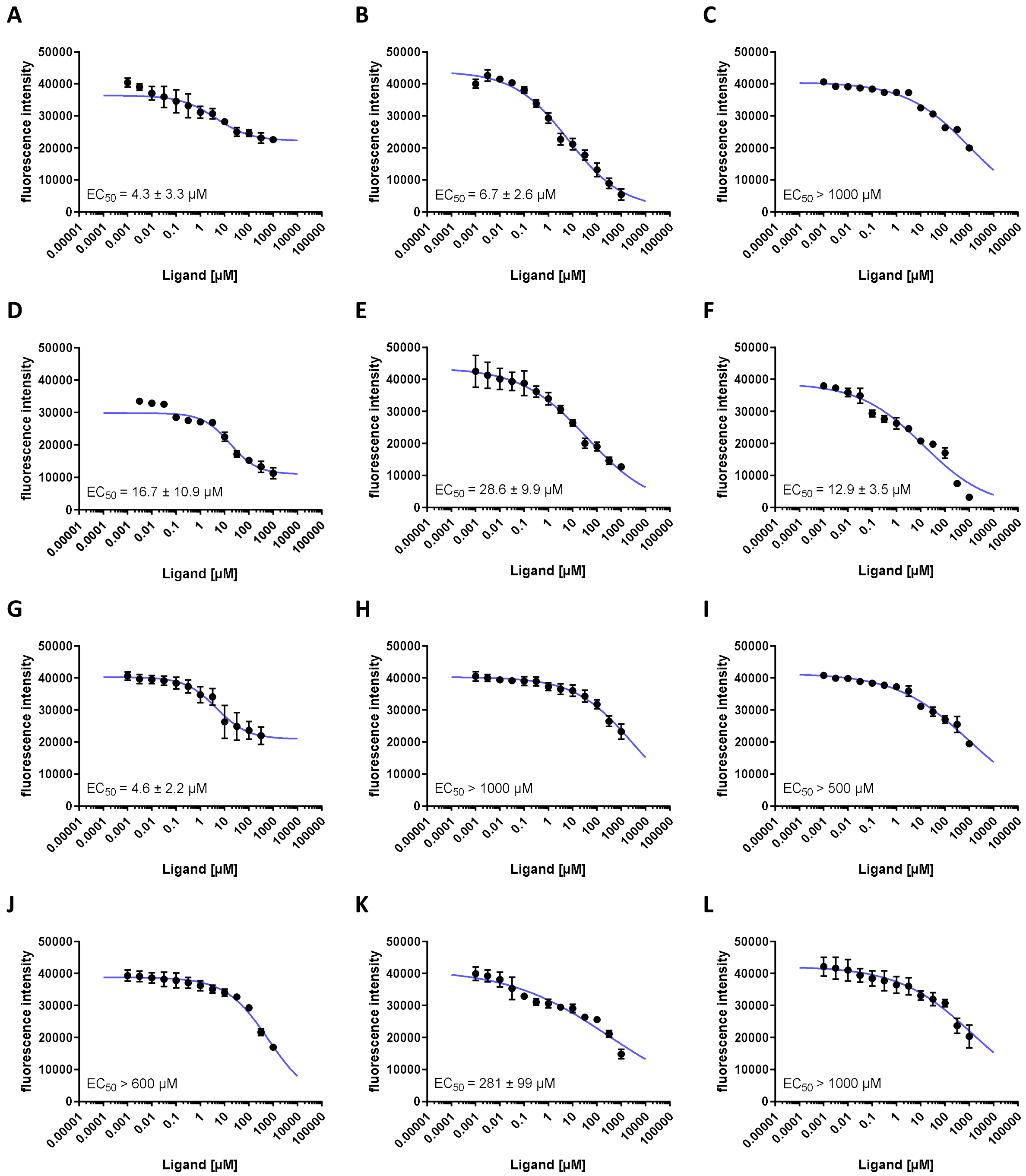

Figure S 10: FRET dose-response curves from the selectivity screening against HCV IRES in presence of total RNA from yeast (1:5 HCV-IRES: total RNA ratio, m/m). Compound **4** (A), **5** (B), **8** (C), **8a** (D), **8b** (E), **8c** (F), **9** (G), **10** (H), **11** (I), **12** (J), **13** (K) and amiloride (**26**, L). For **4** and **9** lower EC_{50}-values are observed in presence of total RNA compared to HCV IRES alone (Figure S6 A and G, respectively), but the remaining fluorescence intensity is higher indicating impairment of the conformational change.

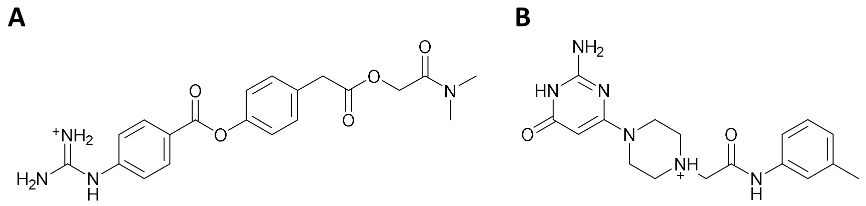

27 **28**

Figure S 11: Molecular structures of camostat mesylate **27** (A) and the previously reported preQ$_1$-riboswitch ligand **28** (B).

2. Tables S1-3

Table S 1: Physico-chemical parameter comparison between the unbiased in-house VS-library, RNA-focussed libraries from different suppliers and R-bind 2.0 (small molecules). Except of the total library size, numbers are mean values. RO5: Lipinski rule-of-five for oral bioavailability, MW: molecular weight (g/mol), HBA: hydrogen bond acceptors, HBD: hydrogen bond donors, tPSA: topological polar surface area (Å²).

parameter	unbiased	Asinex	Enamine	library LifeChem	Otava	Reaxense	R-bind
library size	4,1654169[b]	5559	15,520	4452	2542	2740	67
RO5 violations	0.1	0.3	0.1	0.1	0.1	0.1	0.3
MW	340.4	390.4	344.9	365.7	335.5	338.2	350.1
HBA	5.5	6.8	6.3	6.4	5.2	5.3	5.9
HBD	1.4	2.0	1.9	1.0	1.8	1.8	3.5
logP	2.1	0.8	1.7	2.7	2.4	2.3	0.2
$logD_{7.4}$	2.5	1.7	2.0	2.9	2.7	2.7	1.1
rotatable bonds	5.4	9.1	5.3	5.1	4.9	5.0	4.9
tPSA	68.5	79.2	77.0	68.1	69.0	69.6	79.0
heavy atoms	23.9	28.2	24.6	26.1	23.9	24.0	25.6
acidic centers	0.22	0.03	0.15	0.52	0.16	0.16	0.16
basic centers	0.25	0.64	0.43	0.48	0.32	0.32	1.99
aromatic atoms	11.0	12.0	13.3	12.4	12.9	13.0	13.9
aromatic/heavy	0.45	0.42	0.54	0.47	0.55	0.55	0.54
nitrogens	3.0	4.5	4.4	4.0	2.8	2.9	4.3
oxygens	2.5	2.2	1.8	2.4	2.4	2.4	1.6
stereo centers	0.6	0.8	0.6	0.3	0.3	0.3	0.3
formal charge	0.12	0.55	0.26	0.13	0.20	0.20	1.06
number of rings	3.0	3.7	3.4	3.6	3.3	3.3	3.7

[b]For molecular docking, molecules with less than 2 HBD and without aromatic atoms were removed according to the HCV IRES pharmacophore hypothesis resulting in 3,481,646 molecules.

Table S 2: Applied physico-chemical property filters using FILTER (OMEGA 4.1.0.0: OpenEye Scientific Software, Santa Fe, NM, USA. http://www.eyesopen.com, 2019). PAINs were removed from all libraries in a separate, preceding step.

AGGREGATORS true "Eliminate known aggregators"
MAX_LIPINSKI_ACCEPTORS 10 "Maximum number of oxygen & nitrogen atoms"
MIN_LIPINSKI_DONORS 2 "Minimum number O & N atoms with hydrogens"
MAX_LIPINSKI_DONORS 5 "Maximum number O & N atoms with hydrogens"
MIN_MOLWT 130 "Minimum molecular weight"
MAX_MOLWT 400 "Maximum molecular weight"
MIN_XLOGP -3.0 "Minimum XLogP"
MAX_XLOGP 3 "Maximum XLogP"
MIN_2D_PSA 50.0 "Minimum 2-Dimensional (SMILES) Polar Surface Area"
MAX_2D_PSA 200.0 "Maximum 2-Dimensional (SMILES) Polar Surface Area"
MIN_ROT_BONDS 0 "Minimum number of rotatable bonds"
MAX_ROT_BONDS 8 "Maximum number of rotatable bonds"
MIN_COUNT_FORMAL_CRG 0 "Minimum number formal charges"
MAX_COUNT_FORMAL_CRG 3 "Maximum number of formal charges"
MIN_SUM_FORMAL_CRG 0 "Minimum sum of formal charges"
MAX_SUM_FORMAL_CRG 3 "Maximum sum of formal charges"
MIN_CHIRAL_CENTERS 0 "Minimum chiral centers"
MAX_CHIRAL_CENTERS 3 "Maximum chiral centers"
ELIMINATE_METALS Sc,Ti,V,Cr,Mn,Fe,Co,Ni,Cu,Zn,Y,Zr,Nb,Mo,Tc,Ru,Rh,Pd,Ag,Cd
ALLOWED_ELEMENTS H,C,N,O,F,P,S,Cl,Br,I

Table S 3: Molecular docking results of virtual screening hits selected for testing including identity and purity confirmation by LC-MS analytics (for details see supporting information 4. Analytical data of virtual screening hits). Molecules are depicted as their likely protomeric/tautomeric states under physiological conditions. For **15** – **22** different protomers/tautomers are reasonable. CoA: certificate of analysis.

compound	SMILES	FRED docking score kcal/mol	FRED docking ph4-features	LeadIT docking score kJ/mol	LeadIT docking ph4-features	Analytics mass calc. / mass found	Analytics purity [%]	Analytics Supplier CoA
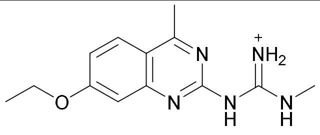 **8**	O(CC)c1cc2nc(NC(=[N+])NC)nc(C)c2cc1	-14.20	2	-28.73	2	260.2 / 260.0 [M + H$^+$]	98.8	identity: ^{1}H NMR purity statement: >90 %
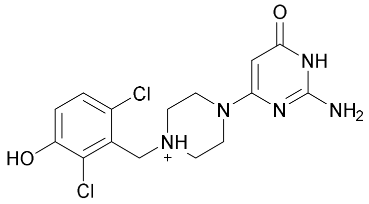 **9**	Clc1c(C[N+]2CCN(C=3N=C(N)NC(=O)C=3)CC2)c(Cl)ccc1O	-13.88	2	-31.82	2	370.1 / 370.0 [M + H$^+$]	93.7	LC-MS (mass: 370.0, purity: 96.6%)
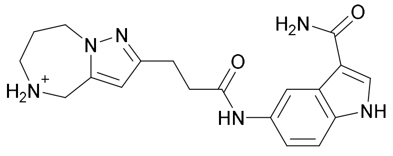 **10**	O=C(Nc1cc2c(C(=O)N)c[nH]c2cc1)CCc1nn2c(c1)C[N+]CCC2	-13.91	1	-39.90	1	367.2 / 367.1 [M + H$^+$]	90.5	LC-MS (mass: 367.1, purity: 95.7%)
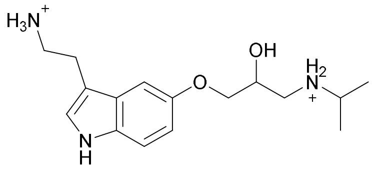 **11**	O(CC(O)C[N+]C(C)C)c1cc2c(CC[N+])c[nH]c2cc1	-15.86	1	-28.57	1	292.2 / 292.1 [M + H$^+$]	99.1	identity: ^{1}H NMR purity statement: >85%
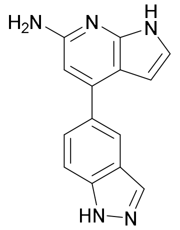 **12**	Nc1nc2[nH]ccc2c(-c2cc3c([nH]nc3)cc2)c1	-15.77	1	-40.28	2	250.1 / 250.0 [M + H$^+$]	93.8	LC-MS (mass: 250.1, purity: 95.4%)
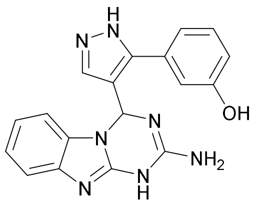 **13**	Oc1cc(-c2c(C3n4c5c(nc4NC(N)=N3)cccc5)cn[nH]2)ccc1	-16.02	1	-34.17	2	346.1 / 346.0 [M + H$^+$]	99.2	identity: ^{1}H NMR purity statement: >90%
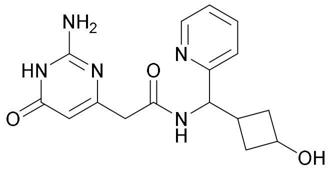 **14**	O=C(NC(c1ncccc1)C1CC(O)C1)CC=1N=C(N)NC(=O)C=1	-14.87	1	-33.61	2	330.2 / 330.0 [M + H$^+$]	98.9	LC-MS (mass: 330.2, purity: 100%)

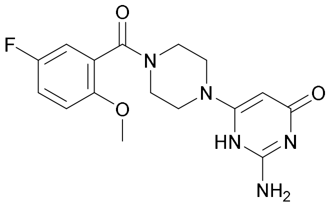 **15**	Fc1cc(C(=O)N2CCN(C=3NC(N)=NC(=O)C=3)CC2)c(OC)cc1	-13.05	1	-32.14	2	348.1 / 348.0 $[M + H^+]$	98.2	LC-MS (mass: 348.0, purity: 100%)
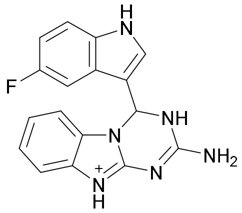 **16**	Fc1cc2c(C3n4c5c([n+]c4N=C(N)N3)cccc5)c[nH]c2cc1	-14.37	3	-30.37	3	321.1 / 321.0 $[M + H^+]$	98.3	identity: ^{1}H NMR purity statement: >85%
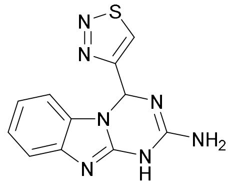 **17**	NC1=NC(c2nnsc2)n2c3c(nc2N1)cccc3	-13.47	3	-31.02	2	272.3 $[M + H^+]$ / 310.0 $[M + K^+]$	97.9	LC-MS (mass: 272.2, purity: 100%)
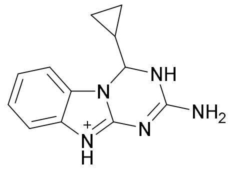 **18**	NC1=Nc2[n+]c3c(n2C(C2CC2)N1)cccc3	-12.17	3	-29.74	2	228.1 / 228.0 $[M + H^+]$	98.6	LC-MS (mass: 228.2, purity: 99.1%)
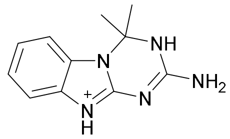 **19**	NC1=Nc2[n+]c3c(n2C(C)(C)N1)cccc3	-13.47	1	-29.81	3	216.1 / 216.0 $[M + H^+]$	100.0	identity: ^{1}H NMR purity statement: >90 %
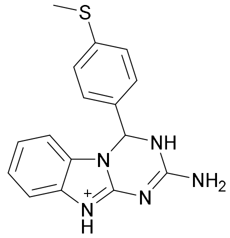 **20**	S(C)c1ccc(C2n3c4c([n+]c3N=C(N)N2)cccc4)cc1	-11.54	1	n.d.	n.d.	310.4 / 310.0 $[M + H^+]$	97.8	LC-MS (mass: 310.2, purity: 100%)
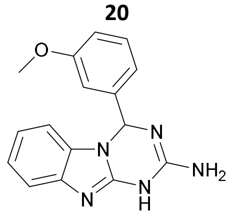 **21**	O(C)c1cc(C2n3c4c(nc3NC(N)=N2)cccc4)ccc1	-11.27	1	n.d.	n.d.	294.1 / 294.0 $[M + H^+]$	99.7	LC-MS (mass: 294.1 , purity: 100%)
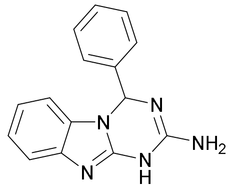 **22**	NC1=NC(c2ccccc2)n2c3c(nc2N1)cccc3	-11.26	3	-29.04	0	264.1 / 264.0 $[M + H^+]$	97.9	LC-MS (mass: 264.0, purity: 97.7%)

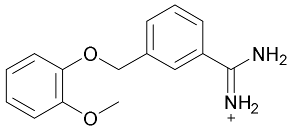 **23**	O(Cc1cc(C(=[N+])N)ccc1)c1c(OC)cccc1	-13.64	1	-27.57	2	257.1 / 257.0 [M + H$^+$]	99.8	LC-MS (mass: 257.1, purity: 100%)
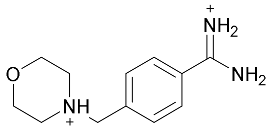 **24**	[N+]=C(N)c1ccc(C[N+]2CCOCC2)cc1	-13.64	2	-28.71	2	220.2 / 220.0 [M + H$^+$]	97.6	LC-MS (mass: 220.0, purity statement: >90 %)
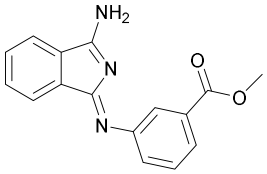 **25**	O=C(OC)c1cc(N=C2N=C(N)c3c2cccc3)ccc1	-14.53	3	-29.00	1	280.1 / 280.0 [M + H$^+$]	92.6	identity: ^{1}H NMR purity statement: >85%

3. Extended material and methods: synthesis of compound 5

General experimental methods

All reagents and solvents were commercial grade and used without further purification. Reaction progress was monitored by thin layer chromatography (**TLC**) using Alugram Xtra F254 silica plates from Machery-Nagel or neutral aluminium oxide 60 F254 plates from Merck KGaA. In addition, high-performance liquid chromatography/electron spray ionization mass spectrometry (**HPLC/ESI-MS**) was used to control reaction's conversion and to determine the identity as well as the purity of compounds tested in the assay. An Agilent 1100 series HPLC system and either an Agilent Zorbax SB-Aq (4.6 mm x 150 mm, 5 µm) or Poroshell 120 EC-C18 (2.10 mm x 150 mm, 4 µM) column coupled to an Agilent 1100 series LC/MSD Trap with ESI, was used. The measurements were conducted with a gradient of acetonitrile and water (+0.1% formic acid) with 10%–90% acetonitrile over 10 min with a flow rate of 0.7 mL/min. Signals were detected at 254 nm with quantification by area under curve (AUC) and masses were determined in positive ionization mode (ESI) unless otherwise stated. **Column chromatography** was performed with silica gel 60 (40–63 µm) from Machery-Nagel. **Flash column chromatography** was performed with the Biotage Isolera™ One system. For reversed-phase flash chromatography prepacked columns of the type Biotage®Sfär C18 Duo from Biotage® were used. In the case of normal-phase flash chromatography, columns were self-packed with silica gel 60 (15–40 µm) from Merck KGaA. **Preparative HPLC** purification was performed with an Agilent 1290 II Infinity Preparative LC System using a MZ-Aqua Perfect C18 20 x 250 mm, 7 µm preparative LC column and acetonitrile/water +0.1% formic acid as mobile phase. **Melting points** (uncorrected) were measured with an MPM-H3 using semi-open capillaries. Nuclear magnetic resonance (**NMR**) spectra were recorded as stated individually on Bruker Fourier 300 MHz or Bruker Avance III 600 MHz. The chemical shift was abbreviated to δ and has the unit ppm. Tetramethyl silane (TMS) (δ = 0 ppm) was used as the reference substance. The chemical shifts were referenced to the solvent peaks in ^{1}H: δ = 7.26 (Chloroform-*d*), 2.50 (DMSO-*d*6) ppm and in ^{13}C: δ = 77.16 (Chloroform-*d*), 39.52 (DMSO-d6) ppm purchased from Deutero GmbH. In addition, the following abbreviations for the multiplicities of the peaks were defined: s (singlet), d (doublet), dd (doublet of doublet), t (triplet), td (triplet of doublet), q (quartet), qd (quartet of doublet), h (sextet), hept (septet) and m (multiplet). In the evaluation of the 1H-NMR spectrum, the coupling constant *J* was given in Hz and denoted together with the number of signaling hydrogen atoms. The MestReNova 12.0.4-22023 NMR spectrum processing program from *Mestrelab Research* was used to evaluate the NMR spectra and to determine the purity of the compound through LC-MS.

Overview

The following synthetic route is based on previous reported procedures[1,2], which were slightly modified at some points.

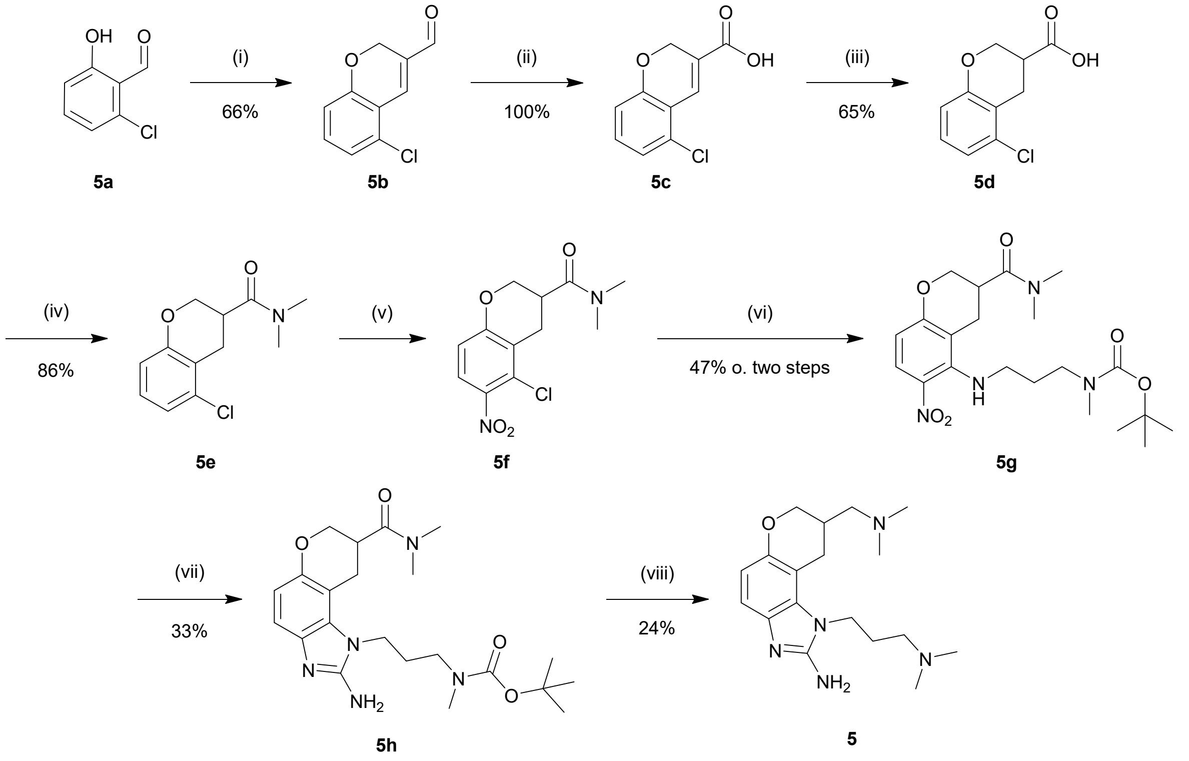

Scheme 1: Reagents and conditions: (i) Acrolein, DABCO, dioxane, 95 °C; (ii) silver nitrate, NaOH, EtOH/H_2O, 85° C; (iii) Na/Hg, NaOH/H_2O, room temperature (rt); (iv) EDC HCl, HOBt, dimethylamine hydrochloride, *N*-Methyl morpholine, dichloromethane, rt; (v) sodium nitrate, trifluoro acetic acid, dichloroacetic acid, 0 °C-rt; (vi) *N*-(3-aminopropyl)-*N*-methylcarbamate, 1-methyl-2-pyrrolidinone, 75 °C; (vii) H_2 Pd/C, EtOH, then cyanogen bromide/EtOH, rt; (viii) LAH, THF, 60 °C.

5-Chloro-2*H*-chromene-3-carbaldehyde (5b)

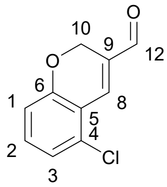

A mixture of 6-chlorosalicylaldehyde (**5a)** (4.84 g, 30.9 mmol, 1.00 equiv), acrolein (2.89 g, 3.44 mL, 46.4 mmol, 1.50 equiv) and 1,4-diazabicyclo[2.2.2]octane (1.73 g, 15.5 mmol, 0.500 equiv) in dioxane (16.9 mL) was heated under inert gas atmosphere at 95 °C for 2 h in a sealed flask. The reaction mixture was cooled to room temperature and diluted with dichloromethane (75.0 ml). The organic phase was washed with 3M HCl (3 x 30.0 mL). The aqueous layer was extracted with dichloromethane (3 x 30.0 mL). Combined organic layers were washed with aqueous saturated sodium chloride solution, dried over sodium sulfate, filtered, and concentrated under reduced pressure. Crude product was purified *via* column chromatography (isocratic, cyclohexane (Cy): ethyl acetate (EA); 50:1). Target compound **5b** (3.95 g, 20.3 mmol, **66% yield**) was isolated as yellow solid. $\boldsymbol{T_m}$ = 60–62 °C. **^{1}H-NMR, COSY** (300 MHz, Chloroform-*d*, TMS) δ_H 9.63 (1H, s, 12-H), 7.59 (1H, q, *J* = 1.3 Hz, 8-H), 7.19 (1H, t, *J* = 8.1 Hz, 2-H), 6.99 (1H, dd, *J* = 8.1, 1.0 Hz, 1-H), 6.78 (1H, dt, *J* = 8.1, 1.0 Hz, 3-H), 4.99 (2H, d, *J* = 1.3 Hz, 10-H). **^{13}C-NMR, HSQC, HMBC** (75 MHz, Chloroform-*d*, TMS) δ_C 189.83 (C-12), 157.21 (C-6), 137.18 (C-8), 133.95 (C-4), 133.14 (C-2), 132.42 (C-9), 122.74 (C-1), 119.37 (C-5), 115.45 (C-3), 63.13 (C-10). **MS (ESI)** found: m/z = 194.9 [M+H$^+$], calculated: m/z = 195.0 [M+H$^+$].

5-Chloro-2*H*-chromene-3-carboxylic acid (5c)

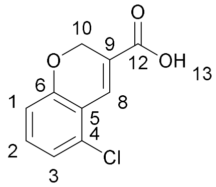

A solution of sodium hydroxide (3.25 g, 81.2 mmol, 4.00 equiv) in water (25.7 mL) was added to absolute ethanol (52.1 mL). A solution of silver nitrate (7.24 g, 42.6 mmol, 2.10 equiv) in water (25.7 mL) was added portionwise. Then **5b** (3.95 g, 20.3 mmol, 1.00 equiv) was added and the mixture was heated at 85°C for 75 min. The supernatant was decanted after cooling down at room temperature and the solid was washed with a 1:1 ethanol/water solution (4 × 20.0 mL). The washings were combined with the decanted supernatant and were diluted with an excess of 1M aqueous HCl (150 mL) resulting in a voluminous light-yellow precipitate. The aqueous suspension was extracted with methylene chloride (3 x 75.0 mL). Combined organic layers were dried over sodium sulfate, filtered, and concentrated under reduced pressure. Target compound **5c** (4.26 g, 20.2 mmol, **100% yield**) was isolated as light-yellow solid. $\boldsymbol{T_m}$ = 190–192 °C. **^{1}H-NMR, COSY** (300 MHz, DMSO-*d*$_6$, TMS) δ_H 7.53 (1H, q, *J* = 1.5 Hz, 8-H), 7.27 (1H, t, *J* = 8.1 Hz, 2-H), 7.08 (1H, dd, *J* = 8.1, 1.1 Hz, 1-H), 6.86 (1H, dt, *J* = 8.1, 1.1 Hz, 3-H), 4.92 (2H, d, *J* = 1.5 Hz, 10-H). **^{13}C-NMR, HSQC, HMBC** (75 MHz, DMSO-*d*$_6$, TMS) δ_C 165.09 (C-12), 155.70 (C-6), 132.36 (C-2), 132.08 (C-4), 127.34 (C-8), 125.43 (C-9), 122.39 (C-1), 118.99 (C-5), 115.12 (C-3), 64.09 (C-10). **MS (ESI)** found: m/z = 210.9 [M+H$^+$], calculated: m/z = 211.0 [M+H$^+$].

5-Chlorochromane-3-carboxylic acid (5d)

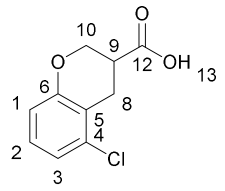

To a solution of **5c** (4.25 g, 20.2 mmol, 1.00 equiv) in 10% aqueous sodium hydroxide solution (100 mL) was added portionwise 20% sodium amalgam (8.80 g) over a period of 30 min (caution: gas and heat evolution). The grey suspension was stirred for 3 h at room temperature. Afterwards, the progress of the reaction was controlled via TLC. If starting material is left, an appropriate amount of 20% sodium amalgam was added portionwise (additional total amount of 20% sodium amalgam of 6.20 g was added in four portions). This process was repeated till reaction's completion, which resulted in a white suspension. The supernatant was decanted from the liquid mercury, and the mercury was washed with 10% aqueous sodium hydroxide solution (3 x 20.0 mL). The combined supernatants were acidified to a pH of 2 with concentrated hydrochloric acid, and extracted with dichloromethane (3 x 50.0 mL). Combined organic layers were dried over sodium sulfate, filtered, and concentrated under reduced pressure. Target compound **5d** (2.80 g, 13.2 mmol, **65% yield**) was isolated as colorless solid. $\boldsymbol{T_m}$ = 190–192 °C. **^{1}H-NMR, COSY** (300 MHz, DMSO-d_6, TMS) δ_H 12.69 (1H, br s, 13-H), 7.11 (1H, t, J = 8.1 Hz, 2-H), 7.00 (1H, dd, J = 8.1, 1.3 Hz, 1-H), 6.77 (1H, dd, J = 8.1, 1.3 Hz, 3-H), 4.33 – 4.12 (2H, m, 10-H), 3.07 (1H, tdd, J = 7.1, 6.7, 3.4 Hz, 9-H), 2.91 (2H, d, J = 6.7 Hz, 8-H). **^{13}C-NMR, HSQC, HMBC** (75 MHz, DMSO-d_6, TMS) δ_C 173.04 (C-12), 155.35 (C-6), 133.68 (C-4), 127.79 (C-2), 120.97 (C-1), 119.78 (C-5), 115.32 (C-3), 66.07 (C-10), 37.20 (C-9), 24.89 (C-8). **MS (ESI)** (in negative mode) found: m/z = 210.7 [M-H$^-$], calculated: m/z = 211.0 [M-H$^-$].

5-Chloro-*N,N*-dimethylchromane-3-carboxamide (5e)

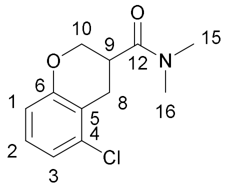

To a solution of **5d** (1.40 g, 6.58 mmol, 1.00 equiv), 1-ethyl-3-(3-dimethylaminopropyl)carbodiimide hydrochloride (1.89 g, 9.88 mmol, 1.50 equiv), dimethylamine hydrochloride (1.34 g, 16.5 mmol, 2.50 equiv) and *N*-hydroxy benzotriazole (1.51 g, 9.88 mmol, 1.50 equiv) in dichloromethane (60.0 mL) was added 4-methylmorpholine (3.33 g, 3.66 mL, 32.9 mmol, 5.00 equiv) under inert gas atmosphere and the mixture stirred for 66 h at room temperature. The reaction mixture was diluted with additional dichloromethane, and an equal volume of saturated aqueous sodium bicarbonate solution was added. The organic phase was separated and the aqueous phase was washed with dichloromethane (3x 25.0 mL). The combined organic phases were washed with aqueous saturated sodium chloride solution (1 x 30.0 mL), dried over sodium sulfate, filtered, and concentrated under reduced pressure. The crude product was purified *via* flash chromatography (gradient 0–25% EA in Cy; product eluates at 15% EA) and lyophilized afterwards. Target compound **5e** (1.36 g, 5.66 mmol, **86% yield**) was isolated as colorless oil. **^{1}H-NMR, COSY** (300 MHz, DMSO-d_6, TMS) δ_H 7.12 (1H, t, J = 8.0 Hz, 2-H), 7.00 (1H, d, J = 8.0 Hz, 1-H), 6.79 (1H d, J = 8.0 Hz, 3-H), 4.31 (1H, d, J = 10.3 Hz, 10-H), 3.84 (1H, t, J = 10.3 Hz, 10-H), 3.40 – 3.25 (1H, m, 9-H), 3.10 (3H, s, 15/16-H), 2.95 – 2.67 (5H, m, 15/16-H, 8-H). **^{13}C-NMR, HSQC, HMBC** (75 MHz, DMSO-d_6, TMS) δ_C 170.83 (C-12), 155.25 (C-6), 133.66 (C-4), 127.70 (C-2), 120.80 (C-

1), 120.19, (C-5) 115.19 (C-3), 66.45 (C-10), 36.74 (C-15/16), 35.07 (C-15/16), 34.15 (C-9), 26.18 (C-8). **MS (ESI)** found: m/z = 240.0 [M+H$^+$], calculated: m/z = 240.1 [M+H$^+$].

5-Chloro-*N,N*-dimethyl-6-nitrochromane-3-carboxamide (5f)

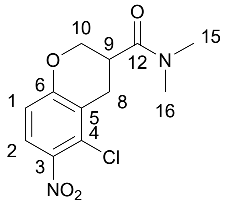

Pestled sodium nitrate (2.89 g, 34.0 mmol, 3.00 equiv) and **5e** (2.72 g, 11.3 mmol, 1.00 equiv) were mixed in dichloroacetic acid (140 mL) under ice cooling and inert gas atmosphere. Trifluoroacetic acid (465 mg, 312 µL, 4.08 mmol, 0.360 equiv) was added and the mixture stirred for 28 h covering the ice bath and allowing it to warm up overnight. The resulting dark crude reaction mixture was then slowly poured into a rapidly stirred solution of sodium carbonate (10%, 1.20 L) (Caution CO2 evolution). After rapid stirring for 10 minutes, the aqueous phase was extracted with dichloromethane (3 x 75.0 mL). Combined organic layers were dried over sodium sulfate, filtered, and concentrated under reduced pressure. Crude product of **5f** (red resin-like oil, 1.57 g) was used without further purification. For analytics a small portion was purified *via* column chromatography (isocratic, Cy:EA; 3:2) to separate the regioisomers. Para-substituted isomer eluates first, then product. Major isomer: **5-chloro-*N,N*-dimethyl-6-nitrochromane-3-carboxamide (5f)** light yellow solid. T_m = 126–128 °C. **^{1}H-NMR, COSY** (300 MHz, DMSO-d_6, TMS) δ_H 7.87 (1H, d, J = 9.0 Hz, 2-H), 6.98 (1H, d, J = 9.0 Hz, 1-H), 4.47 – 4.34 (1H, m, 10-H), 4.08 – 3.95 (1H, m, 10-H), 3.47 – 3.36 (1H, m, 9-H), 3.11 (3H, s, 15/16-H), 3.04 – 2.77 (5H, m, 15/16/H, 8-H). **^{13}C-NMR, HSQC, HMBC** (75 MHz, DMSO-d_6, TMS) δ_C 170.38 (C-12), 158.18 (C-6), 141.14 (C-3), 126.93 (C-4), 124.59 (C-2), 122.79 (C-5), 115.47 (C-1), 66.94 (C-10), 36.77 (C-15/16), 35.12 (C-15/16), 33.49 (C-9), 26.58 (C-8). **MS (ESI)** found: m/z= = 285.0 [M+H$^+$], calculated: m/z = 285.1 [M+H$^+$]. Undesired isomer: 5-chloro-*N,N*-dimethyl-8-nitrochromane-3-carboxamide: **^{1}H-NMR, COSY** (300 MHz, DMSO-d_6, TMS) δ_H 7.79 (1H, d, J = 8.8 Hz), 7.21 (1H, d, J = 8.8 Hz), 4.60 – 4.35 (1H, m), 4.17 – 3.94 (1H, m), 3.54 – 3.39 (1H, m), 3.11 (3H, s), 3.01 – 2.71 (5H, m). **^{13}C-NMR, HSQC, HMBC** (75 MHz, DMSO-d_6, TMS) δ_C 170.23, 148.72, 138.42, 137.37, 123.79, 123.49, 120.41, 67.40, 36.76, 35.13, 33.19, 26.54.

Tert-butyl(3-((3-(dimethylcarbamoyl)-6-nitrochroman-5-yl)amino)propyl)(methyl) carbamate (5g)

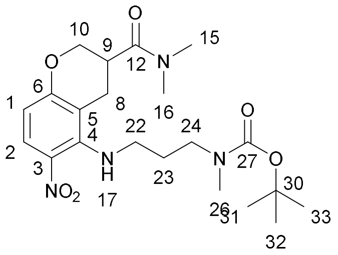

Crude product of **5f** (600 mg, 2.11 mmol, 1.00 equiv) and *N*-(3-aminopropyl)-*N*-methylcarbamate (1.98 g, 10.5 mmol, 5.00 equiv) in 1-methyl-2-pyrrolidinone (2.00 mL) were mixed in a sealed tube under inert gas atmosphere. The mixture was heated at 75 °C for 24 h. After cooling down, the reaction mixture was diluted with diethyl ether (100 mL) and washed with water (4 × 100 mL) and 5% aqueous potassium biphosphate solution (80 mL). The organic phase was dried with sodium sulfate, filtered and concentrated under reduced pressure. Crude product was purified *via* column chromatography (first

isocratic Cy:EA; 1:1, when product starts to eluate percentage of EA can be increased). Target compound **5g** (430 mg, 985 µmol, **47% yield** over two steps) was isolated as bright yellow oil. **^{1}H-NMR, COSY** (300 MHz, Chloroform-*d*) δ_H 7.88 (1H, d, *J* = 9.4 Hz, 2-H), 6.28 (1H, d, *J* = 9.4 Hz, 1-H), 4.40 – 4.28 (1H, m, 10-H), 4.11 – 3.97 (1H, m, 10-H), 3.45 – 3.27 (1H, m, 24-H), 3.27 – 3.12 (2H, m, 22-H), 3.11 – 3.01 (2H, m, 9-H, 24-H), 3.01 – 2.84 (7H, m, 15-H, 16-H, 8-H), 2.83 – 2.60 (4H, m, 8-H, 26-H), 1.73 (2H, p, *J* = 6.9 Hz, 23-H), 1.36 (9H, s, 31-33-H). **^{13}C-NMR, HSQC, HMBC** (75 MHz, Chloroform-*d*) δ_C 171.30 (C-12), 160.03 (C-6), 155.63 (C-27), 148.70 (C-4), 131.40 (C-3), 126.31 (C-2), 110.94 (C-5), 109.24 (C-1), 79.44 (C-30), 67.28 (C-10), 46.17 (C-22), 45.69 (C-24), 37.24 (C-9), 35.62 (C-15/16), 35.15 (C-15/16), 34.29 (C-26), 28.36 (C-23), 27.77 (C-31-33). **MS (ESI)** found: m/z = 437.1 [M+H$^+$], calculated: m/z = 437.2 [M+H$^+$].

***Tert*-butyl (3-(2-amino-8-(dimethylcarbamoyl)-8,9-dihydrochromeno [5,6-*d*] imidazol-1(7*H*)-yl)propyl)(methyl)carbamate (5h)**

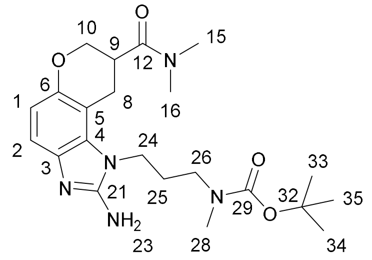

A solution of **5g** (130 mg, 298 µmol, 1.00 equiv) in absolute ethanol (13.5 mL) was mixed with 10% palladium on carbon (135 mg, 127 µmol, 0.425 equiv) under inert gas atmosphere. After addition, the atmosphere was replaced by a hydrogen atmosphere (1 atm) and the mixture stirred for 2 h at room temperature. The mixture was purged with argon upon hydrogen atmosphere removal. Afterwards, the mixture was quickly filtered through Celite® and washed with 10.0 mL ethanol in 3 portions directly in the next reaction flask. To this light yellow solution, cyanogen bromide (34.7 mg, 328 µmol, 1.10 equiv) in 1.0 mL ethanol was added under inert gas atmosphere. The mixture was purged again and covered with aluminum foil and stirred for 21 h. The solvent was removed under reduced pressure and the crude residue was dissolved in a mixture of dichloromethane (20.0 mL) and a saturated sodium bicarbonate solution (20.0 mL) and purged with argon. The mixture has turned from purple to light brown. After the mixture was stirred for 20 min, it was transferred to a separation funnel and the aqueous phase was extracted twice with dichloromethane (2 x 20.0 mL). The combined organic phases were dried over anhydrous sodium sulfate, filtered and concentrated under reduced pressure. The crude product was purified via column chromatography with basic aluminum oxide 60 (isocratic, 3% methanol in diethyl ether, when side-product eluted completely the percentage of methanol was increased to 10%). The product was further purified via reversed-phase flash (gradient 10–100% acetonitrile (ACN) in water+0.1% formic acid, product eluates at 40% ACN). Target compound **5h** (43.0 mg, 99.5 µmol, **33% yield**) was isolated as light purple solid (caution: air sensitive). $\boldsymbol{T_m}$ = 130–132 °C. **^{1}H-NMR, COSY** (300 MHz, DMSO-d_6, TMS) δ_H 6.87 (1H, d, *J* = 8.4 Hz, 2-H), 6.43 (1H, d, *J* = 8.4 Hz, 1-H), 6.15 (2H, br, s, 23-H), 4.23 (1H, dd, *J* = 10.3, 3.1 Hz, 10-H), 4.10 – 3.97 (2H, br, m, 24-H), 3.78 (1H, t, *J* = 10.3 Hz, 10-H), 3.33 – 3.02 (8H, m, 8-H, 9-H, 15/16-H, 26-H), 2.88 (3H, s, 15/16-H), 2.76 (3H, s, 28-H), 1.82 (2H, m, 25-H), 1.48 – 1.15 (9H, br, m, 33-35-H). **^{13}C-NMR, HSQC, HMBC** (75 MHz, DMSO-d_6, TMS) δ_C 171.46 (C-12), 154.91 (C-29), 154.45 (C-21), 147.64 (C-6), 136.60 (C-3), 131.50 (C-4), 113.67 (C-2), 109.53 (C-1), 104.08 (C-5), 78.49 (C-32), 66.13 (C-10), 45.61 (C-26), 41.12 (C-24), 36.79 (C-15/16), 35.07 (C-15/16), 34.72 (C-9), 33.70 (C-28), 28.88 (C-25), 28.00 (C-33-35), 23.87 (C-8). **MS (ESI)** found: m/z = 432.2 [M+H$^+$], calculated: m/z = 432.3 [M+H$^+$]. Formation of *tert*-butyl (3-(8-

(dimethylcarbamoyl)-2-methyl-8,9-dihydrochromeno[5,6-*d*]imidazol-1(7*H*)-yl)propyl)(methyl)carbamate as side-product was observed: **^{1}H-NMR, COSY** (300 MHz, DMSO-d_6, TMS) δ_H 7.21 (1H, d, *J* = 8.6 Hz), 6.62 (1H, d, *J* = 8.6 Hz), 4.30 (1H, dd, *J* = 10.1, 3.1 Hz), 4.24 – 4.06 (2H, br, m), 3.84 (1H, t, *J* = 10.1 Hz), 3.32 – 3.17 (5H, m), 3.13 (3H, s), 2.88 (3H, s), 2.79 (3H, s), 2.46 (3H, s), 1.98 – 1.77 (2H, br, m), 1.56 – 1.13 (9H, br, m). **^{13}C-NMR, HSQC, HMBC** (75 MHz, DMSO-d_6, TMS) δ_C 171.29, 154.60, 150.72, 149.71, 136.89, 133.00, 116.92, 111.58, 104.76, 78.62, 66.20, 45.69, 42.41, 36.81, 35.11, 34.45, 33.80, 29.91, 27.98, 23.78, 13.58. **MS (ESI)** found: m/z = 431.2 [M+H$^+$], calculated: m/z = 431.3 [M+H$^+$].

8-((Dimethylamino)methyl)-1-(3-(dimethylamino)propyl)-1,7,8,9-tetrahydrochromeno[5,6-*d*]imidazol-2-amine (5)

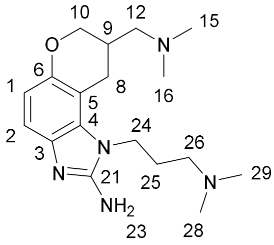

Lithium aluminum hydride (LAH) (9.48 mg, 250 µmol, 3.76 equiv) was added portionwise to a solution of **5h** (28.7 mg, 66.4 µmol, 1.00 equiv) in dry tetrahydrofuran (THF) (3.50 mL) under inert gas atmosphere. The mixture was heated at 60°C for 2 h in a sealed tube, then the same amount of LAH (9.48 mg, 250 µmol, 3.76 equiv) was added and heated for 5 h. Reaction's progress was monitored by LC-MS. After cooling down, the excess hydride was quenched with water (80 µL) followed by 15% sodium hydroxide solution (80 µL). Then additional water was added (240 µL) and a brown solid precipitated. The mixture was filtered over Celite® and the filter pad washed with THF. The filtrate was dried over sodium sulfate, filtered, and concentrated under reduced pressure. The crude product was taken in DMSO and purified *via* reversed phase flash chromatography (isocratic 0% ACN in water+0.1% formic acid; product eluates with DMSO at 0% ACN). The crude product was further purified *via* preparative HPLC (gradient over 10 min 0–5% ACN+0.1% formic acid in water+0.1% formic acid). Target compound **5** (5.23 mg, 15.8 µmol, **24% yield, 99% purity**) was isolated as colorless solid which gets brown upon air contact (keep under inert gas atmosphere). This reaction is recommended to do in the indicated scale. $\boldsymbol{T_m}$ = 107–109 °C. **^{1}H-NMR, COSY** (300 MHz, DMSO-d_6, TMS) δ_H 6.88 (1H, d, *J* = 8.1 Hz, 2-H), 6.45 (1H, d, *J* = 8.1 Hz, 1-H), 6.21 (br, s, 23-H), 4.15 (1H, dd, *J* = 10.6, 2.7 Hz, 10-H), 4.10 – 3.98 (2H, br, m, 24-H), 3.74 (1H, dd, *J* = 10.5, 7.6 Hz, 10-H), 3.17 (1H, dd, *J* = 16.0, 5.5 Hz, 8-H), 2.78 (1H, dd, *J* = 16.0, 7.6 Hz, 8-H), 2.43 – 2.27 (4H, m, 26-H, 12-H), 2.26 (6H, s, 28-29-H), 2.23 (7H, s, 15-16-H, 9-H), 1.94 – 1.74 (2H, br, m, 25-H). **^{13}C-NMR, HSQC, HMBC** (75 MHz, DMSO-d_6, TMS) δ_C 154.22 (C-21), 148.36 (C-6), 134.54 (C-3), 131.22 (C-4), 112.95 (C-2), 109.99 (C-1), 104.53 (C-5), 67.61 (C-10), 60.61 (C-12), 54.76 (C-26), 45.37 (C-15-16), 44.30 (C-28-29), 40.77 (C-24), 29.57 (C-9), 27.61 (C-25), 24.58 (C-8). **MS (ESI)** found: m/z = 332.2 [M+H$^+$], calculated: m/z = 332.2 [M+H$^+$].

Analytics compound 5

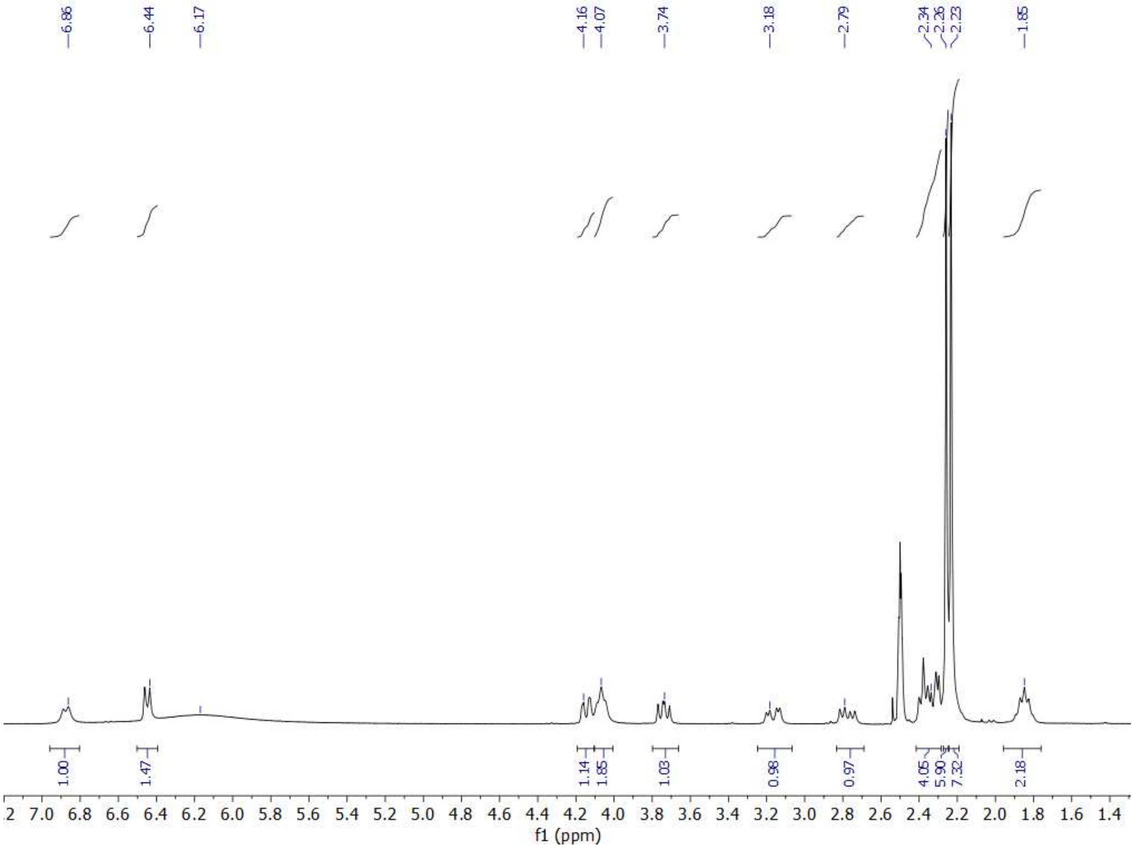

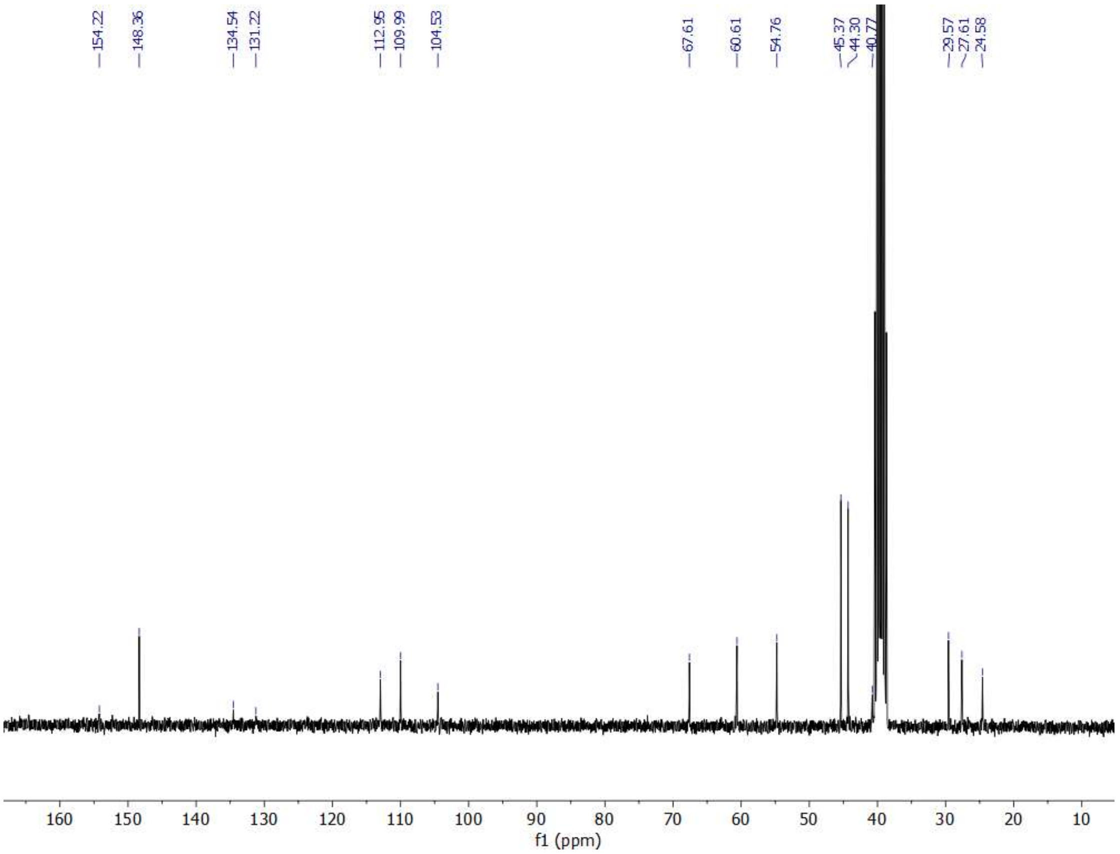

Figure S 7: NMR-spectra of final compound **5**: ^{1}H-NMR spectrum measured in DMSO-d6 (top), ^{13}C-NMR spectrum measured in DMSO-d6 (bottom).

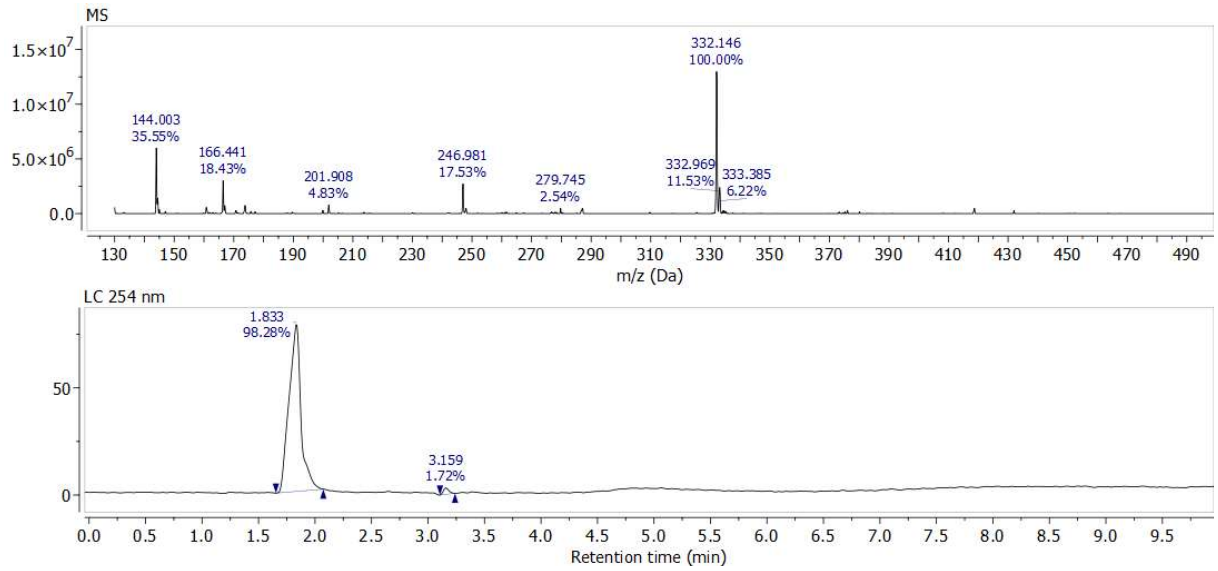

Figure S 8: LC-ESI-MS (measured with Zorbax column, gradient 10–90% ACN in water + 0.1% formic acid over 10 min) traces of compound **5**, top: mass spectrum, bottom: LC chromatogram.

4. Analytical data of virtual screening hits

General experimental methods

High-performance liquid chromatography/electron spray ionization mass spectrometry (**HPLC/ESI-MS**) was used to determine the identity as well as the purity of compounds tested in the assay. An Agilent 1100 series HPLC system and either an Agilent Zorbax SB-Aq (4.6 mm x 150 mm, 5 µm) or Poroshell 120 EC-C18 (2.10 mm x 150 mm, 4 µM) column coupled to an Agilent 1100 series LC/MSD Trap with ESI, was used. The measurements were conducted with a gradient of acetonitrile and water (+0.1% formic acid) with 10%–90% acetonitrile over 10 min with a flow rate of 0.7 mL/min. Signals were detected at 254 nm with quantification by area under curve (AUC) and masses were determined in positive ionization mode (ESI) unless otherwise stated. Nuclear magnetic resonance (**NMR**) spectra were recorded on a Bruker Avance Neo 400 MHz NMR spectrometer. The chemical shift was abbreviated to δ and has the unit ppm. Tetramethyl silane (TMS) (δ = 0 ppm) was used as the reference substance. The chemical shifts were referenced to the solvent peaks in ^{1}H: δ = 3.31 (Methanol-d_4), 2.50 (DMSO-*d*6), 4.79 (Deuterium oxide) ppm and in ^{13}C: δ = 49.00 (Methanol-d_4), 39.52 (DMSO-d6) ppm purchased from Deutero GmbH. TMS was used as reference substance for ^{13}C spectra measured in deuterium oxide. In addition, the following abbreviations for the multiplicities of the peaks were defined: s (singlet), d (doublet), dd (doublet of doublet), t (triplet), dt (doublet of triplet), q (quartet), qd (quartet of doublet) and m (multiplet). In the evaluation of the 1H-NMR spectrum, the coupling constant *J* was given in Hz and denoted together with the number of signalling hydrogen atoms. The MestReNova 12.0.4-22023 NMR spectrum processing program from *Mestrelab Research* was used to evaluate the NMR spectra and to determine the purity of the compounds through LC-MS.

For compounds **8** and **8b**, additional purification by **preparative HPLC** was performed with an Agilent 1290 II Infinity Preparative LC System using a MZ-Aqua Perfect C18 20 x 250 mm, 7 µm preparative LC column and acetonitrile/water +0.1% formic acid as the mobile phase. The impurity of compound **8b** was isolated and resulted in compound **8c**.

Spectra of VS hits

Analytical data of the purchased VS hits and compound 4 including LC-MS analytics (top: mass spectrum, bottom: LC chromatogram at 254 nm, column used is indicated) and NMR spectroscopy (^{1}H-NMR and ^{13}C-NMR, if possible). Due to low amounts of purchased compounds, determination of ^{1}H- and ^{13}C-NMR spectra was not always feasible. In these cases, ^{1}H-NMR spectra of the vendor are depicted, if provided. Therefore, ^{13}C-NMR spectra are not available for compounds **9**, **12**, **15**, **17**, **20**, **23** and **24**. For compound **9**, also no ^{1}H-NMR spectrum is available.

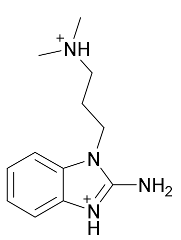

4

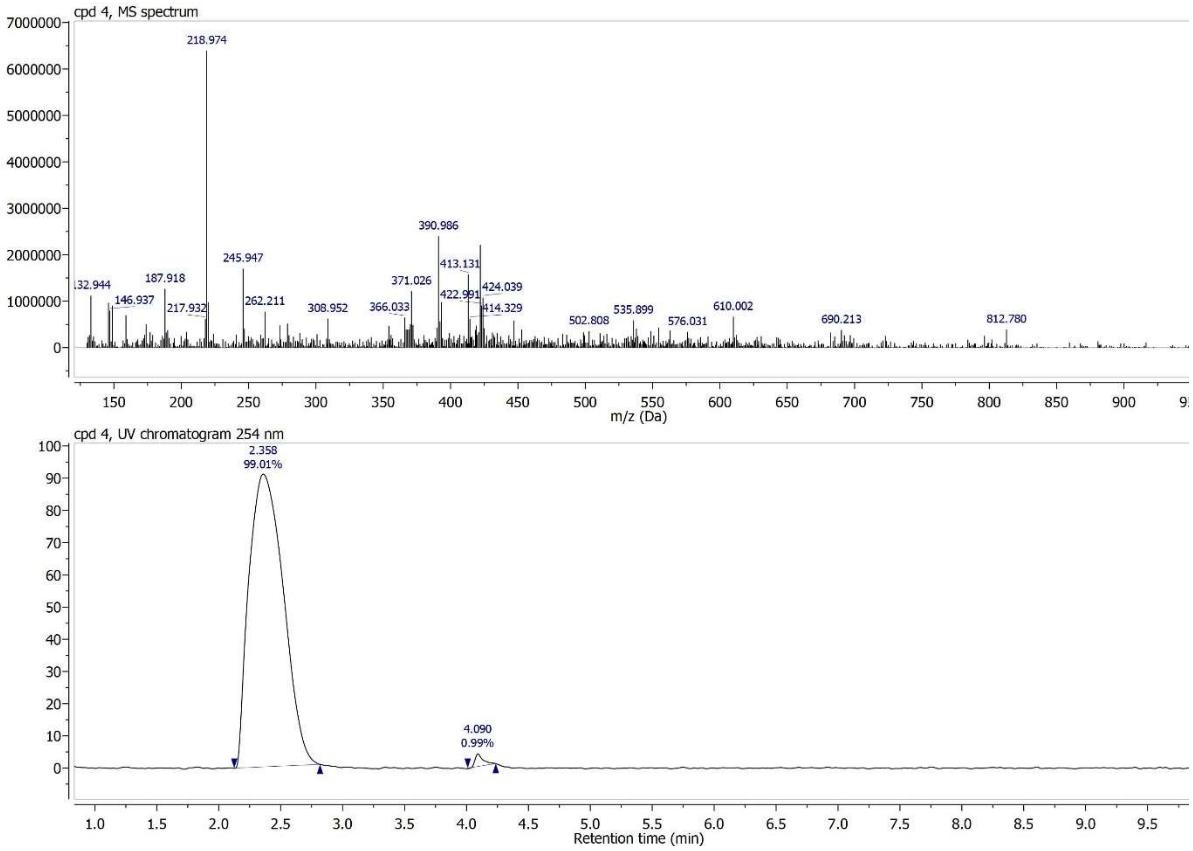

LC-ESI-MS (measured with Zorbax column, gradient 10–90% ACN in water + 0.1% formic acid over 10 min), top: mass spectrum, bottom: LC chromatogram at 254 nm. Calculated: m/z = 219.3 [M + H$^+$], found: m/z = 219.0 [M + H$^+$], purity 99.0%.

4

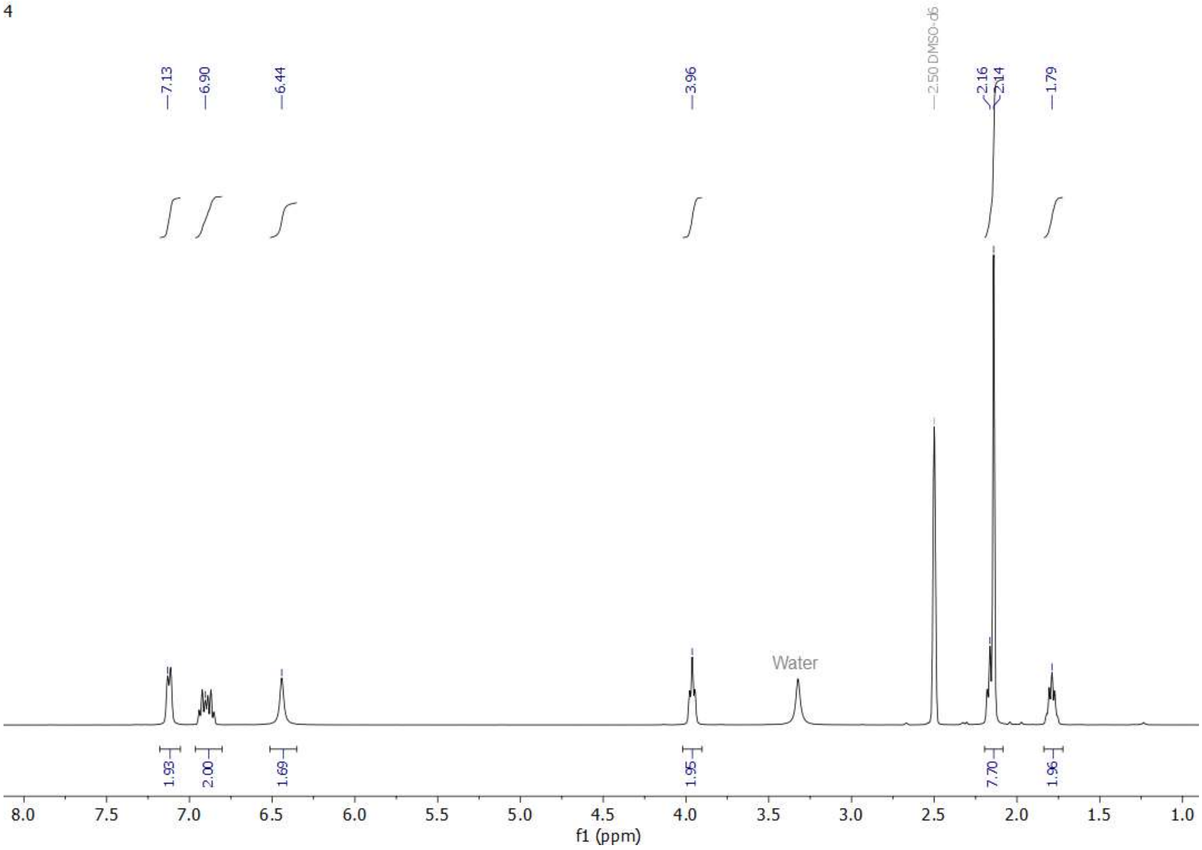

^{1}H NMR (400 MHz, DMSO-d_6, TMS) δ_H 7.12 (2H, d, J = 7.5 Hz), 6.98 – 6.77 (2H, m), 6.44 (2H, s), 3.96 (2H, t, J = 6.8 Hz), 2.20 – 2.11 (8H, m), 1.79 (2H, t, J = 6.8 Hz).

4

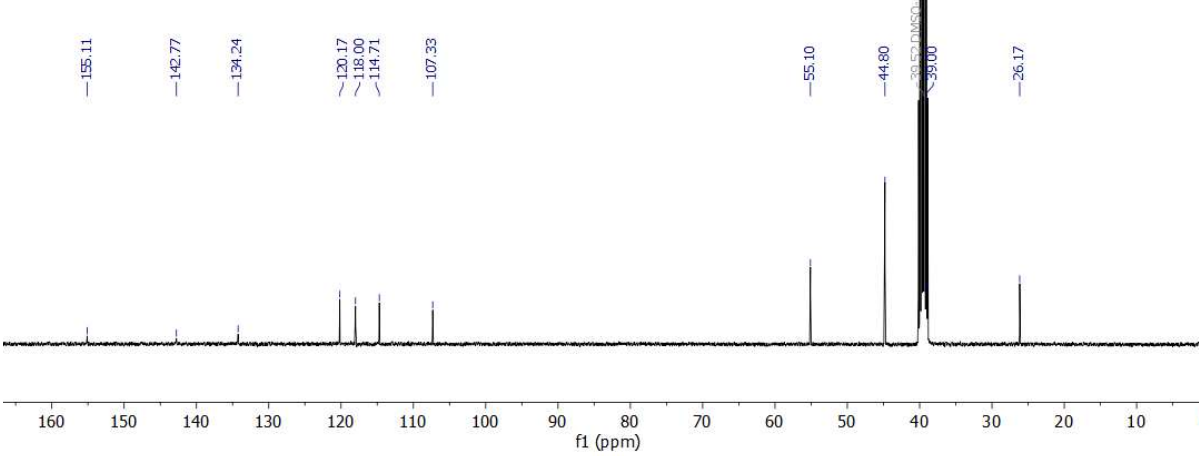

^{13}C NMR (101 MHz, DMSO-d_6, TMS) δ_C 155.11, 142.77, 134.24, 120.17, 118.00, 114.71, 107.33, 55.10, 44.80, 39.00, 26.17.

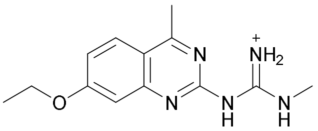

8

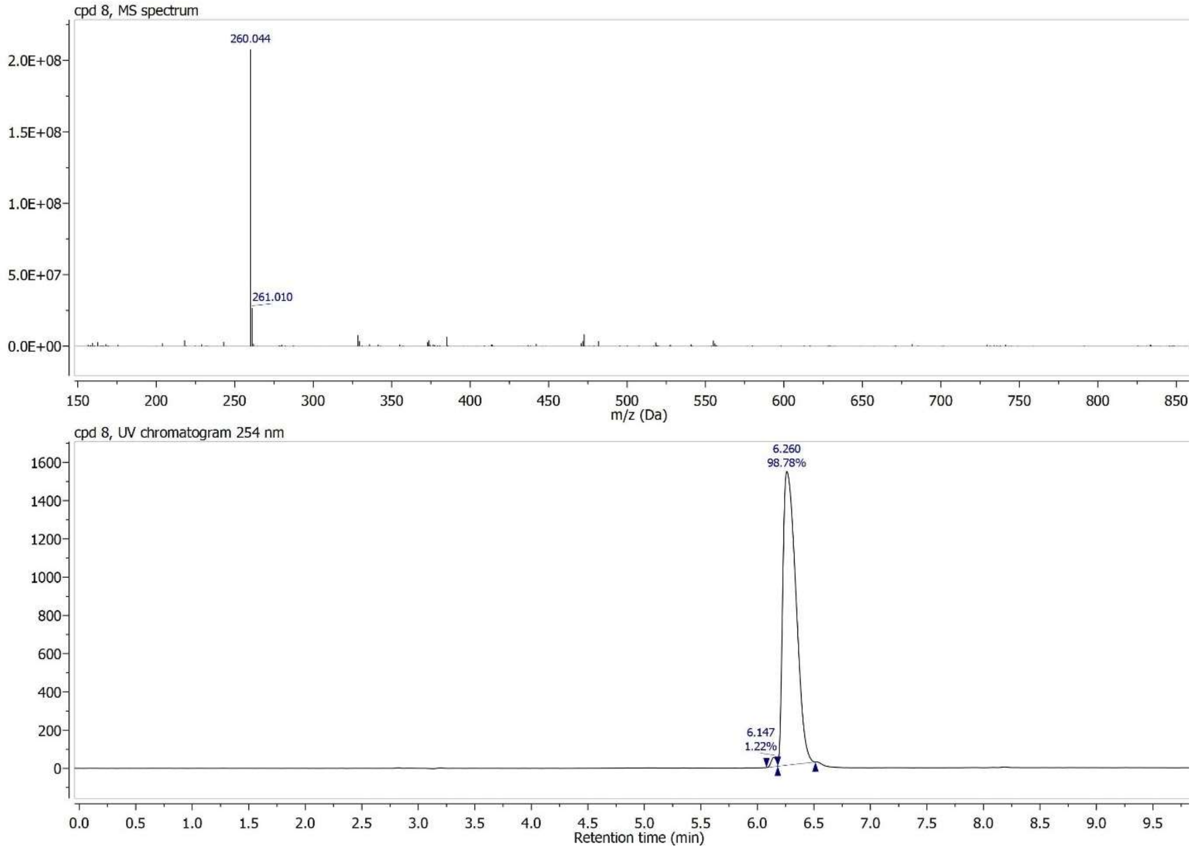

LC-ESI-MS (measured with Zorbax column, gradient 10–90% ACN in water + 0.1% formic acid over 10 min), top: mass spectrum, bottom: LC chromatogram at 254 nm. Calculated: m/z = 260.2 [M + H$^+$], found: m/z = 260.0 [M + H$^+$], purity 98.8%.

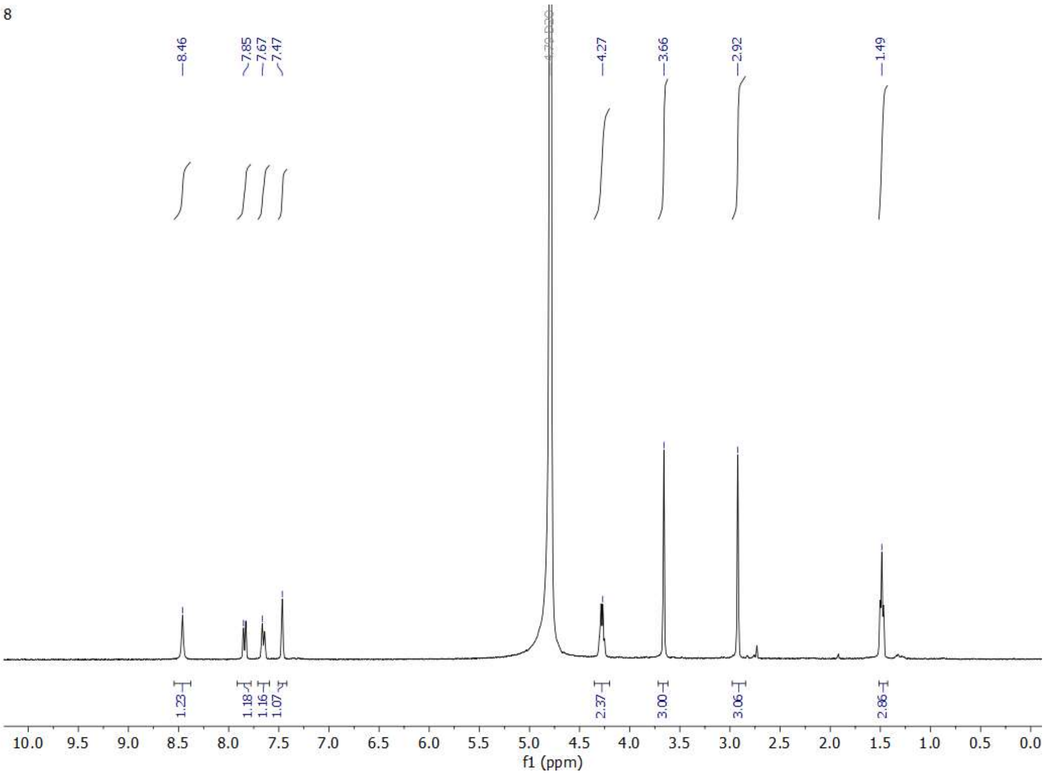

^{1}H NMR (400 MHz, Deuterium Oxide, TMS) δ_H 8.46 (1H, s), 7.93 – 7.78 (1H, m), 7.72 – 7.58 (1H, m), 7.52 – 7.41 (1H, m), 4.28 (2H, q, *J* = 7.5, 7.1 Hz), 3.66 (3H, s), 2.92 (3H, s), 1.49 (3H, t, *J* = 6.8, 3.6 Hz).

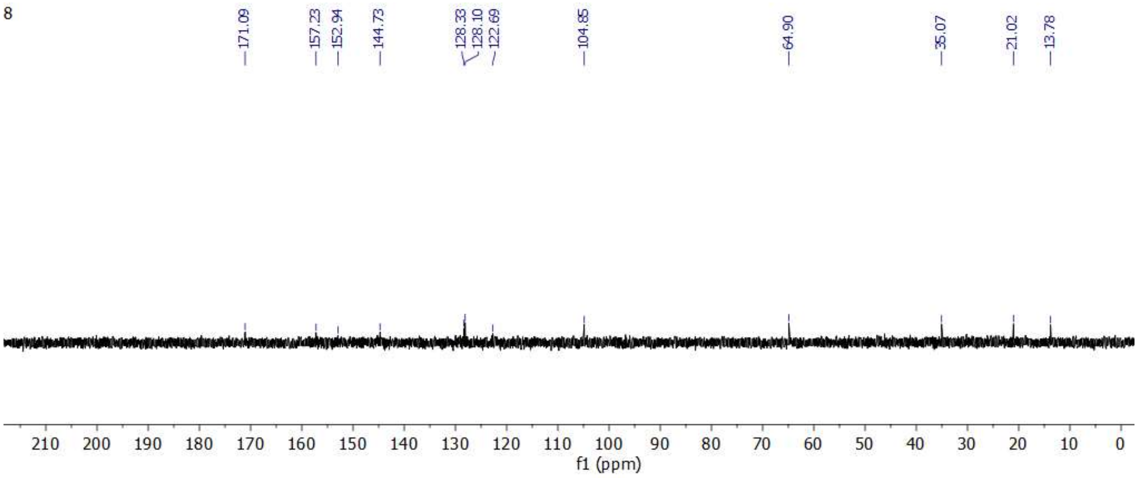

^{13}C NMR (101 MHz, Deuterium Oxide, TMS) δ_C 171.09, 157.23, 152.94, 144.73, 128.33, 128.10, 122.69, 104.85, 64.90, 35.07, 21.02, 13.78.

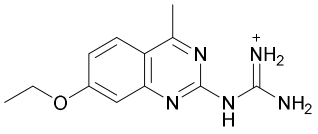

8a

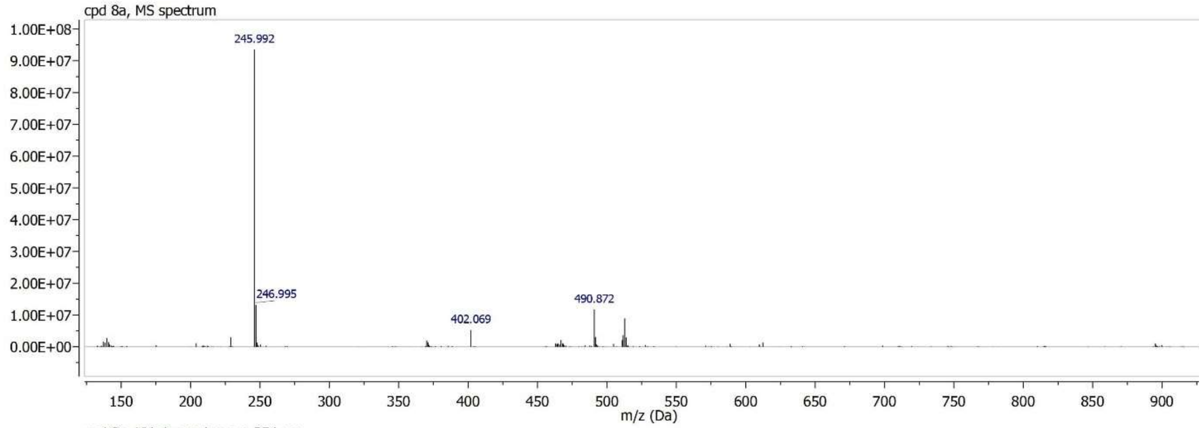

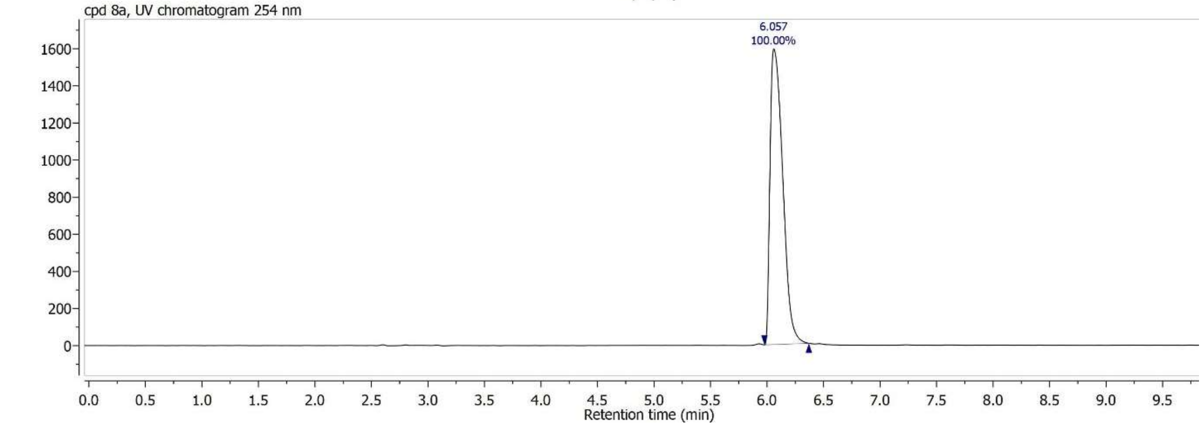

LC-ESI-MS (measured with Zorbax column, gradient 10–90% ACN in water + 0.1% formic acid over 10 min), top: mass spectrum, bottom: LC chromatogram at 254 nm. Calculated: m/z = 246.1 [M + H$^+$], found: m/z = 246.0 [M + H$^+$], purity 100.0%.

8a

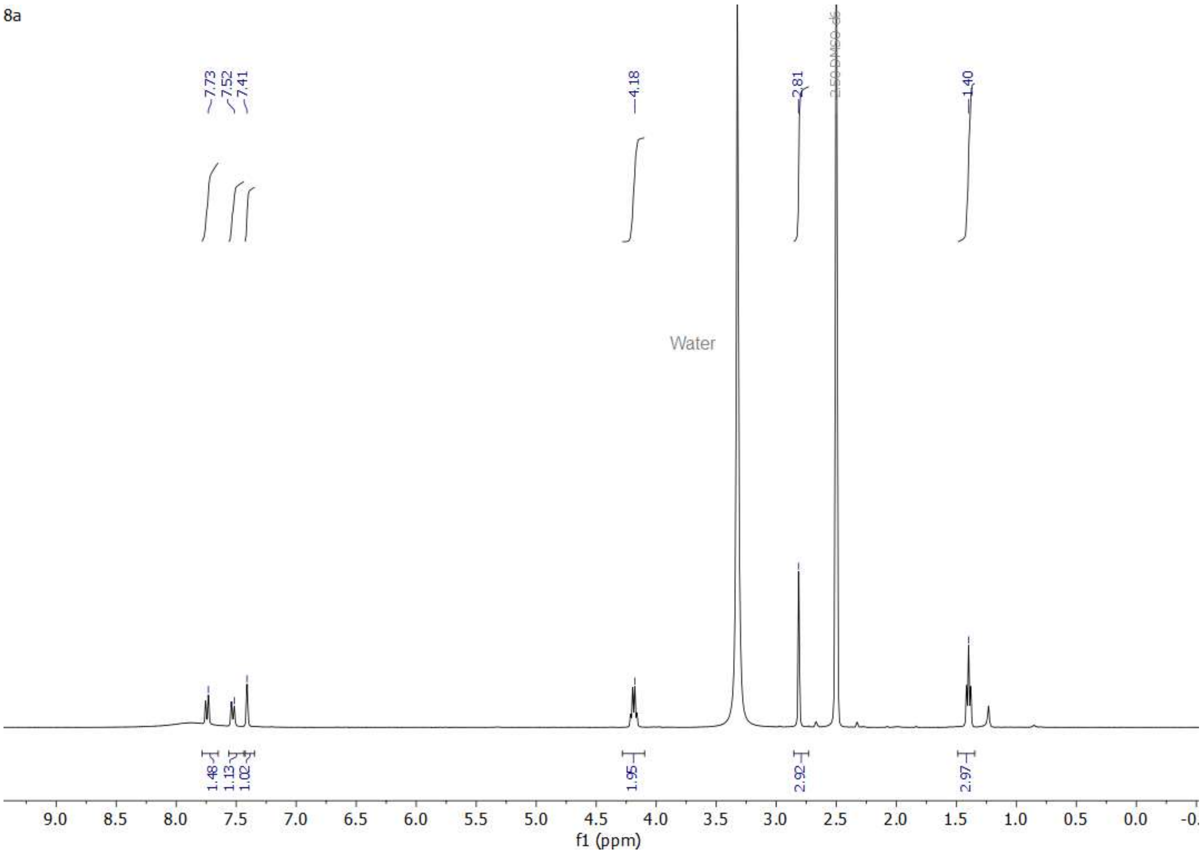

^{1}H NMR (400 MHz, DMSO-d_6, TMS) δ_H 7.74 (1H, dd, *J* = 9.1, 2.0 Hz), 7.53 (1H, dt, *J* = 9.1, 3.8, 2.4 Hz), 7.41 (1H, s), 4.19 (2H, q, *J* = 6.4 Hz), 2.81 (3H, s), 1.40 (3H, t, *J* = 6.1 Hz).

8a

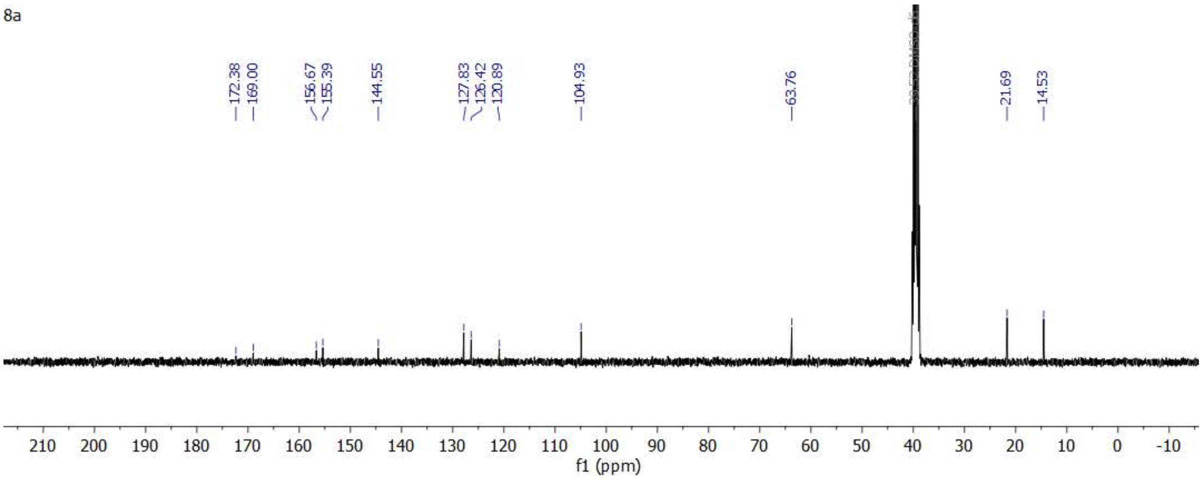

^{13}C NMR (101 MHz, DMSO-d_6, TMS) δ_C 172.38, 169.00, 156.67, 155.39, 144.55, 127.83, 126.42, 120.89, 104.93, 63.76, 21.69, 14.53.

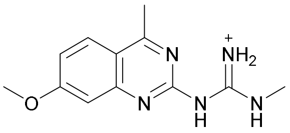

8b

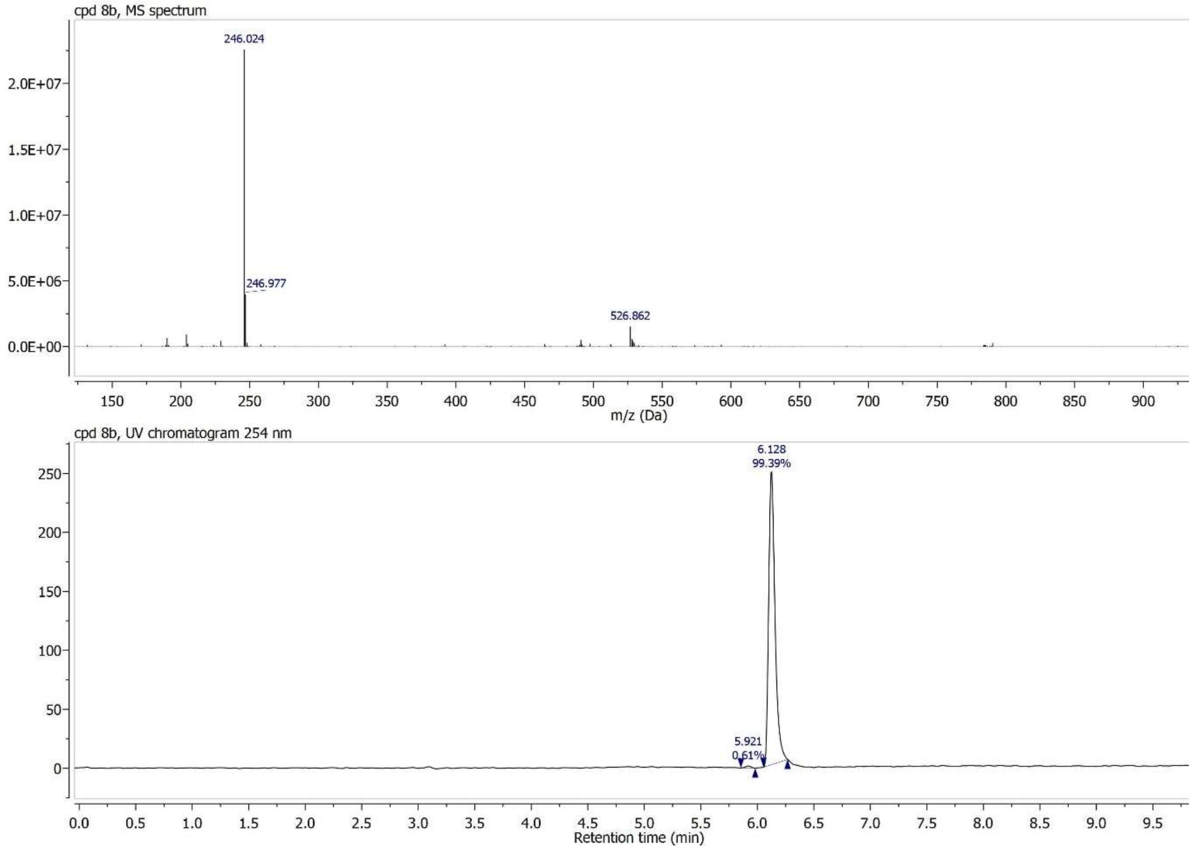

LC-ESI-MS (measured with Zorbax column, gradient 10–90% ACN in water + 0.1% formic acid over 10 min), top: mass spectrum, bottom: LC chromatogram at 254 nm. Calculated: m/z = 246.1 [M + H$^+$], found: m/z= 246.0 [M + H$^+$], purity 99.4%.

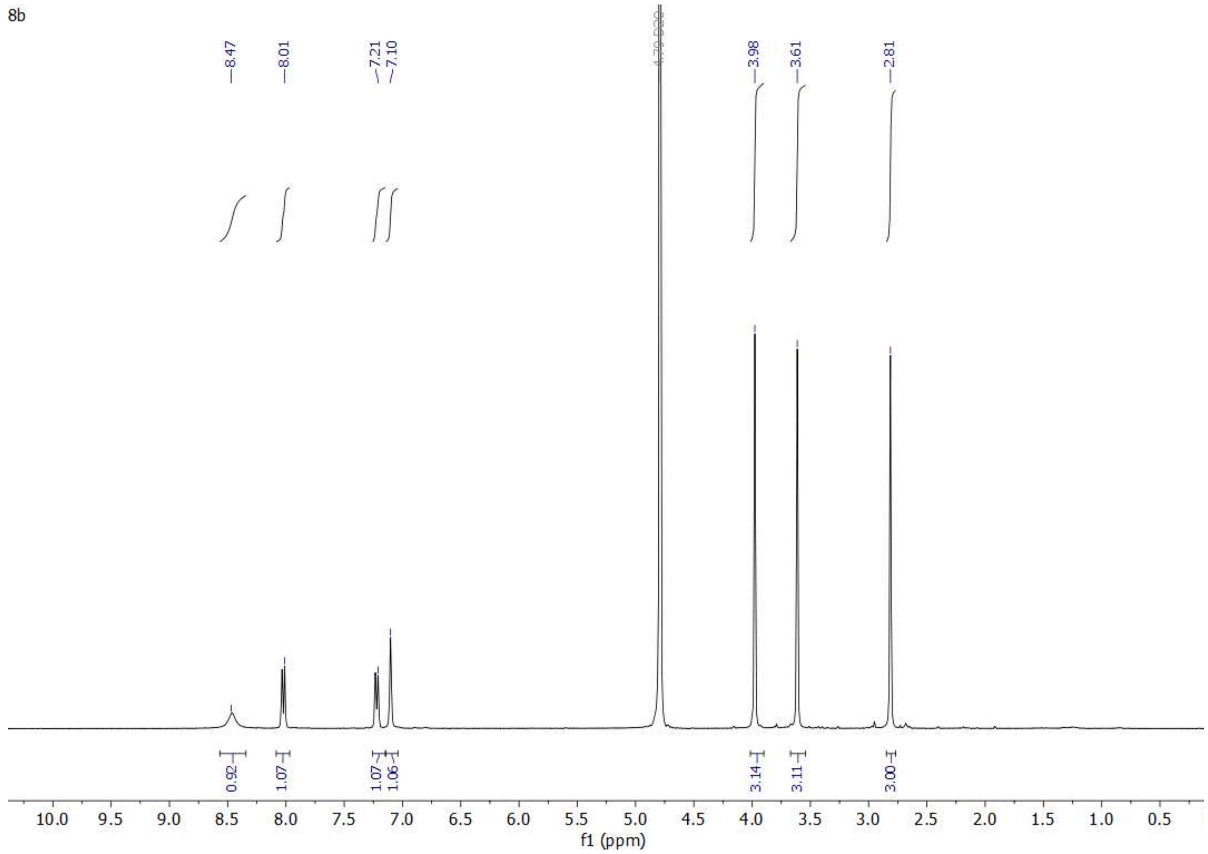

^{1}H NMR (400 MHz, Deuterium Oxide, TMS) δ_H 8.46 (1H, s), 8.02 (1H, d, *J* = 9.1 Hz), 7.22 (1H, d, *J* = 9.1 Hz), 7.10 (1H, s), 3.98 (3H, s), 3.61 (3H, s), 2.81 (3H, s).

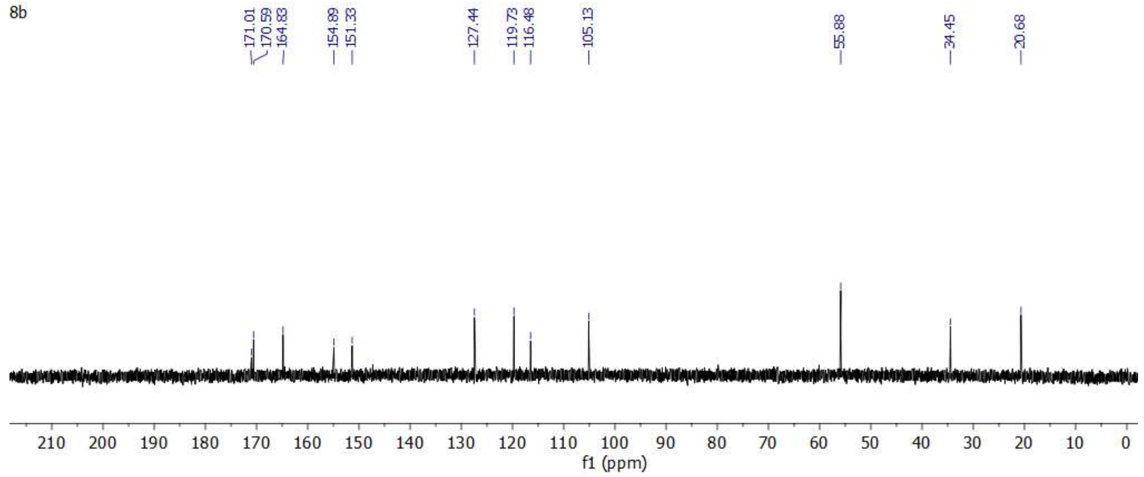

^{13}C NMR (101 MHz, Deuterium Oxide, TMS) δ_C 171.01, 170.59, 164.83, 154.89, 151.33, 127.44, 119.73, 116.48, 105.13, 55.88, 34.45, 20.68.

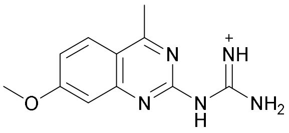

8c

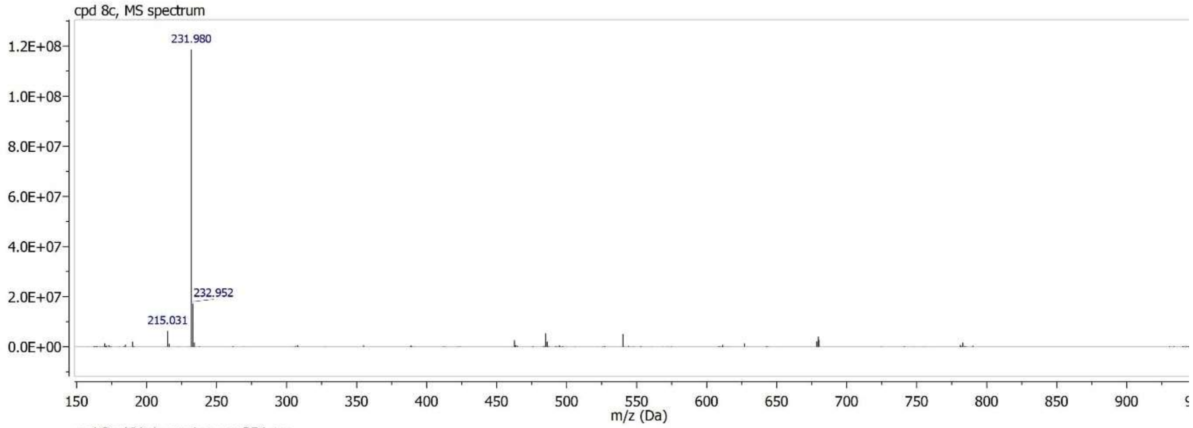

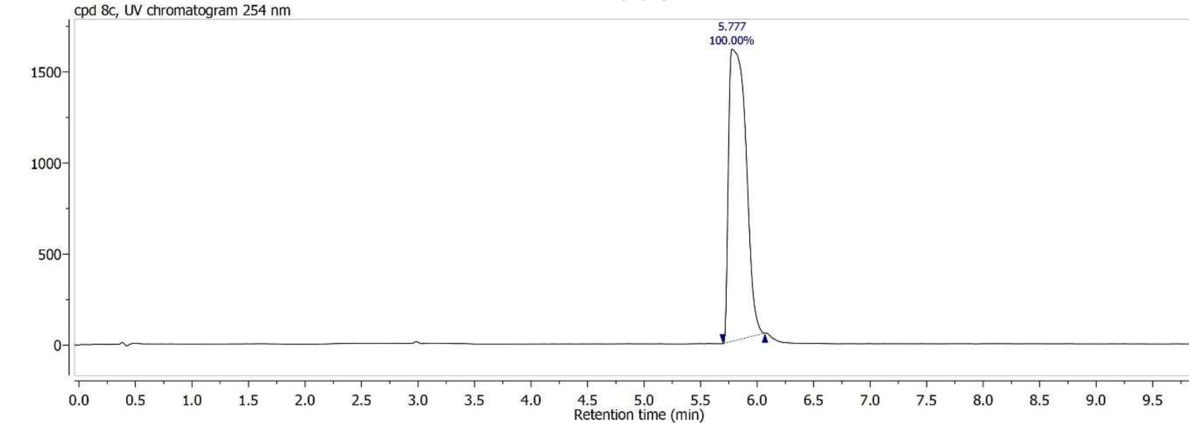

LC-ESI-MS (measured with Zorbax column, gradient 10–90% ACN in water + 0.1% formic acid over 10 min), top: mass spectrum, bottom: LC chromatogram at 254 nm. Calculated: m/z = 232.1 [M + H$^+$], found: m/z = 232.0 [M + H$^+$], purity 100.0%.

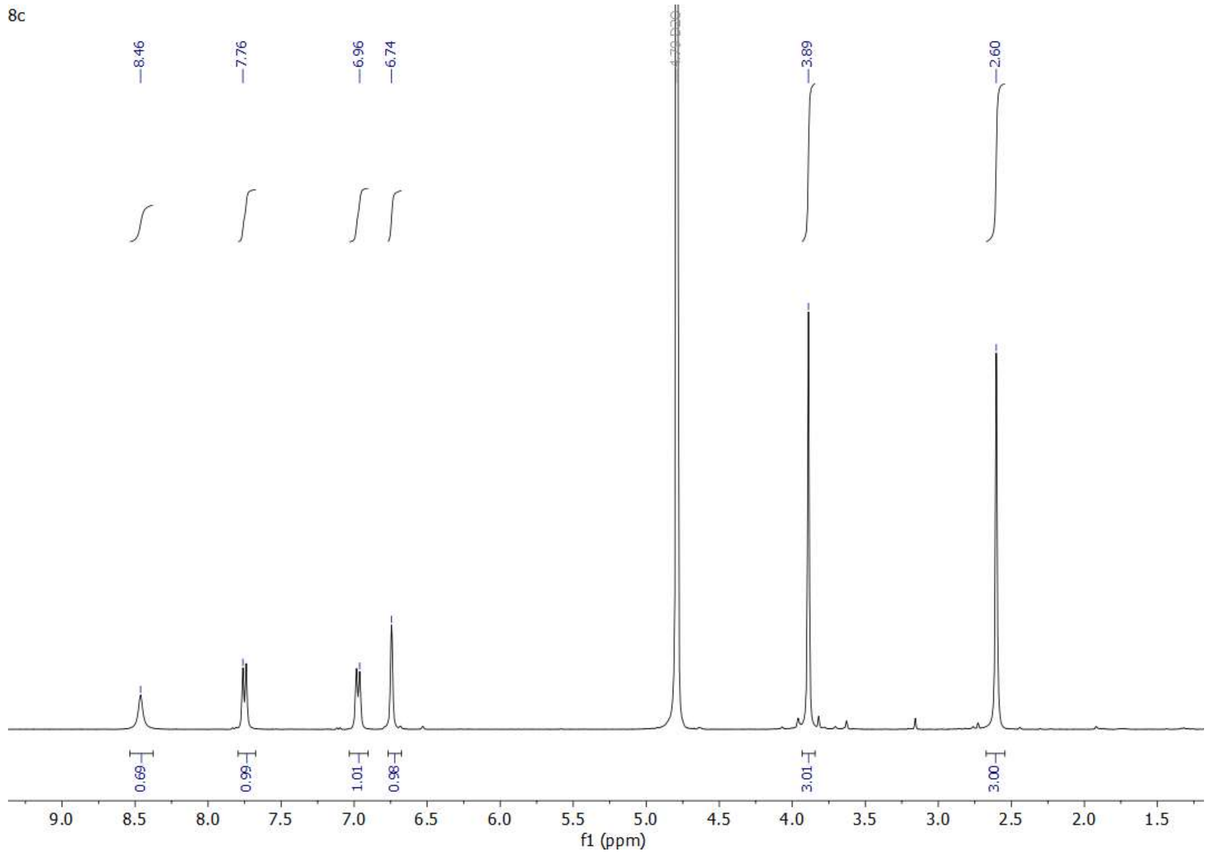

^{1}H NMR (400 MHz, Deuterium Oxide, TMS) δ_H 8.46 (1H, s), 7.75 (1H, d, *J* = 9.1 Hz), 6.97 (1H, d, *J* = 9.0 Hz), 6.74 (1H, s), 3.89 (3H, s), 2.60 (3H, s).

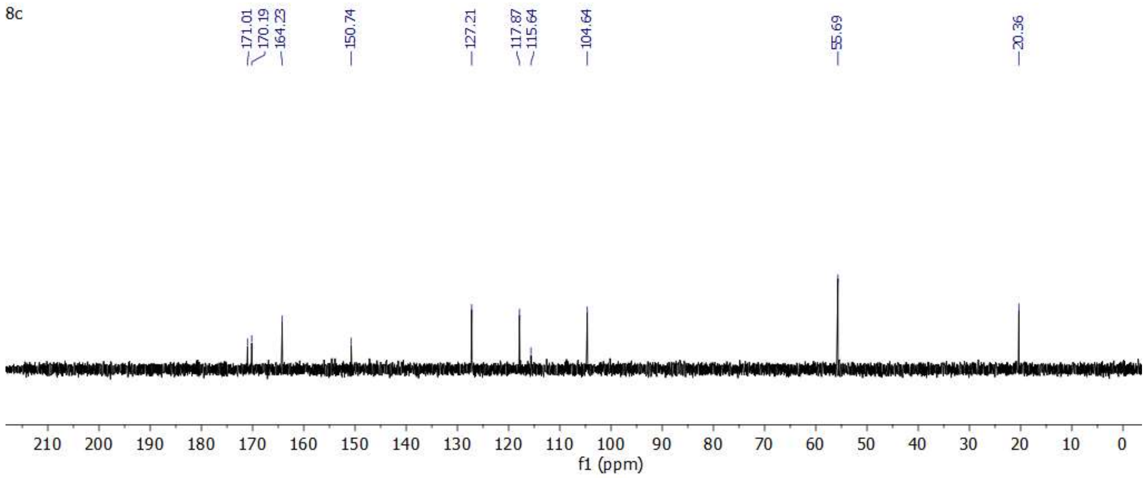

^{13}C NMR (101 MHz, Deuterium Oxide, TMS) δ_C 171.01, 170.19, 164.23, 150.74, 127.21, 117.87, 115.64, 104.64, 55.69, 20.36.

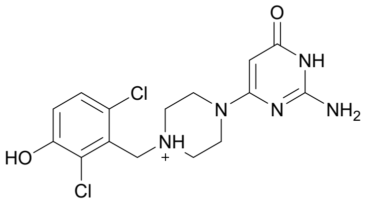

9

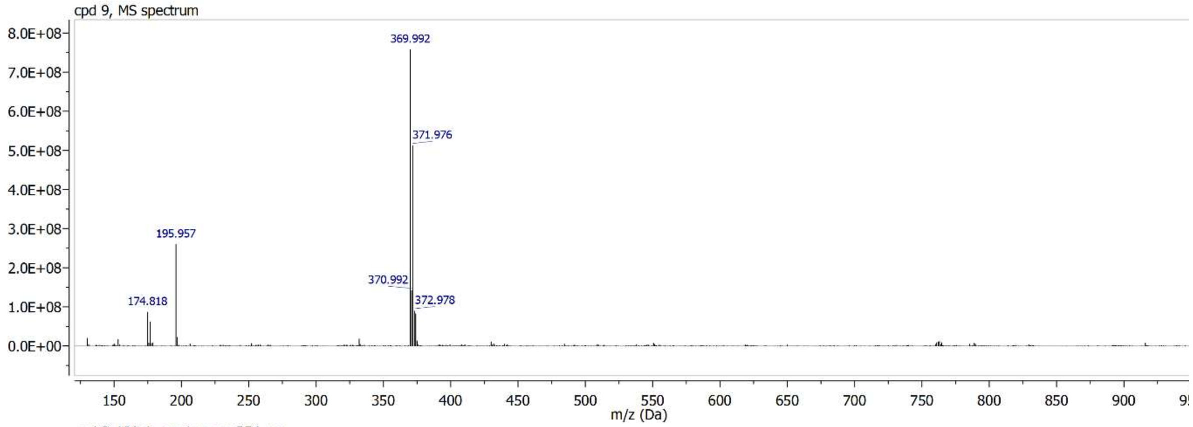

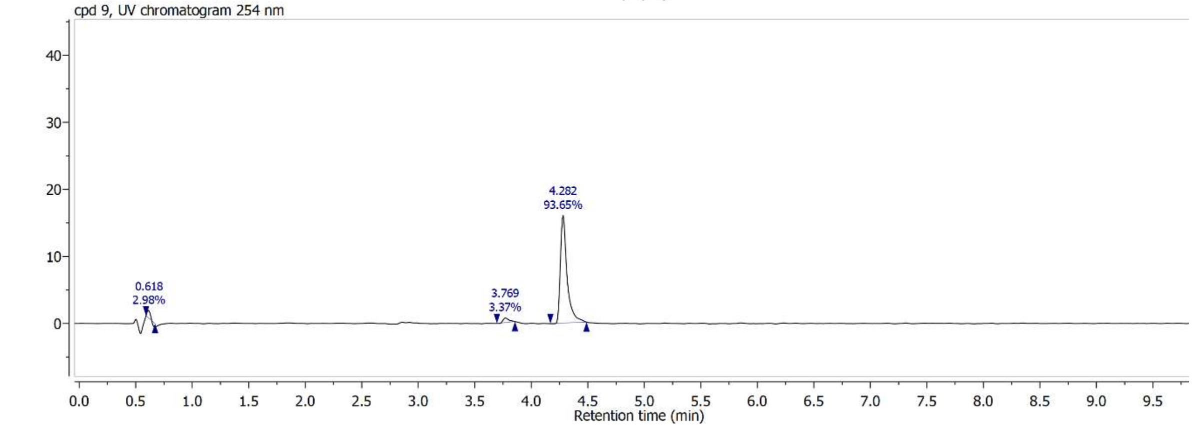

LC-ESI-MS (measured with Poroshell column, gradient 10–90% ACN in water + 0.1% formic acid over 10 min), top: mass spectrum, bottom: LC chromatogram at 254 nm. Calculated: m/z = 370.1 [M + H^+], found: m/z = 370.0 [M + H^+], purity 93.7%.

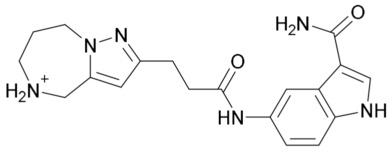

10

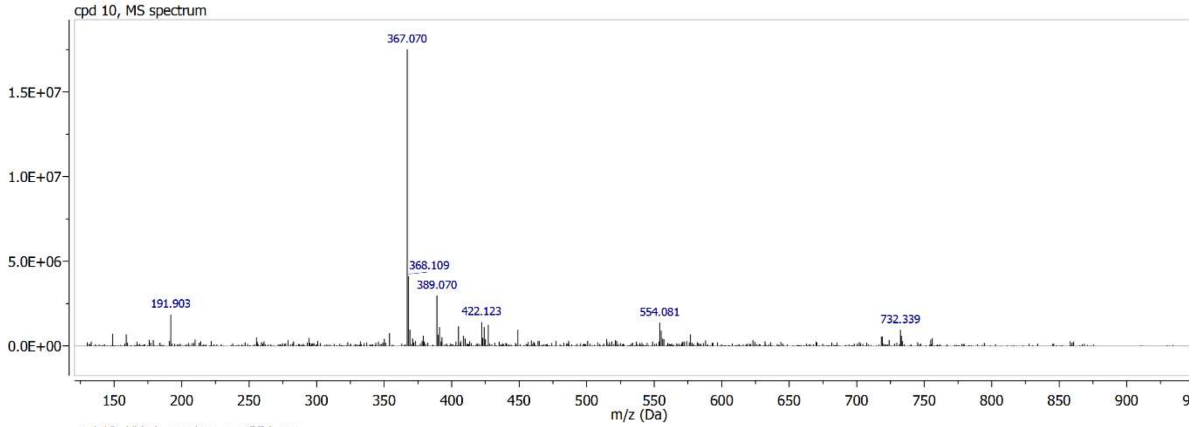

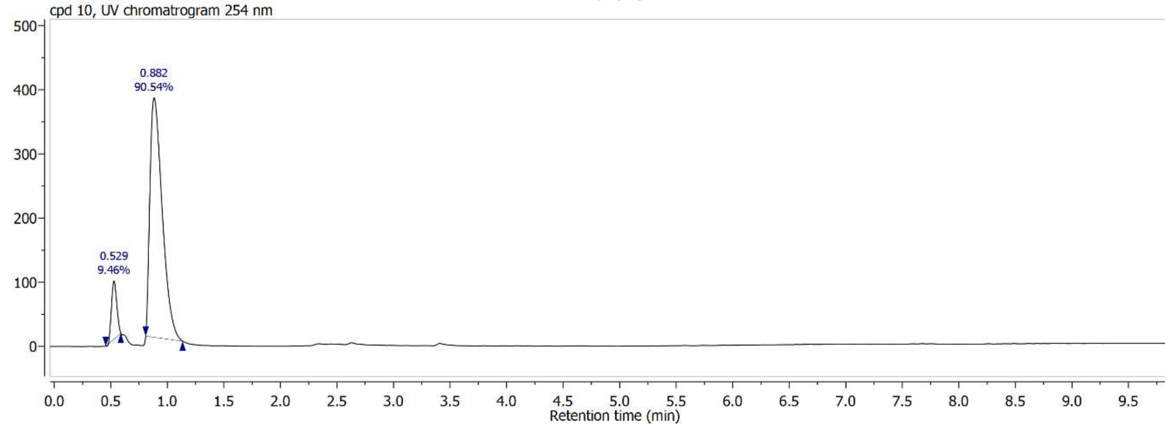

LC-ESI-MS (measured with Poroshell column, gradient 10–90% ACN in water + 0.1% formic acid over 10 min), top: mass spectrum, bottom: LC chromatogram at 254 nm. Calculated: m/z = 367.2 [M + H^+], found: m/z = 367.1 [M + H^+], purity 90.5%.

10

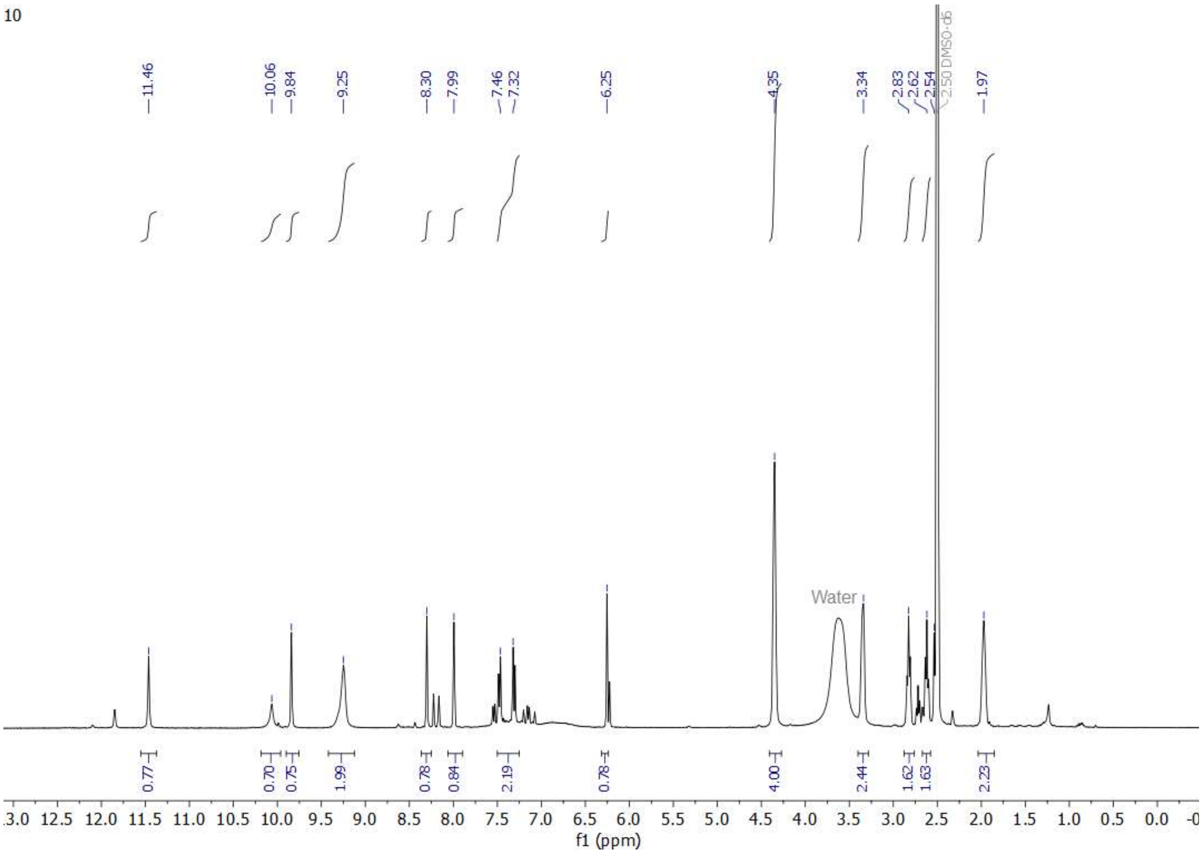

^{1}H NMR (400 MHz, DMSO-d_6, TMS) δ_H 11.46 (1H, s), 10.06 (1H, s), 9.84 (1H, s), 9.25 (2H, s), 8.30 (1H, s), 8.00 (1H, d, J = 2.5 Hz), 7.48 – 7.30 (2H, m), 7.31 (1H, d, J = 8.9 Hz), 6.25 (1H, s), 4.44 – 4.23 (4H, m), 3.43 – 3.25 (2H, m), 2.83 (2H, t, J = 7.7 Hz), 2.62 (2H, t, J = 7.8 Hz), 2.08 – 1.86 (2H, m).

10

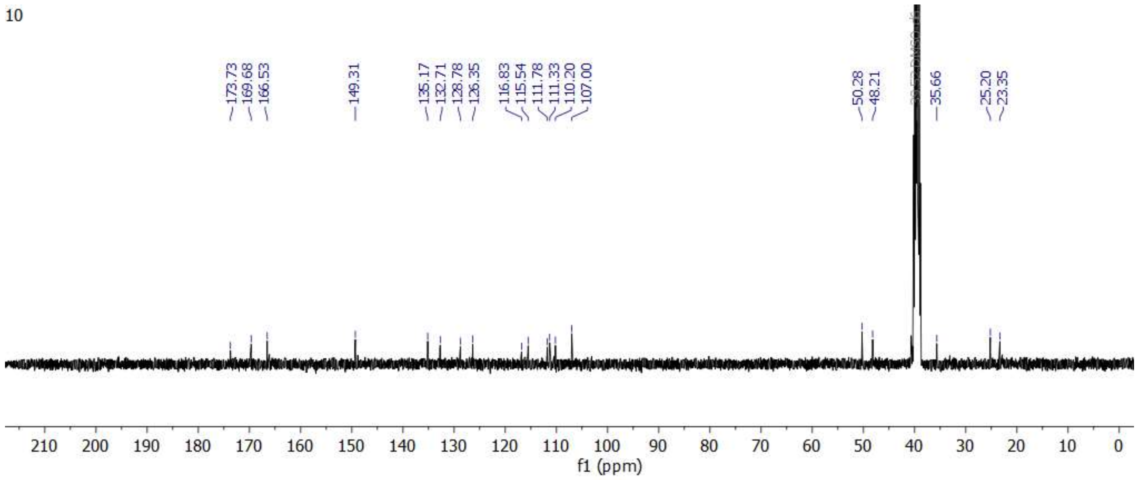

^{13}C NMR (101 MHz, DMSO-d_6, TMS) δ_C 173.73, 169.68, 166.53, 149.31, 135.17, 132.71, 128.78, 126.35, 116.83, 115.54, 111.78, 111.33, 110.20, 107.00, 50.28, 48.21, 35.66, 25.20, 23.35.

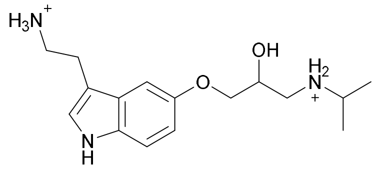

11

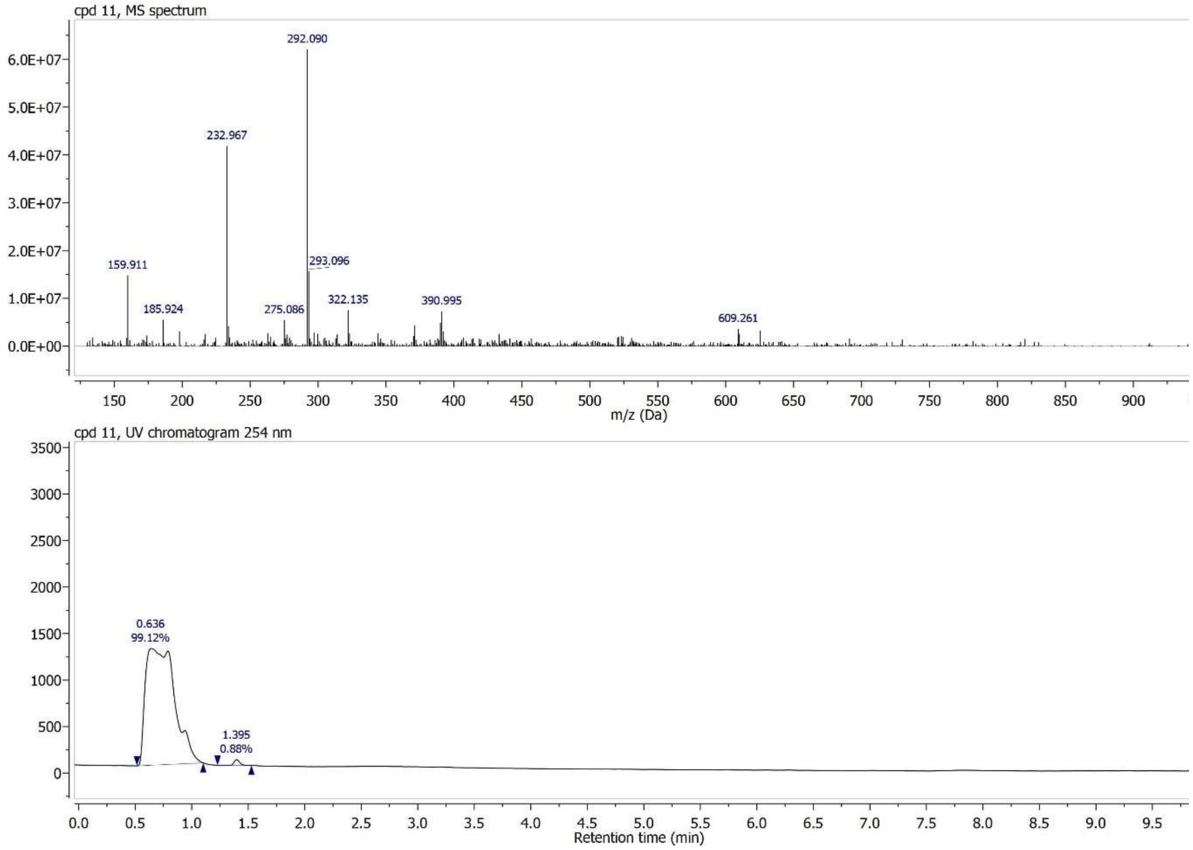

LC-ESI-MS (measured with Poroshell column, gradient 10–90% ACN in water + 0.1% formic acid over 10 min), top: mass spectrum, bottom: LC chromatogram at 254 nm. Calculated: m/z = 292.2 [M + H$^+$], found: m/z = 292.1 [M + H$^+$], purity 99.1%.

11

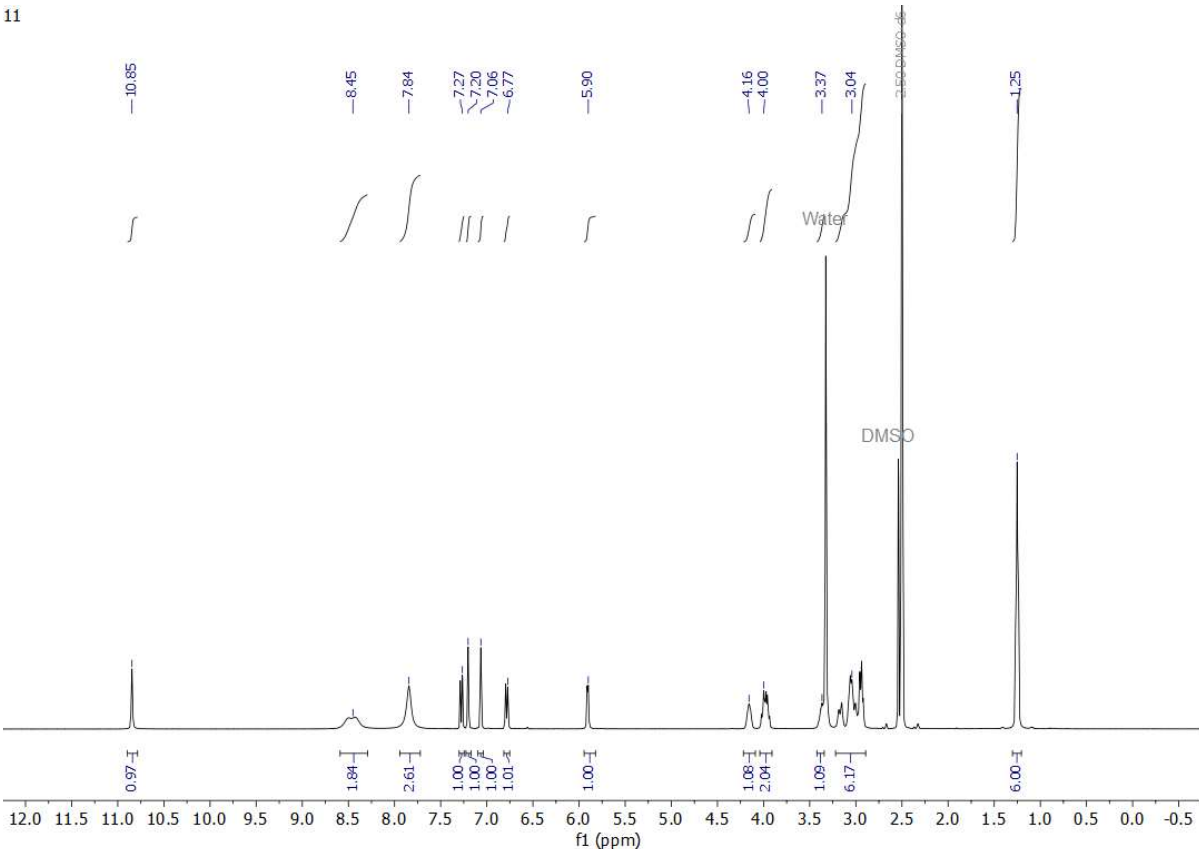

^{1}H NMR (400 MHz, DMSO-d_6, TMS) δ_H 10.85 (1H, s), 8.66 – 8.28 (2H, m), 7.84 (3H, s), 7.28 (1H, dd, J = 8.8, 1.9 Hz), 7.20 (1H, d, J = 2.8 Hz), 7.06 (1H, d, J = 2.6 Hz), 6.79 (1H, dd, J = 8.7, 2.2 Hz), 5.91 (1H, d, J = 5.0 Hz), 4.15 (1H, dd, J = 9.9, 5.4 Hz), 3.98 (2H, qd, J = 9.8, 4.9 Hz), 3.42 – 3.35 (1H, m), 3.23 – 2.86 (6H, m), 1.25 (6H, t, J = 5.9 Hz).

11

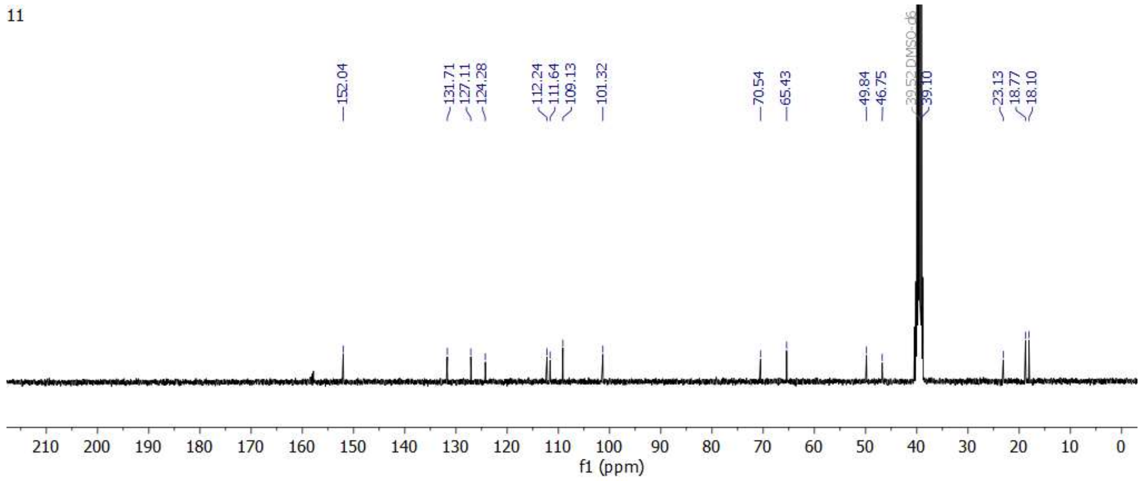

^{13}C NMR (101 MHz, DMSO-d_6, TMS) δ_C 152.04, 131.71, 127.11, 124.28, 112.24, 111.64, 109.13, 101.32, 70.54, 65.43, 49.84, 46.75, 39.10, 23.13, 18.77, 18.10.

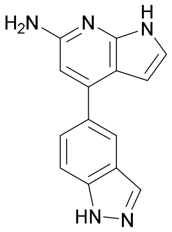

12

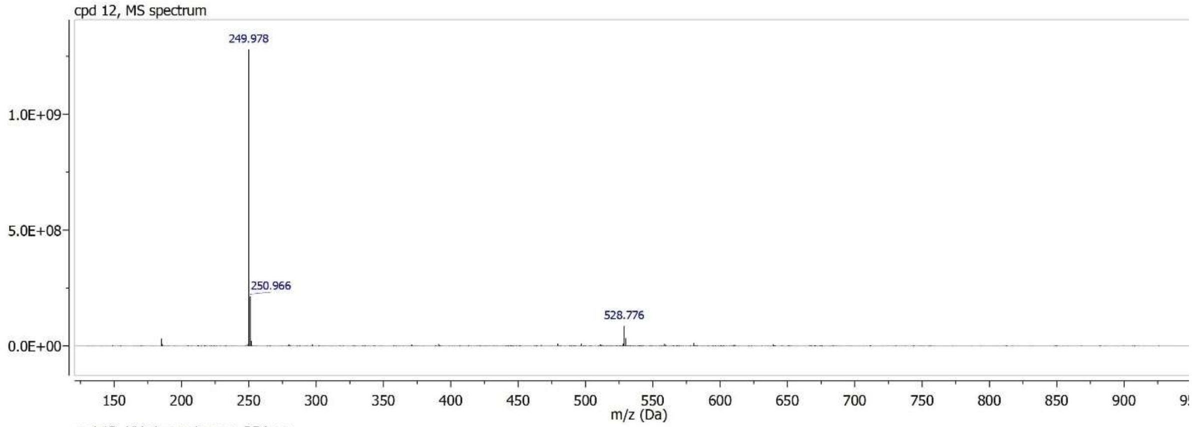

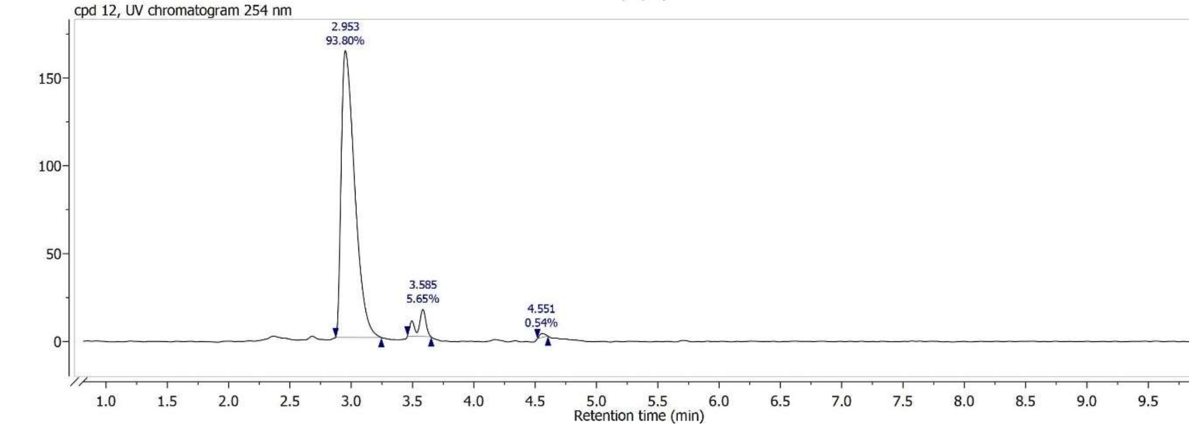

LC-ESI-MS (measured with Poroshell column, gradient 10–90% ACN in water + 0.1% formic acid over 10 min), top: mass spectrum, bottom: LC chromatogram at 254 nm. Calculated: m/z = 250.1[M + H^+], found: m/z = 250.0[M + H^+], purity 93.8%.

12

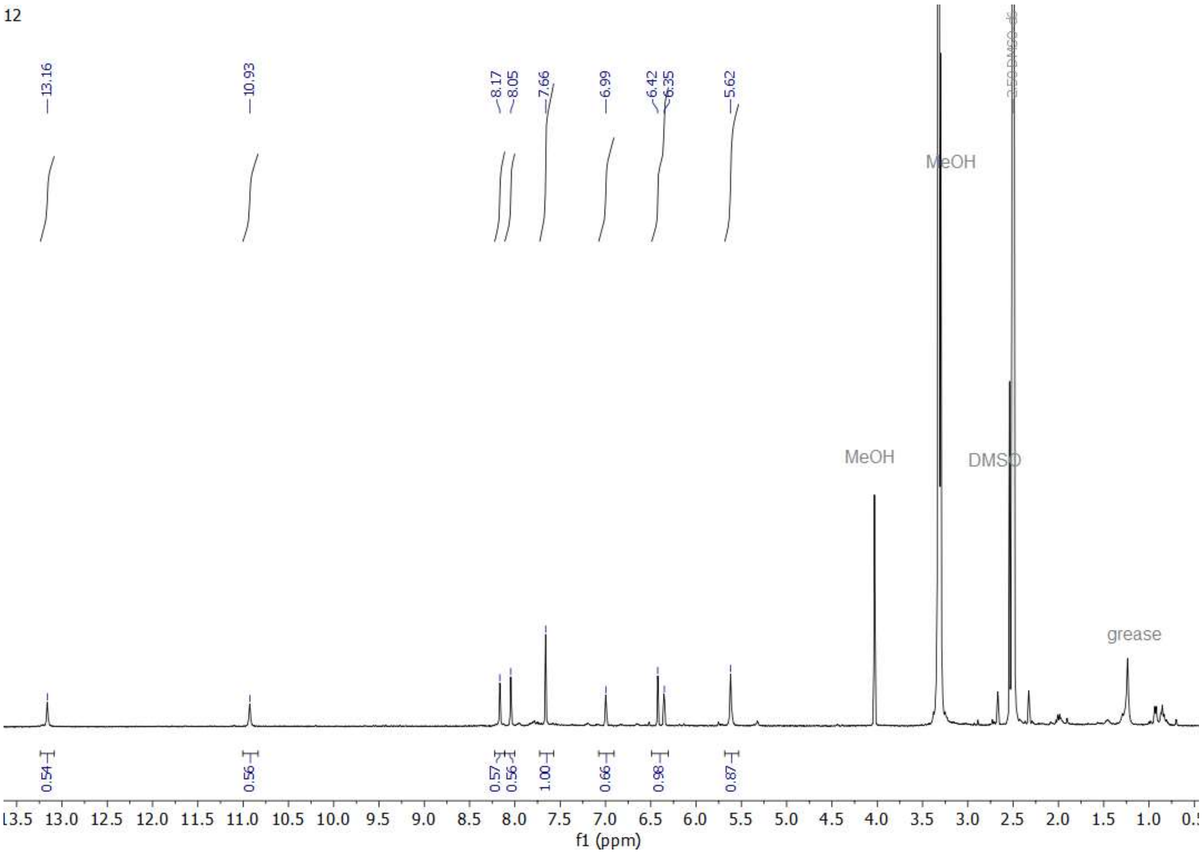

^{1}H NMR (400 MHz, DMSO-d_6, TMS) δ_H 13.16 (1H, s), 10.93 (1H, s), 8.17 (1H, s), 8.05 (1H, s), 7.66 (1H, s), 7.13 – 6.95 (1H, m), 6.44 – 6.33 (1H, m), 5.62 (1H, s).

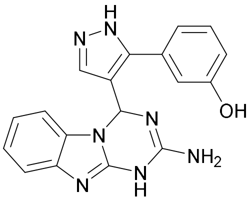

13

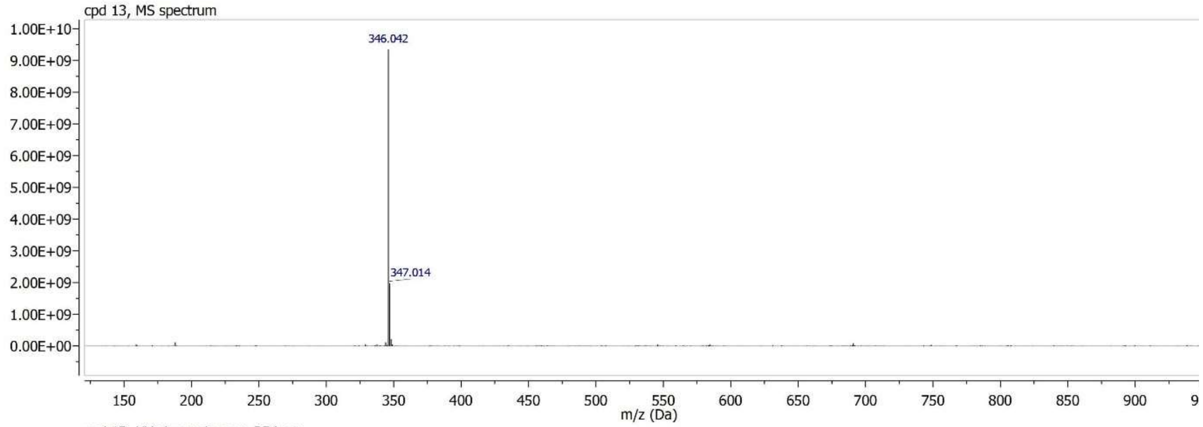

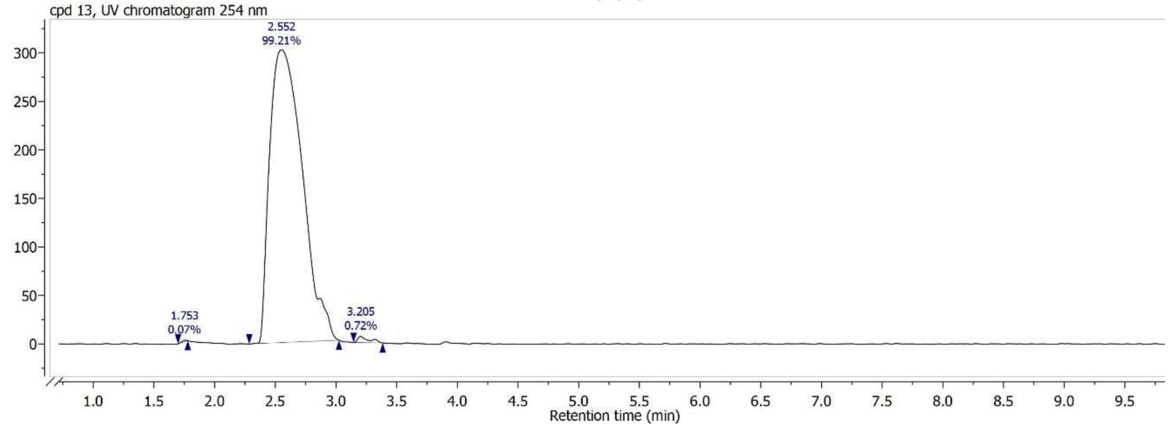

LC-ESI-MS (measured with Poroshell column, gradient 10–90% ACN in water + 0.1% formic acid over 10 min), top: mass spectrum, bottom: LC chromatogram at 254 nm. Calculated: m/z = 346.1 [M + H^+], found: m/z = 346.0[M + H^+], purity 99.2%.

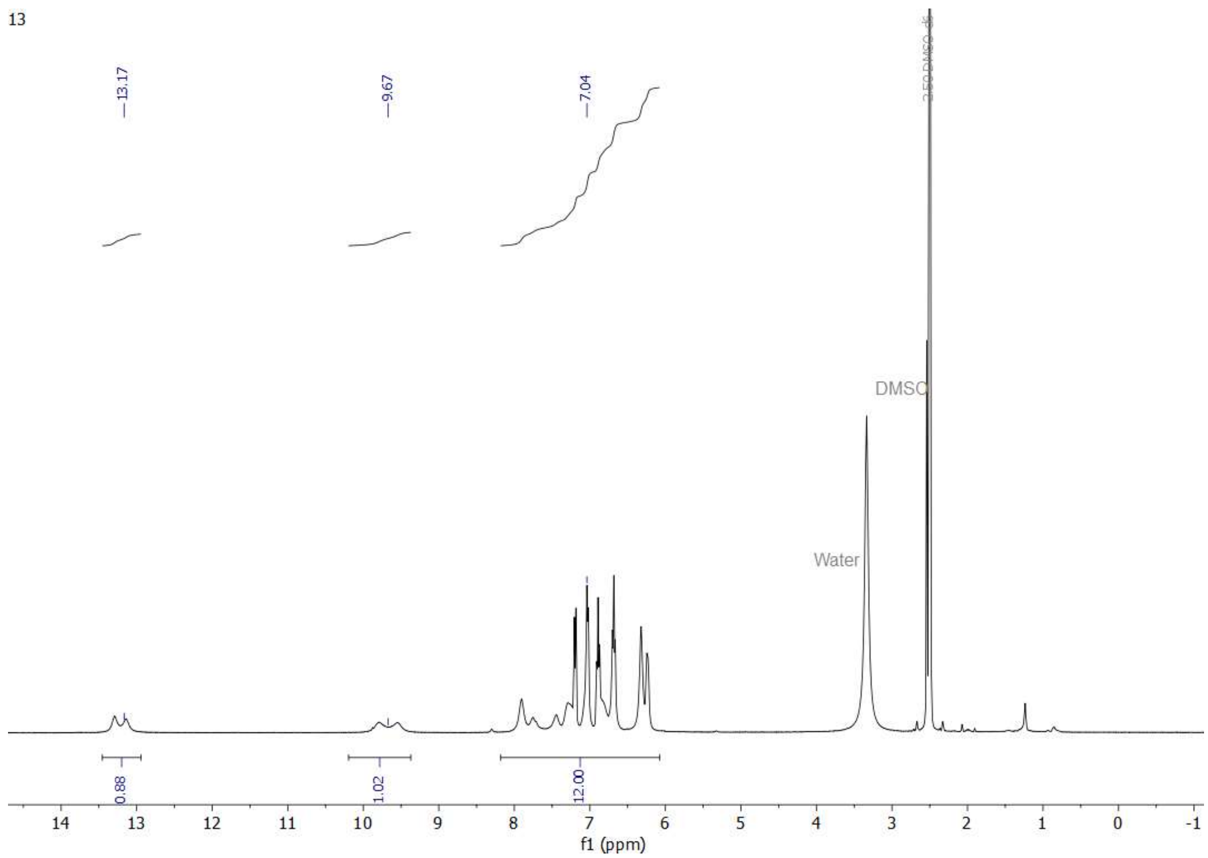

^{1}H NMR (400 MHz, DMSO-d_6, TMS) δ_H 13.61 – 12.91 (1H, m), 10.08 – 9.28 (1H, m), 8.25 – 5.99 (12H, m).

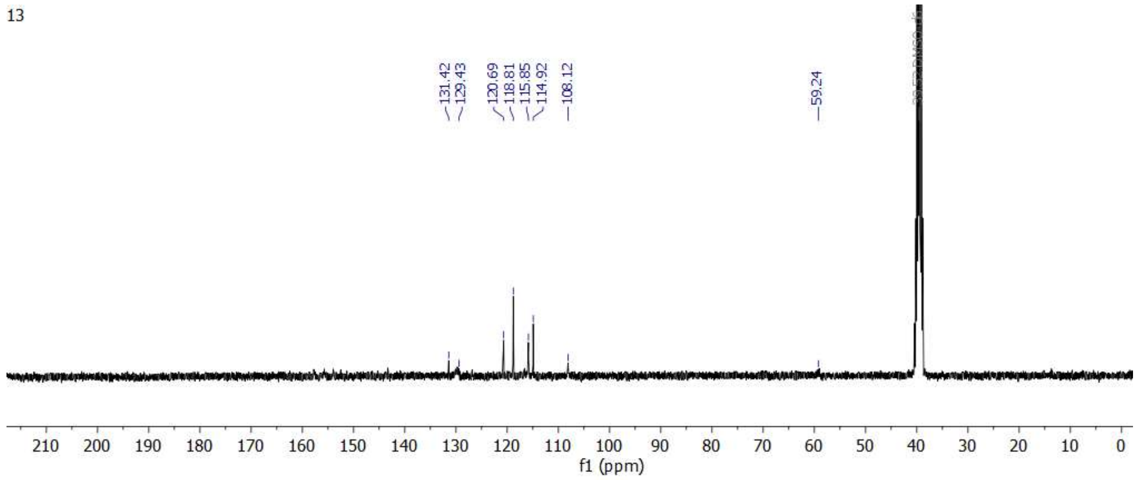

^{13}C NMR (101 MHz, DMSO-d_6, TMS) δ_C 131.42, 129.43, 120.69, 118.81, 115.85, 114.92, 108.12, 59.24.

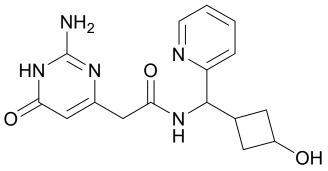

14

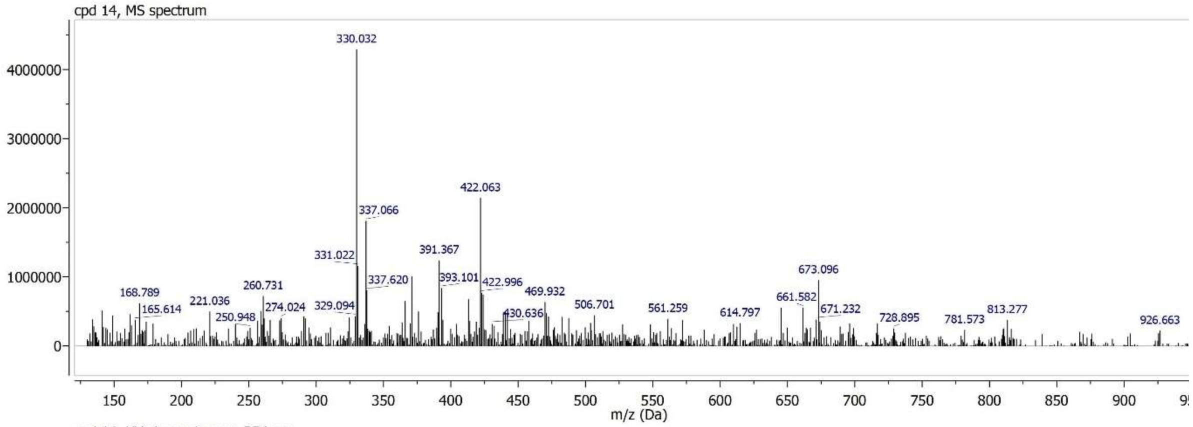

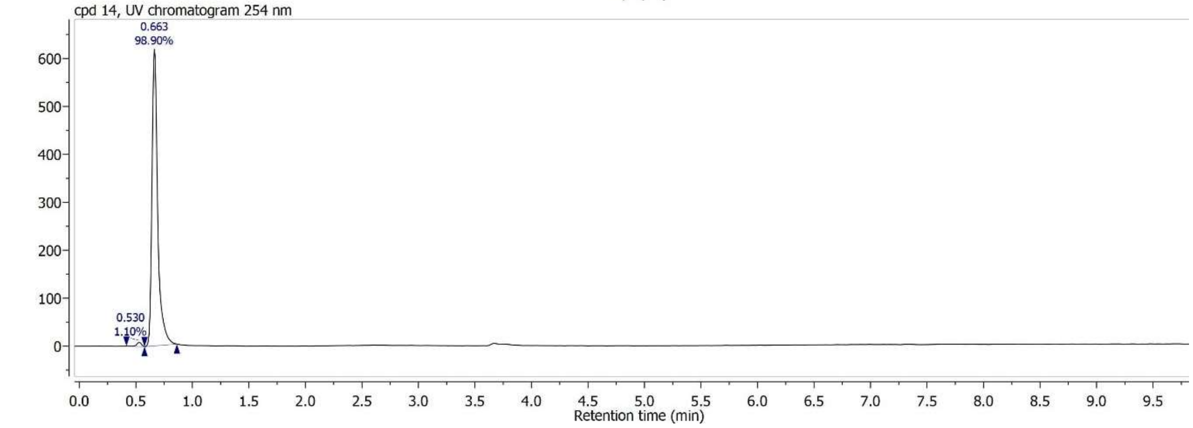

LC-ESI-MS (measured with Poroshell column, gradient 10–90% ACN in water + 0.1% formic acid over 10 min), top: mass spectrum, bottom: LC chromatogram at 254 nm. Calculated: m/z = 330.2 [M + H^+], found: m/z = 330.0 [M + H^+], purity 98.9%.

14

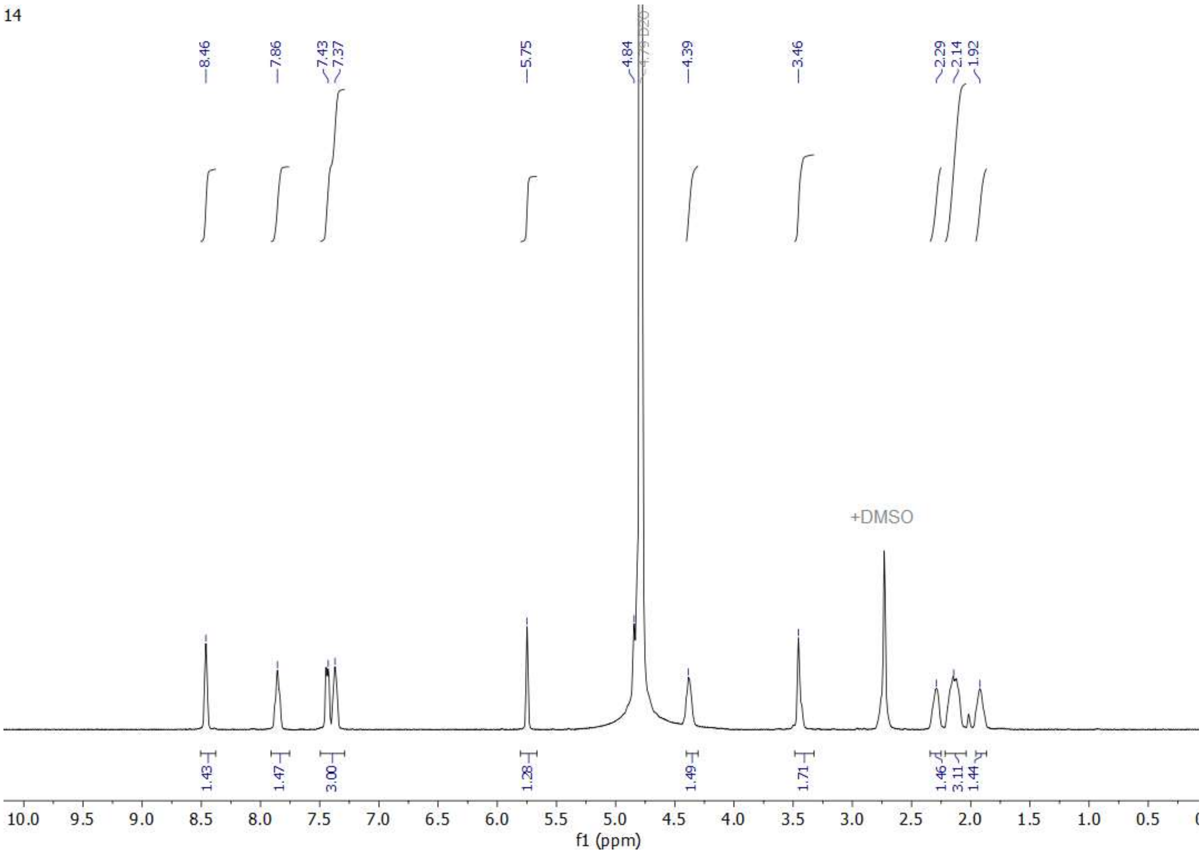

^{1}H NMR (400 MHz, Deuterium Oxide, TMS) δ_H 8.54 – 8.36 (1H, m), 7.95 – 7.78 (1H, m), 7.54 – 7.30 (3H, m), 5.75 (1H, s), 4.92 – 4.82 (m, 6H), 4.49 – 4.27 (1H, m), 3.52 – 3.35 (2H, m), 2.41 – 2.22 (1H, m), 2.22 – 2.03 (3H, m), 1.97 – 1.81 (1H, m).

14

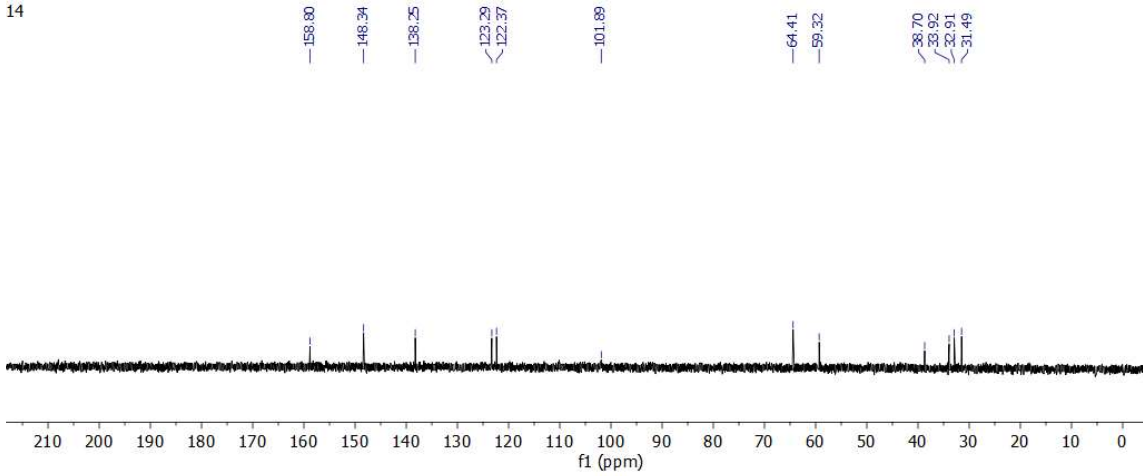

^{13}C NMR (101 MHz, Deuterium Oxide, TMS) δ_C 158.80, 148.34, 138.25, 123.29, 122.37, 101.89, 64.41, 59.32, 38.70, 33.92, 32.91, 31.49.

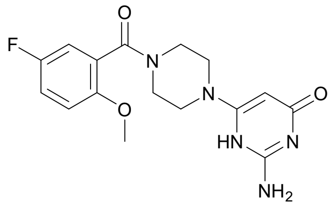

15

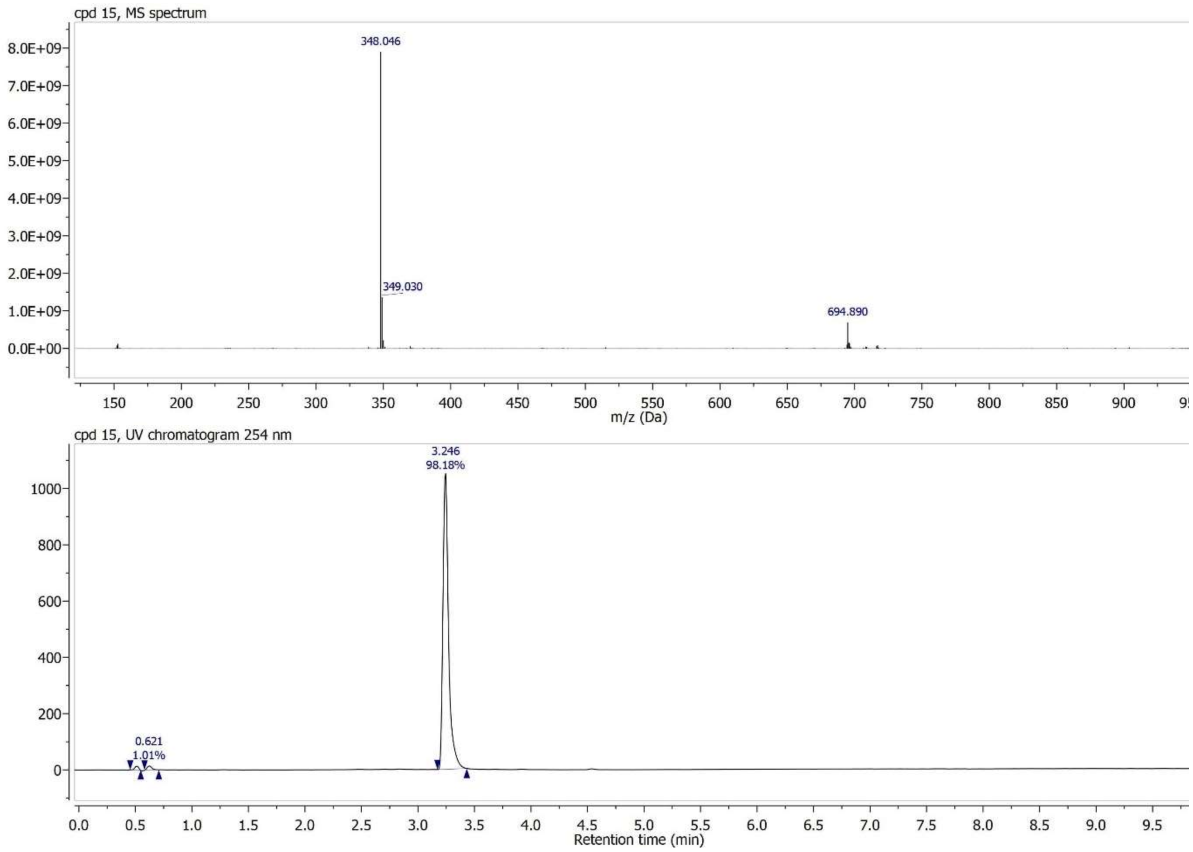

LC-ESI-MS (measured with Poroshell column, gradient 10–90% ACN in water + 0.1% formic acid over 10 min), top: mass spectrum, bottom: LC chromatogram at 254 nm. Calculated: m/z = 348.1 [M + H$^+$], found: m/z = 348.0 [M + H$^+$], purity 98.2%.

15

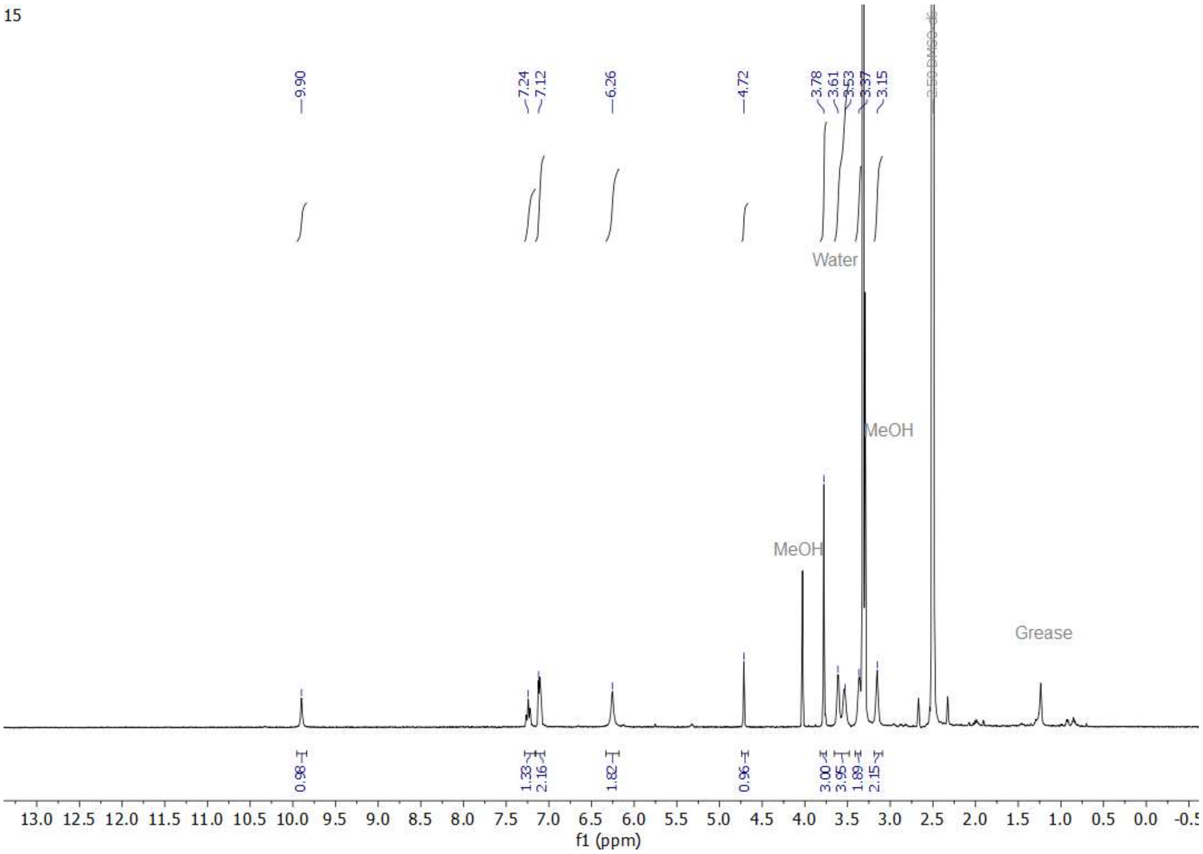

^{1}**H NMR** (400 MHz, DMSO-*d*$_6$, TMS) δ_H 9.90 (1H, s), 7.34 – 7.16 (1H, m), 7.17 – 7.02 (2H, m), 6.26 (2H, s), 4.72 (1H, s), 3.82 – 3.72 (3H, m), 3.63 – 3.49 (4H, m), 3.38 – 3.33 (2H, m), 3.22 – 3.08 (2H, m).

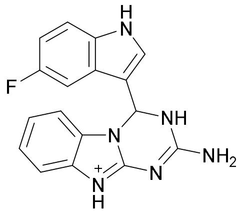

16

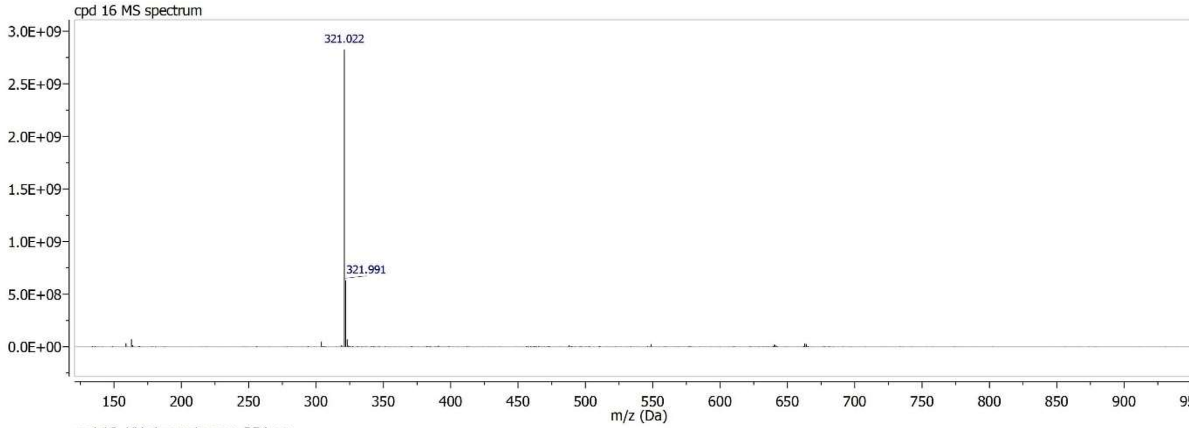

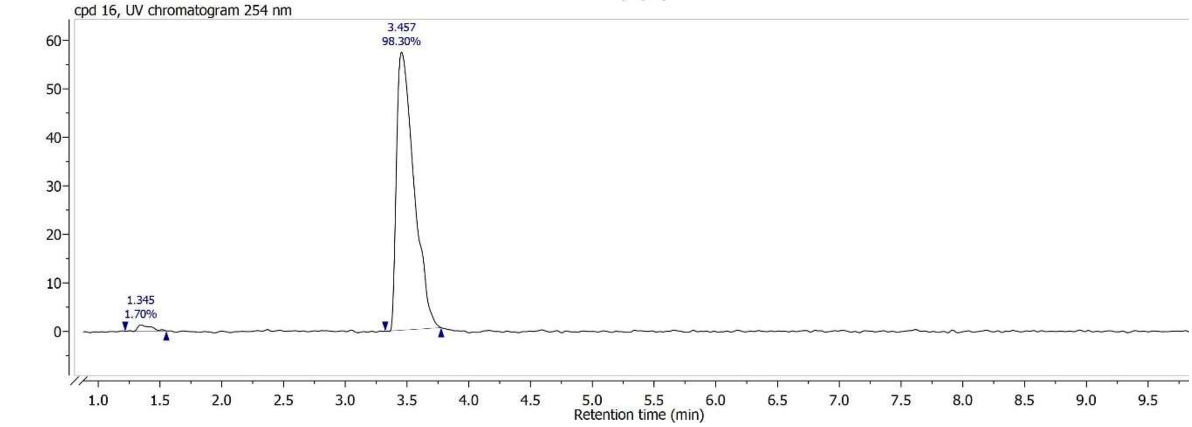

LC-ESI-MS (measured with Poroshell column, gradient 10–90% ACN in water + 0.1% formic acid over 10 min), top: mass spectrum, bottom: LC chromatogram at 254 nm. Calculated: m/z = 321.1 [M + H^+], found: m/z = 321.0 [M + H^+], purity 98.3%.

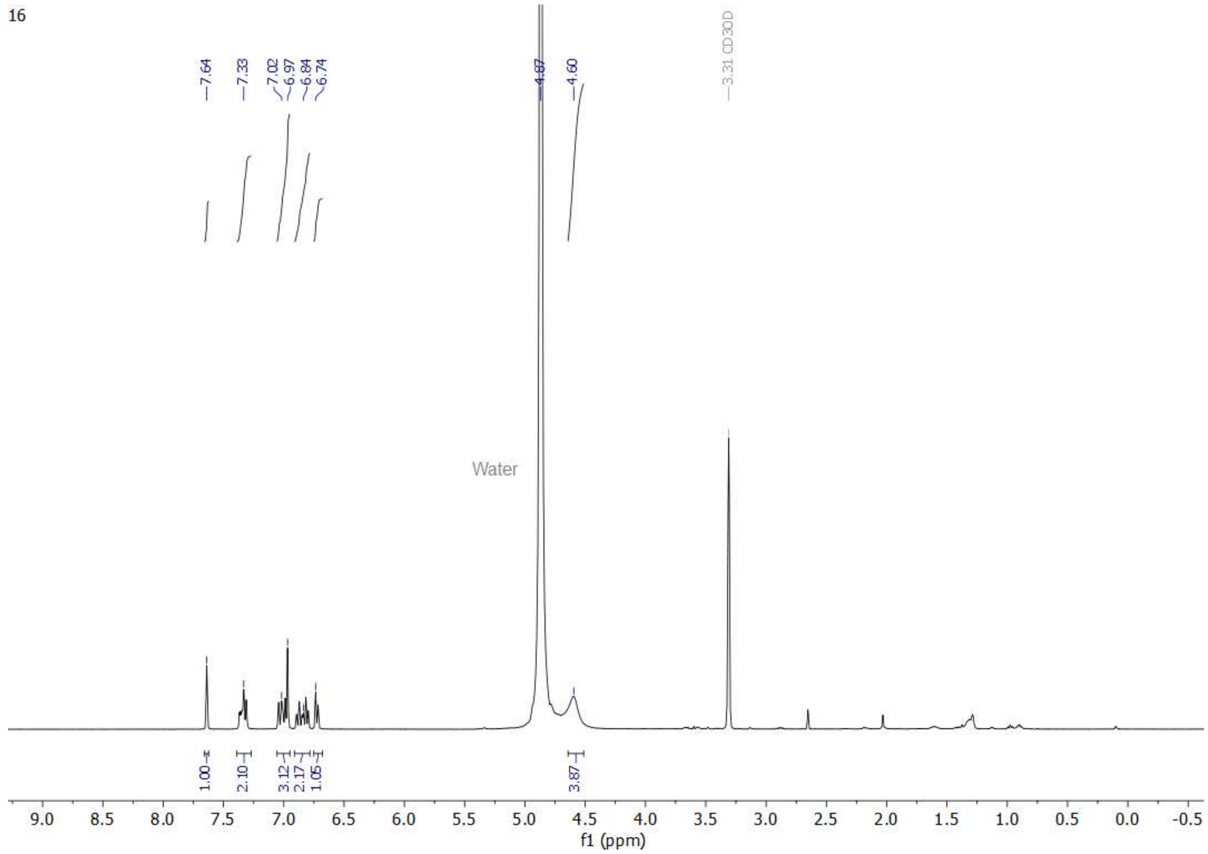

^{1}H NMR (400 MHz, Methanol-d_4, TMS) δ_H 7.64 (1H, s), 7.43 – 7.25 (2H, m), 7.11 – 6.93 (3H, m), 6.84 (2H, dt, J = 20.3, 8.3 Hz), 6.73 (1H, d, J = 8.0 Hz), 4.60 (4H, s).

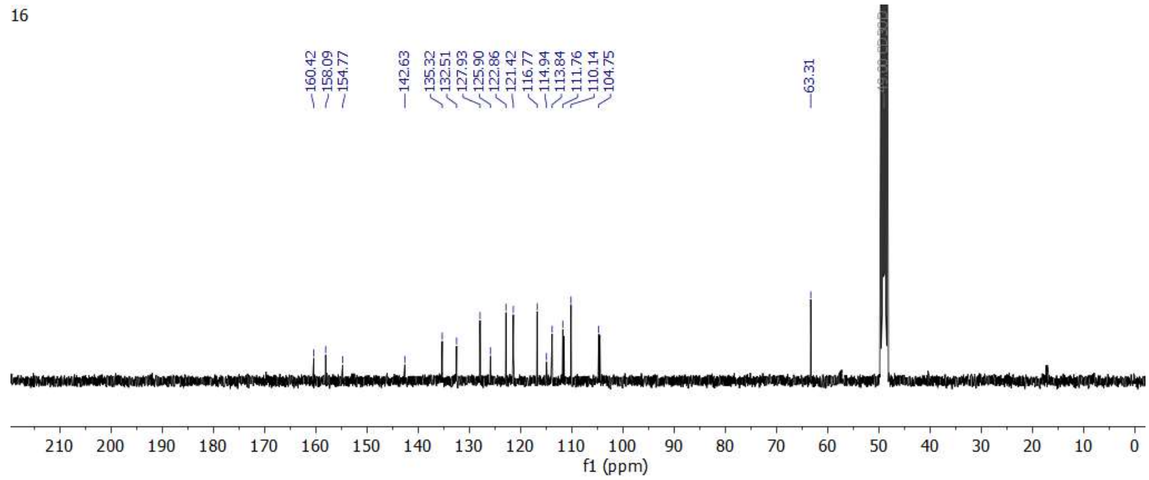

^{13}C NMR (101 MHz, Methanol-d_4, TMS) δ_C 160.42, 158.09, 154.77, 142.63, 135.32, 132.51, 127.93, 125.90, 122.86, 121.42, 116.77, 114.94, 113.84, 111.76, 110.14, 104.75, 63.31.

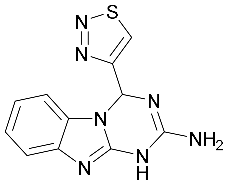

17

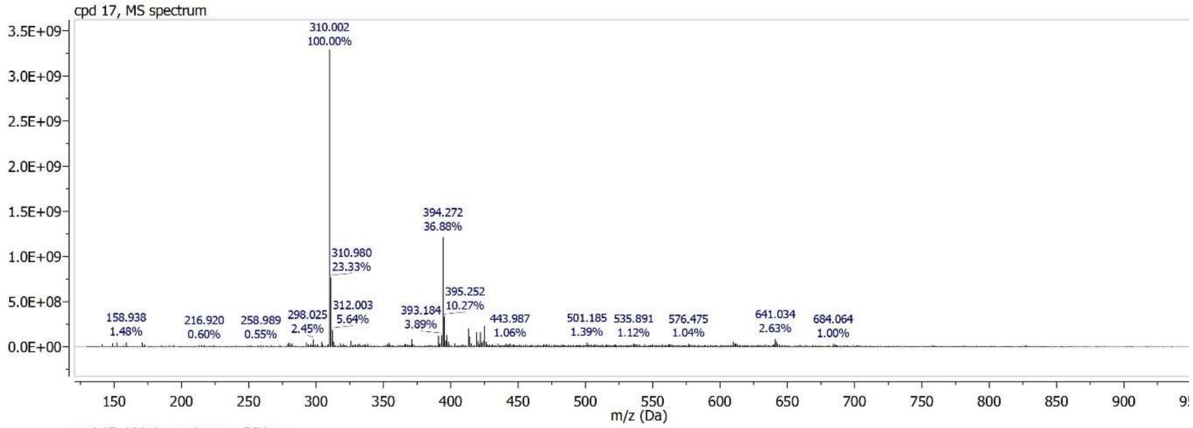

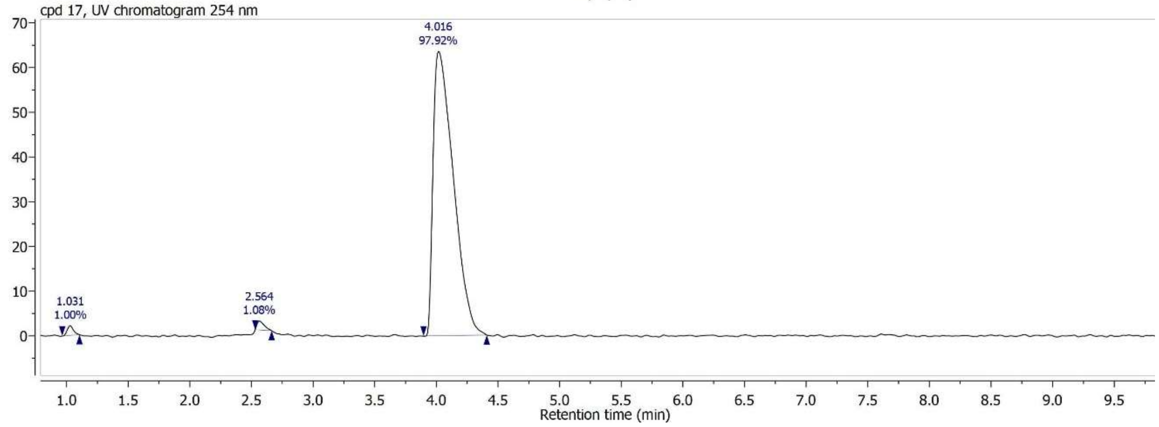

LC-ESI-MS (measured with Poroshell column, gradient 10–90% ACN in water + 0.1% formic acid over 10 min), top: mass spectrum, bottom: LC chromatogram at 254 nm. Calculated: m/z = 272.3 [M + H+], found: m/z = 310.0 [M + K+],, purity 97.9%.

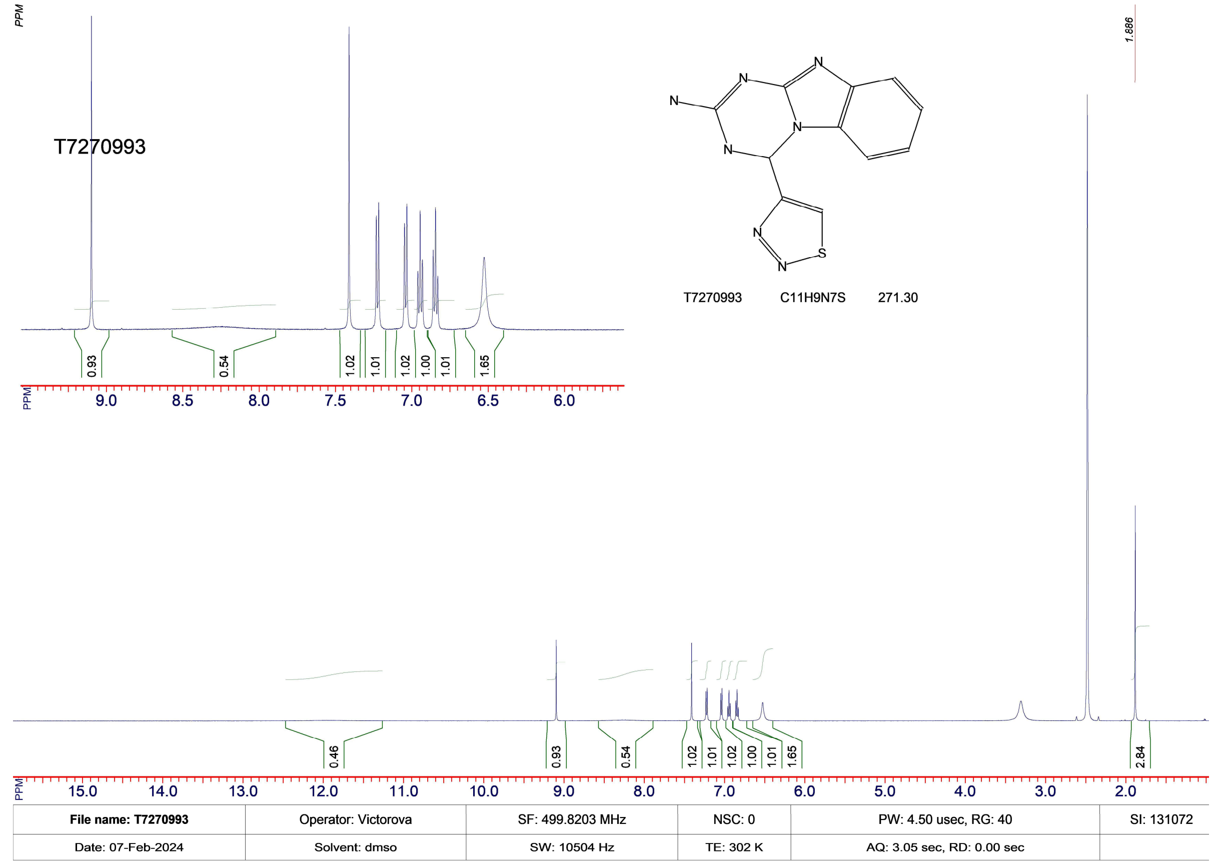

^{1}H NMR provided by Enamine Ltd.

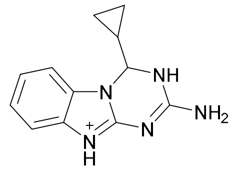

18

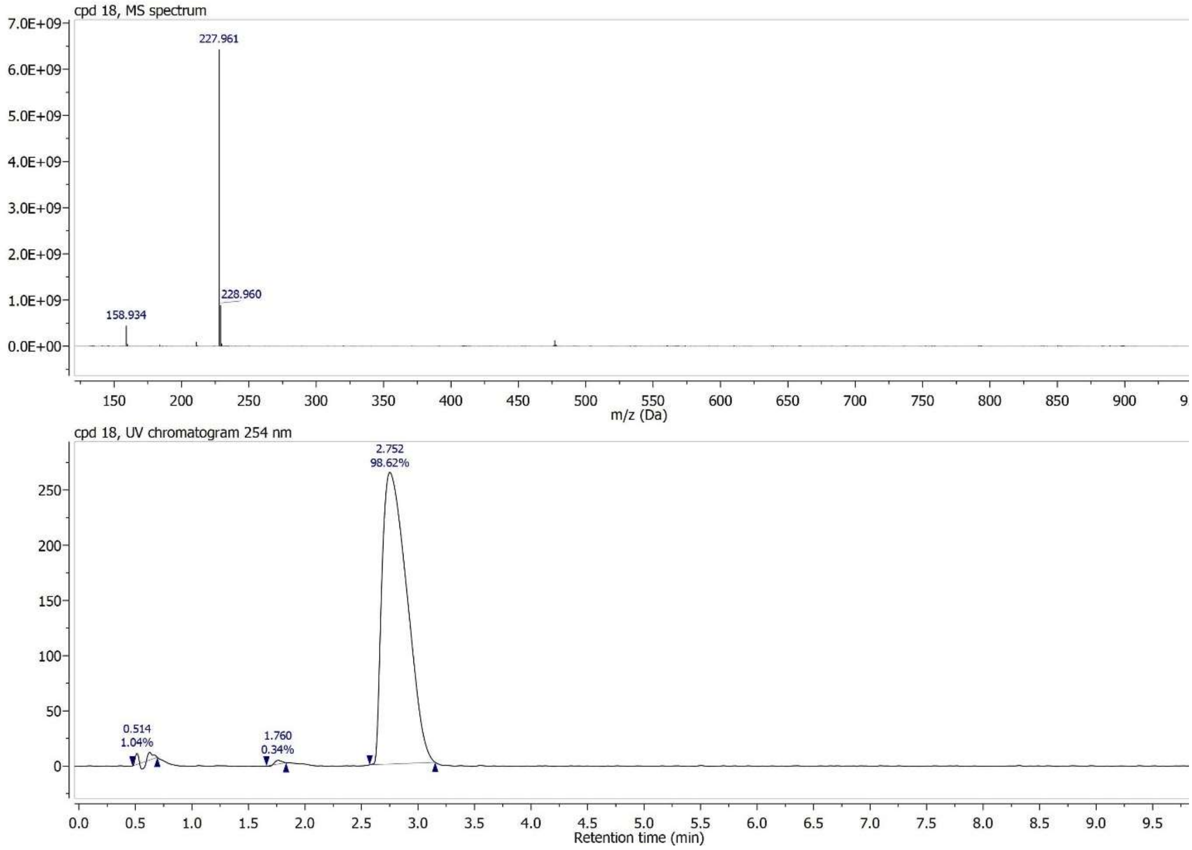

LC-ESI-MS (measured with Poroshell column, gradient 10–90% ACN in water + 0.1% formic acid over 10 min), top: mass spectrum, bottom: LC chromatogram at 254 nm. Calculated: m/z = 228.1 [M + H$^+$], found: m/z = 228.0 [M + H$^+$], purity 98.6%.

18

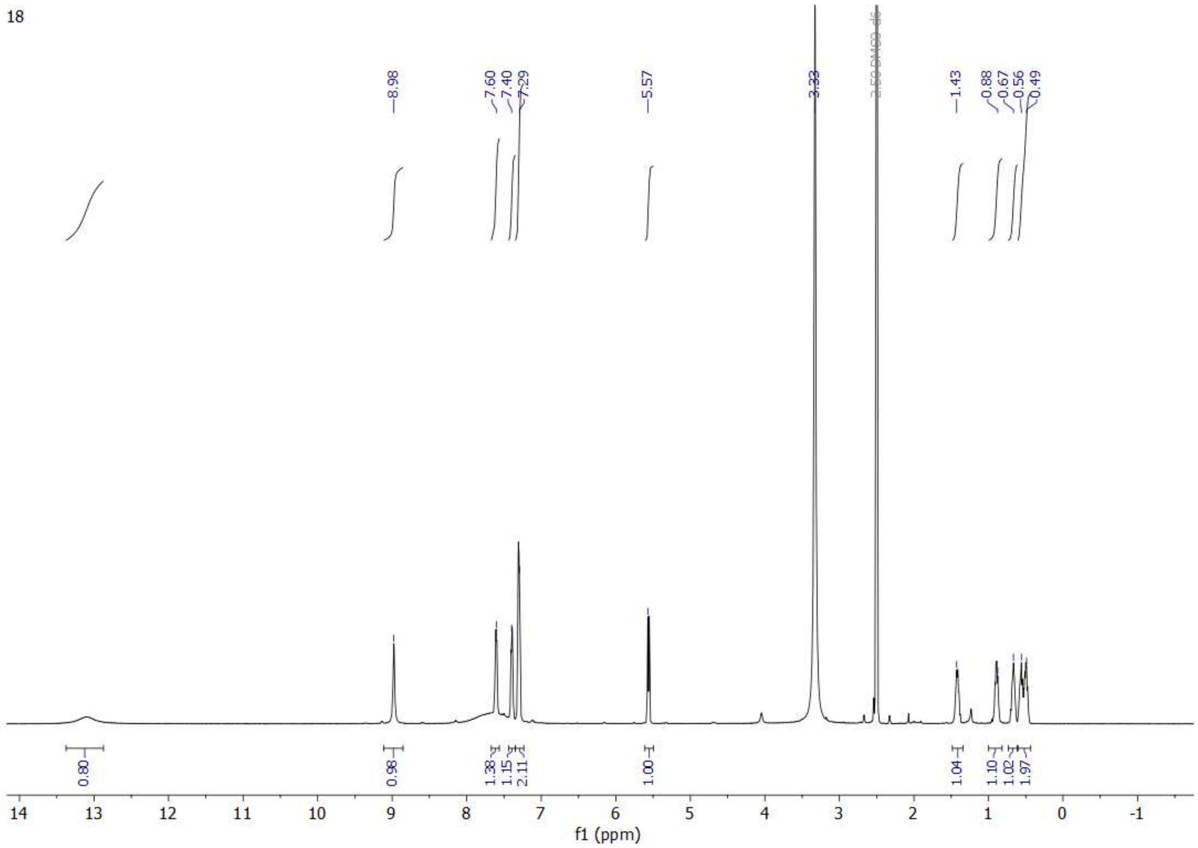

^{1}H NMR (400 MHz, DMSO-d_6, TMS) δ_H 13.10 (1H, s), 8.98 (1H, s), 7.73 – 7.53 (1H, m), 7.47 – 7.33 (1H, m), 7.34 – 7.18 (2H, m), 5.56 (1H, d, J = 7.6 Hz), 1.54 – 1.34 (1H, m), 1.02 – 0.83 (1H, m), 0.72 – 0.61 (1H, m), 0.61 – 0.43 (2H, m).

18

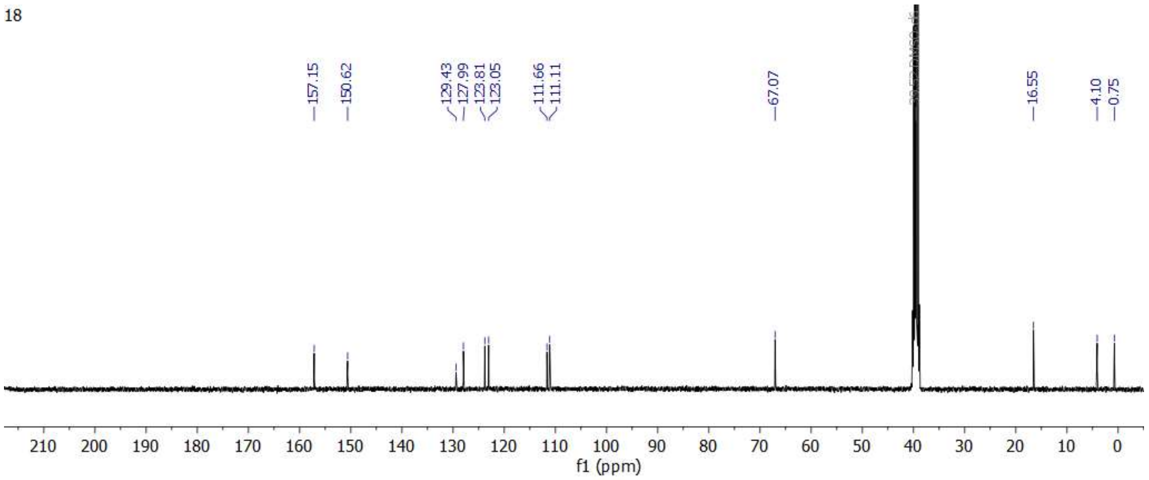

^{13}C NMR (101 MHz, DMSO-d_6, TMS) δ_C 157.15, 150.62, 129.43, 127.99, 123.81, 123.05, 111.66, 111.11, 67.07, 16.55, 4.10, 0.75.

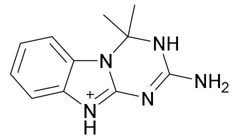

19

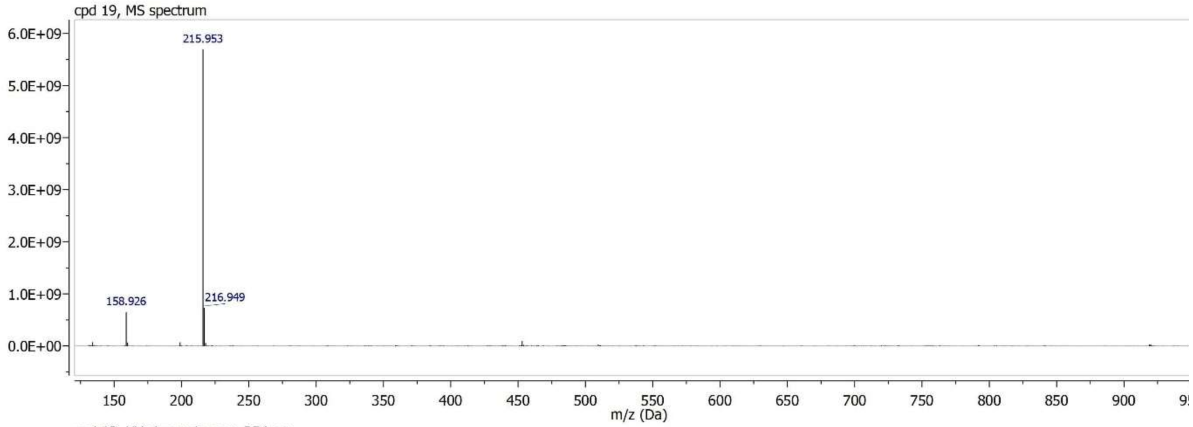

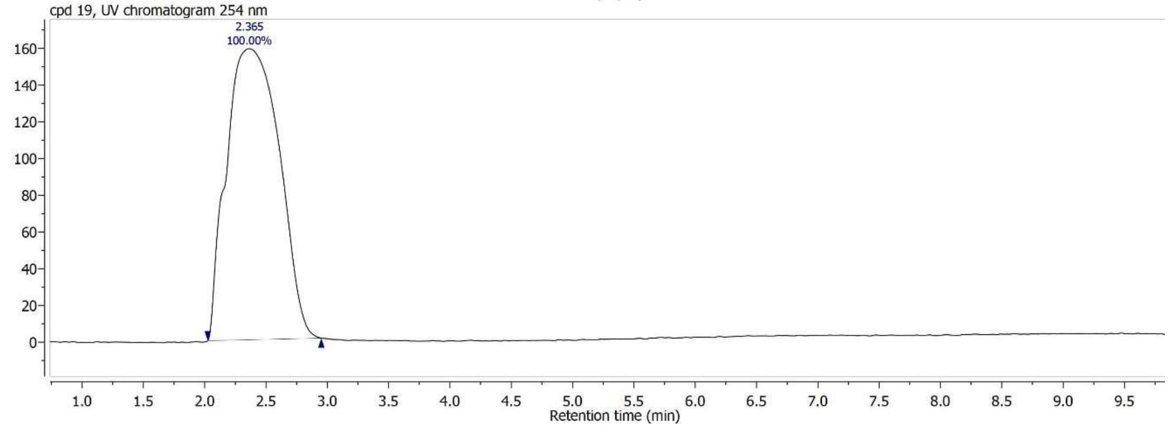

LC-ESI-MS (measured with Poroshell column, gradient 10–90% ACN in water + 0.1% formic acid over 10 min), top: mass spectrum, bottom: LC chromatogram at 254 nm. Calculated: m/z = 216.1 [M + H$^+$], found: m/z = 216.0 [M + H$^+$], purity 100.0%.

19

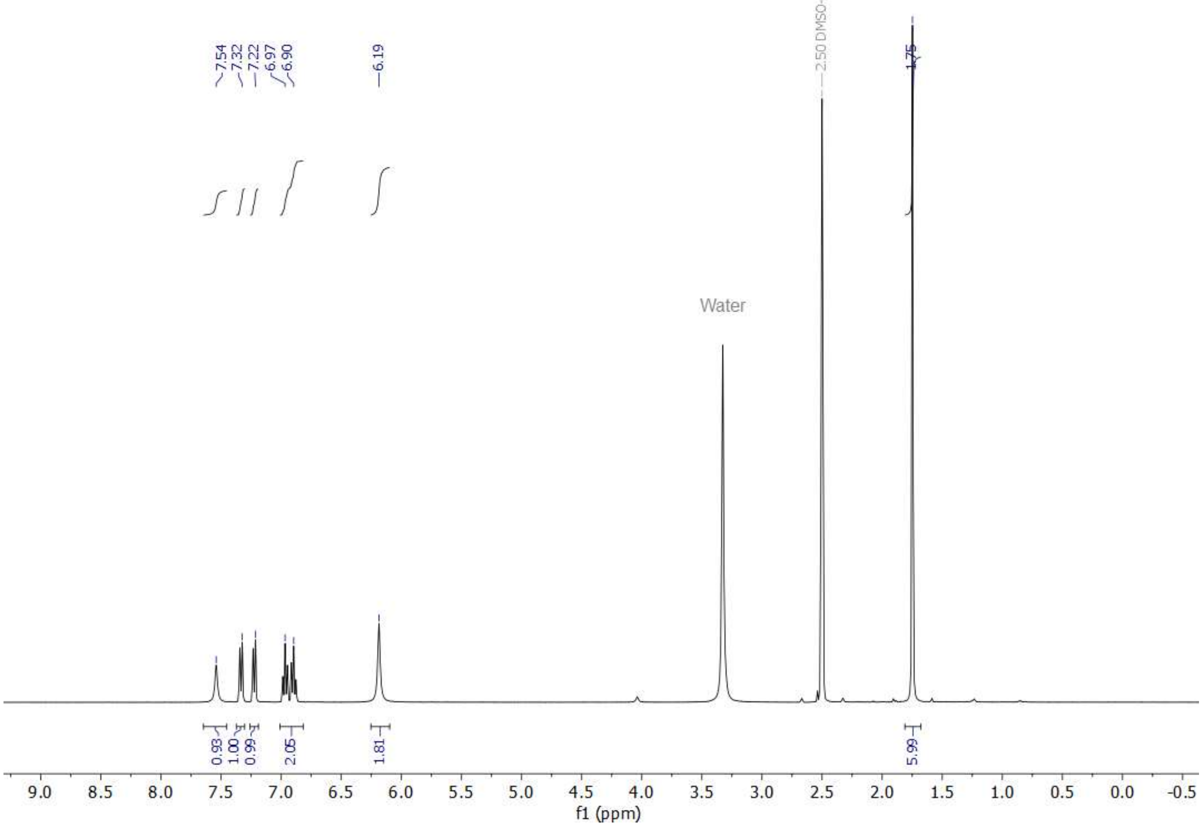

^{1}H NMR (400 MHz, DMSO-*d*$_6$, TMS) δ_H 7.54 (1H, s), 7.33 (1H, d, *J* = 7.8 Hz), 7.22 (1H, d, *J* = 7.7 Hz), 7.03 – 6.79 (2H, m), 6.19 (2H, s), 1.75 (6H, s).

19

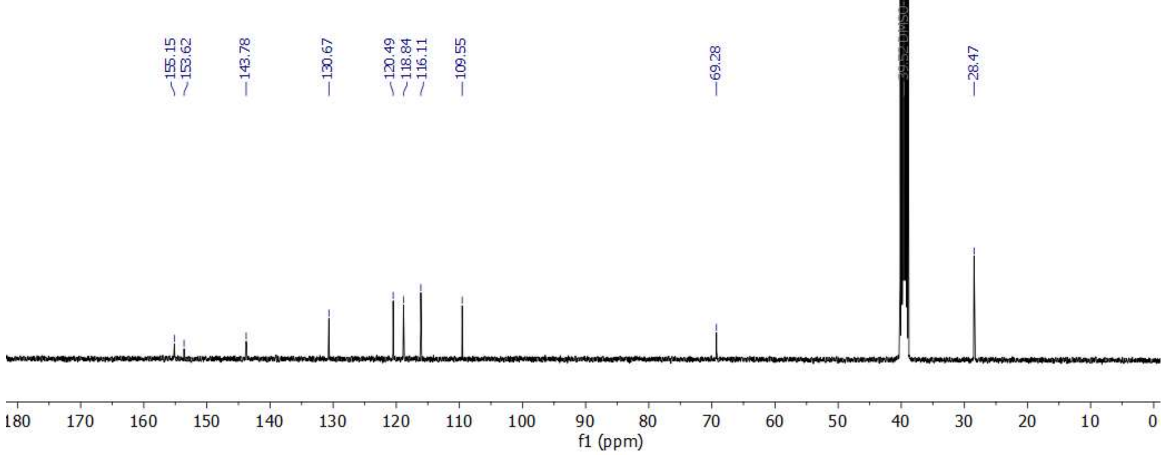

^{13}C NMR (101 MHz, DMSO-*d*$_6$, TMS) δ_C 155.15, 153.62, 143.78, 130.67, 120.49, 118.84, 116.11, 109.55, 69.28, 28.47.

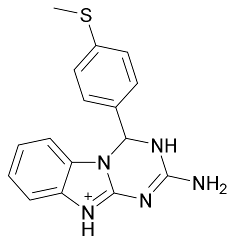

20

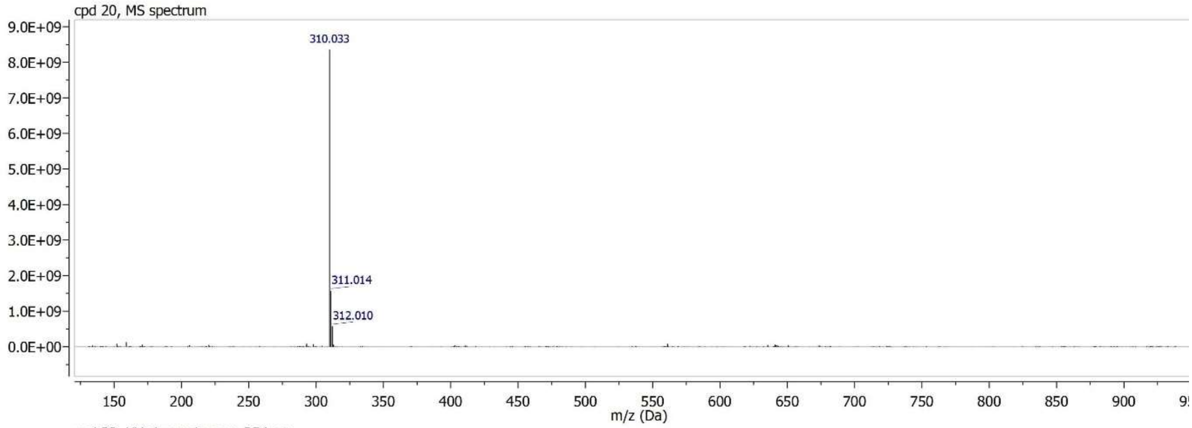

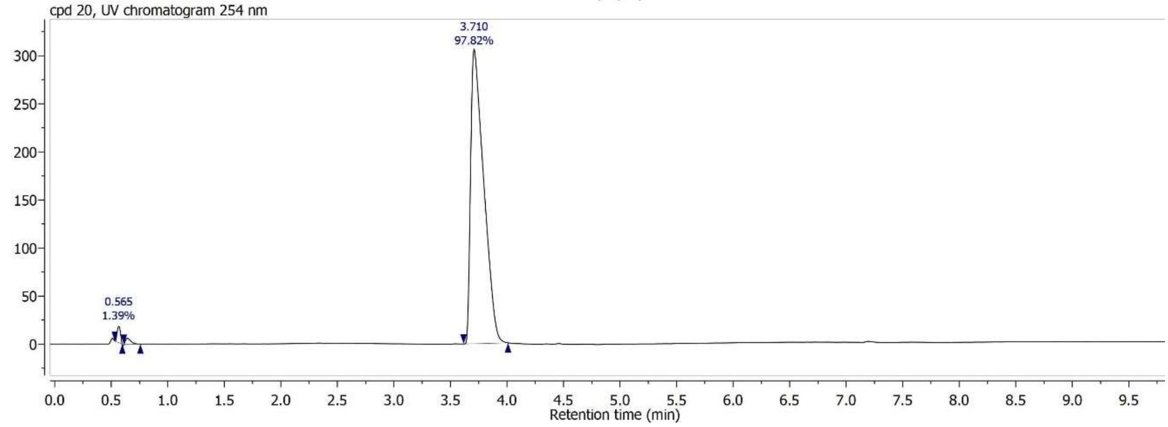

LC-ESI-MS (measured with Poroshell column, gradient 10–90% ACN in water + 0.1% formic acid over 10 min), top: mass spectrum, bottom: LC chromatogram at 254 nm. Calculated: m/z = 310.4 [M + H$^+$], found: m/z = 310.0 [M + H$^+$], purity 97.8%.

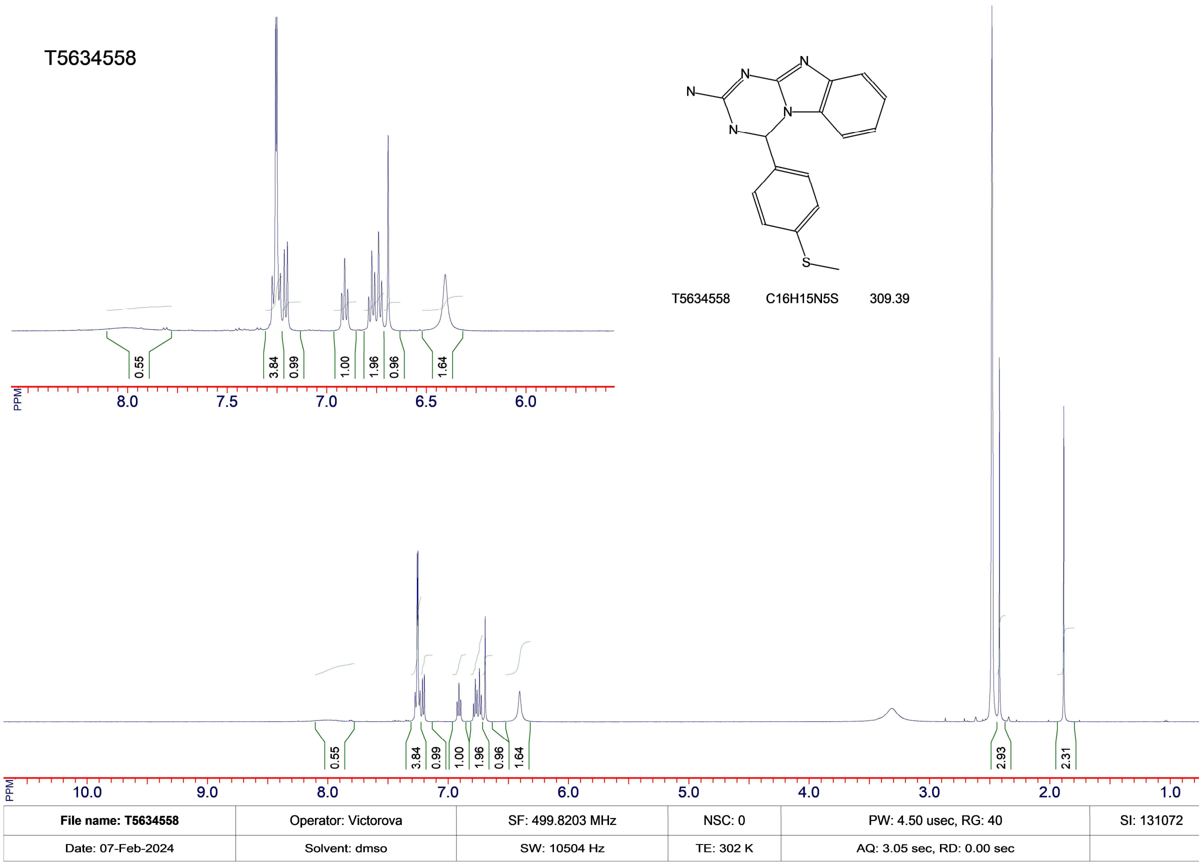

^{1}H NMR provided by Enamine Ltd.

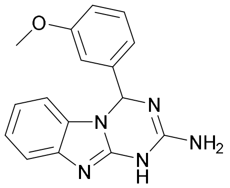

21

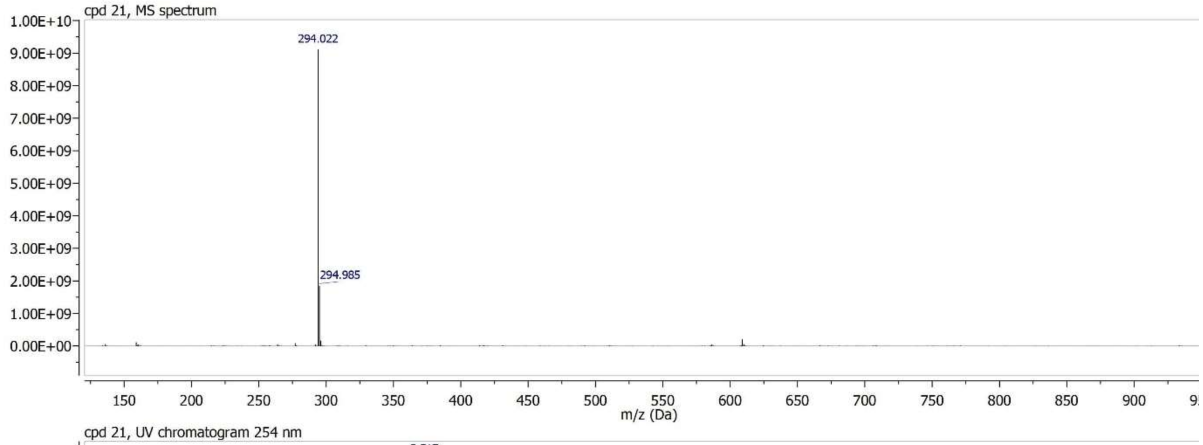

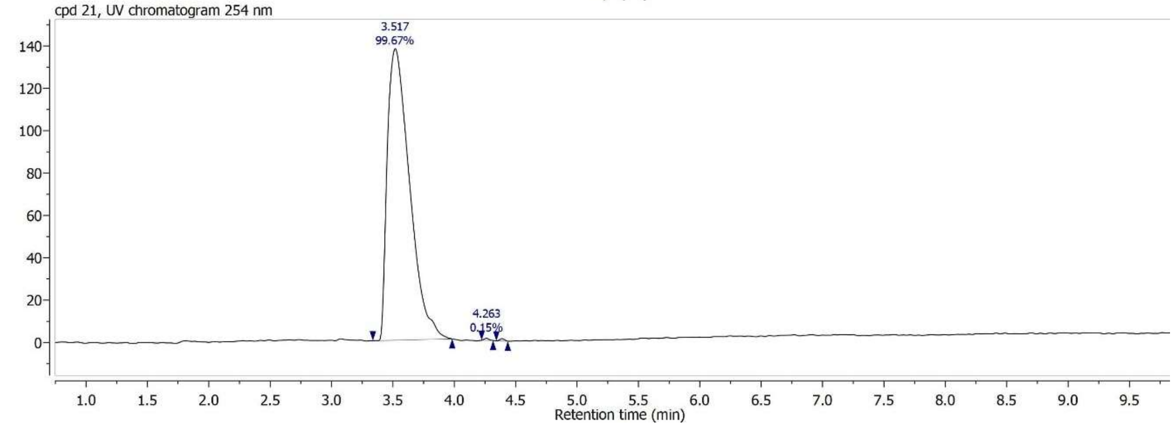

LC-ESI-MS (measured with Poroshell column, gradient 10–90% ACN in water + 0.1% formic acid over 10 min), top: mass spectrum, bottom: LC chromatogram at 254 nm. Calculated: m/z = 294.1 [M + H$^+$], found: m/z = 294.0 [M + H$^+$], purity 99.7%.

21

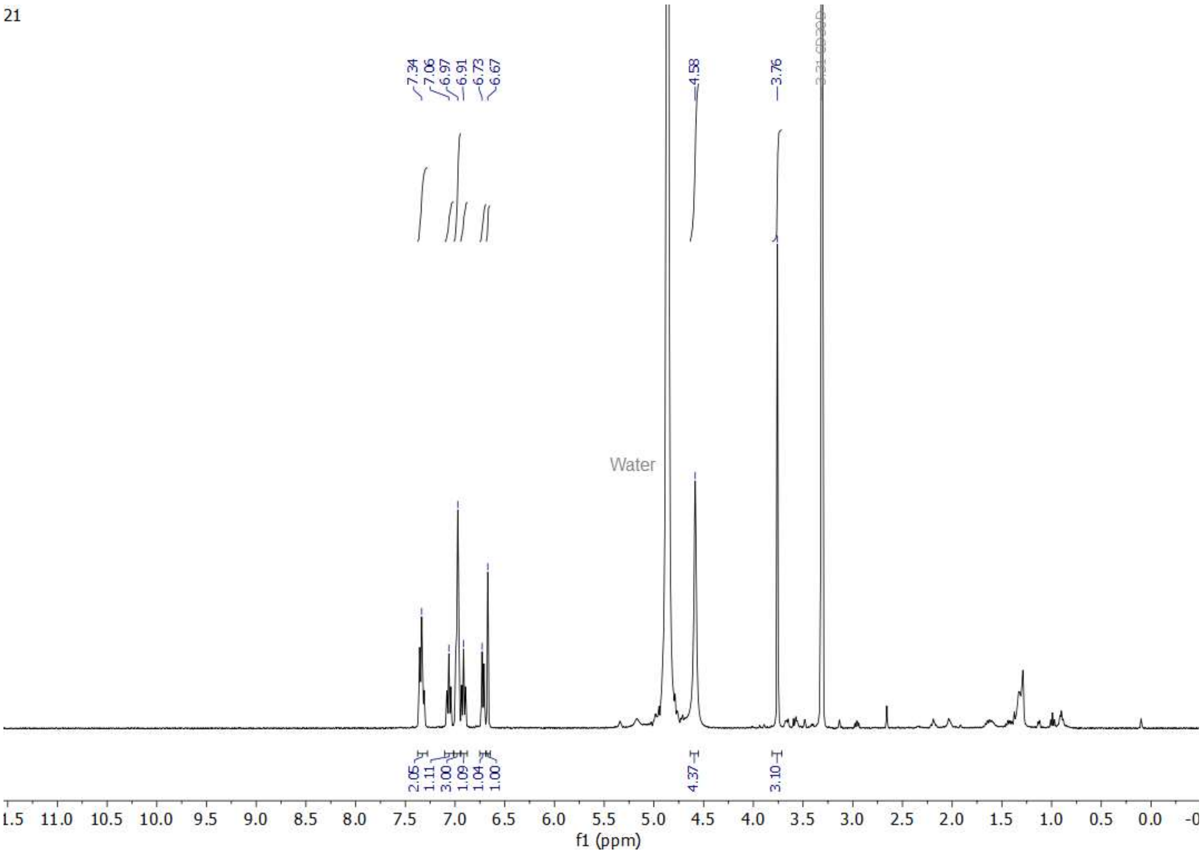

^{1}**H NMR** (400 MHz, Methanol-d_4, TMS) δ_H 7.38 – 7.29 (2H, m), 7.06 (1H, t, J = 7.7 Hz), 7.01 – 6.94 (3H, m), 6.91 (1H, t, J = 7.8 Hz), 6.72 (1H, d, J = 8.0 Hz), 6.67 (1H, d, J = 1.9 Hz), 4.58 (4H, s), 3.76 (3H, s).

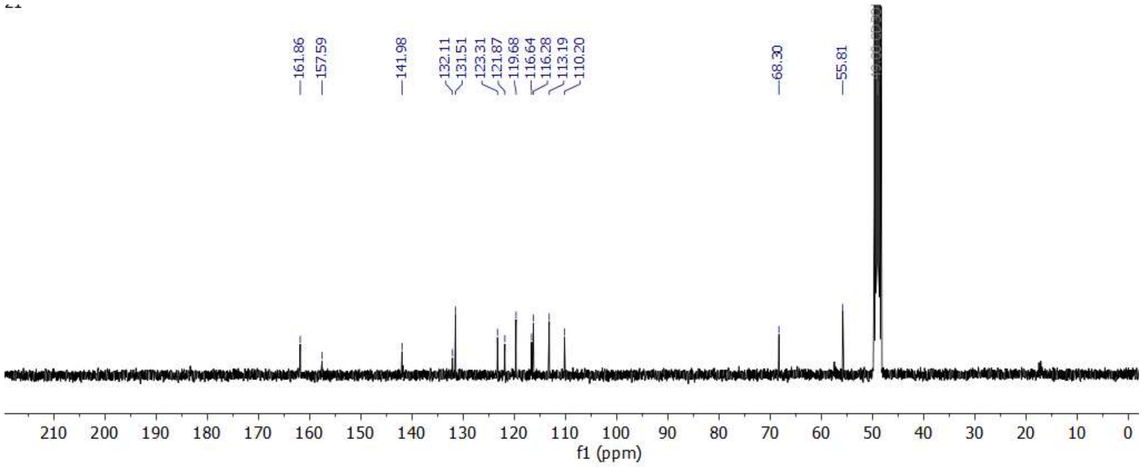

^{13}**C NMR** (101 MHz, Methanol-d_4, TMS) δ_C 161.86, 157.59, 141.98, 132.11, 131.51, 123.31, 121.87, 119.68, 116.64, 116.28, 113.19, 110.20, 68.30, 55.81.

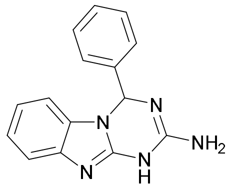

22

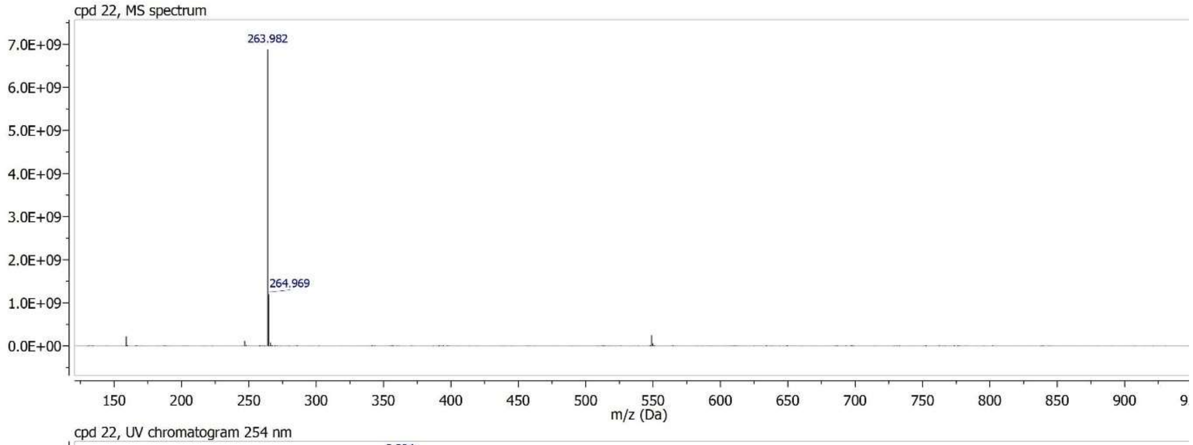

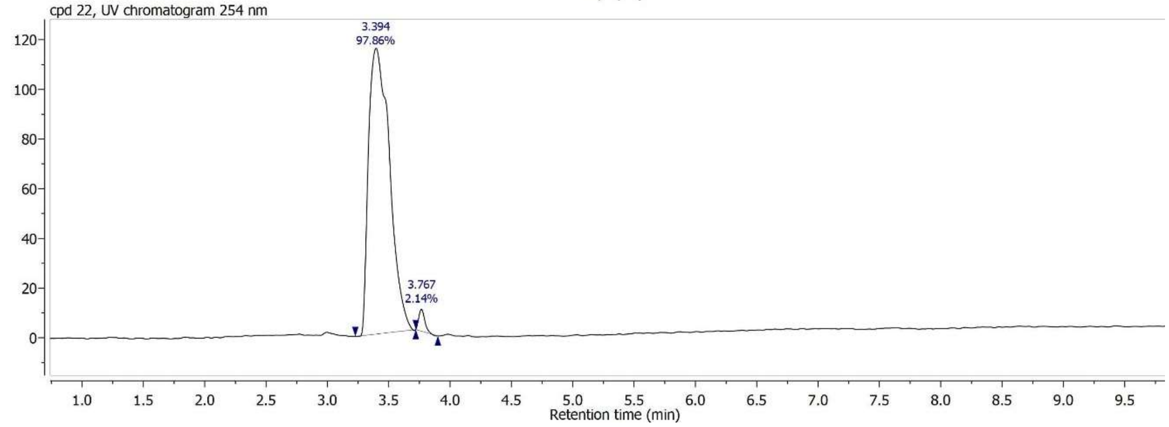

LC-ESI-MS (measured with Poroshell column, gradient 10–90% ACN in water + 0.1% formic acid over 10 min), top: mass spectrum, bottom: LC chromatogram at 254 nm. Calculated: m/z = 264.1 [M + H$^+$], found: m/z = 264.0 [M + H$^+$], purity 97.9%.

22

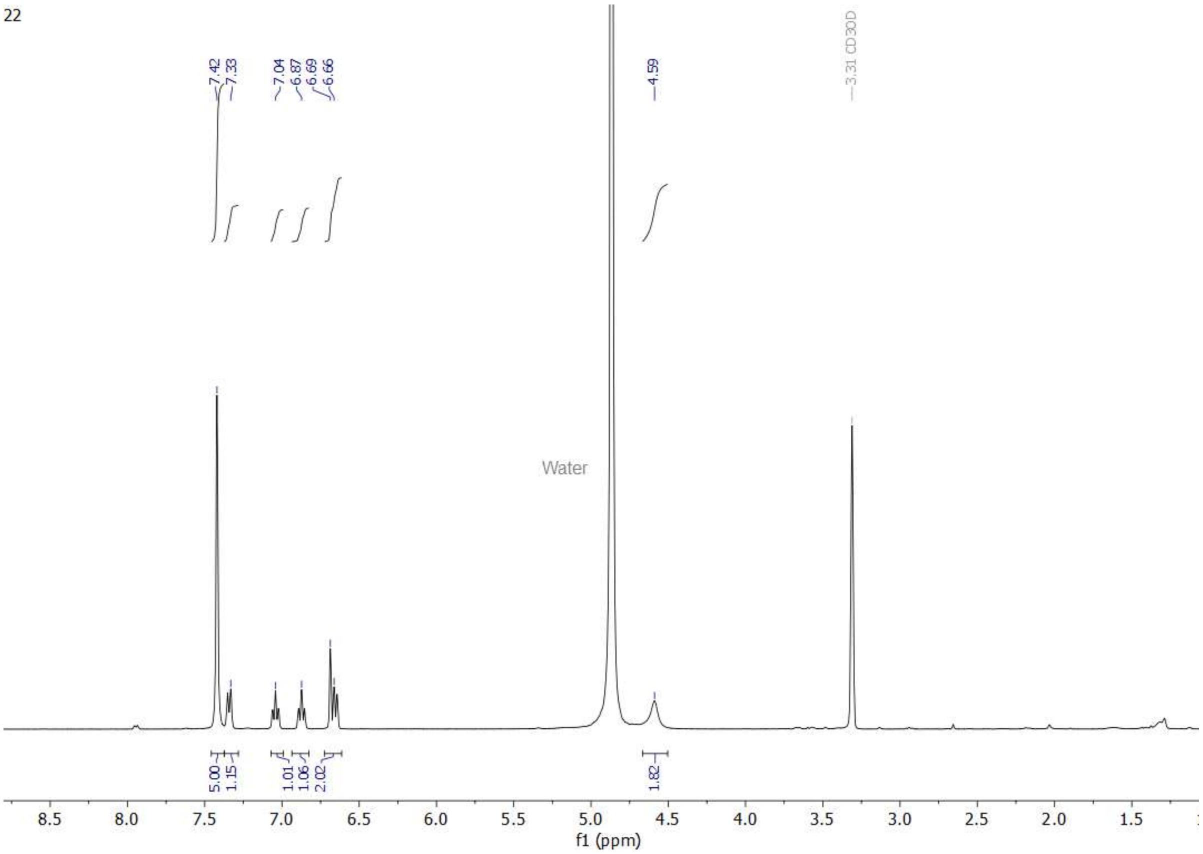

¹H NMR (400 MHz, Methanol-d_4, TMS) δ_H 7.48 – 7.37 (5H, m), 7.34 (1H, d, *J* = 8.0 Hz), 7.13 – 6.96 (1H, m), 6.94 – 6.78 (1H, m), 6.74 – 6.58 (2H, m), 4.59 (2H, s).

22

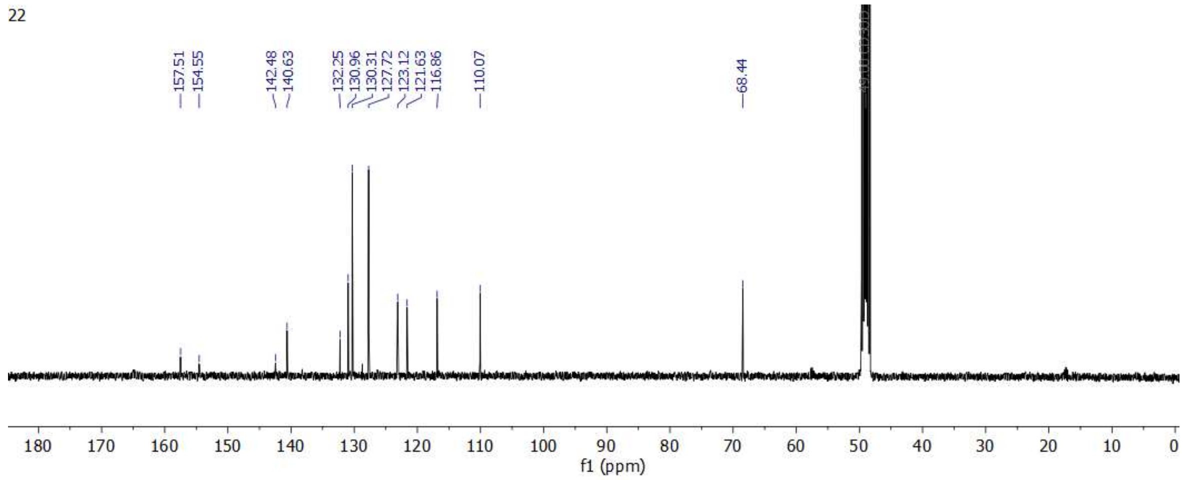

¹³C NMR (101 MHz, Methanol-d_4, TMS) δ_C 157.51, 154.55, 142.48, 140.63, 132.25, 130.96, 130.31, 127.72, 123.12, 121.63, 116.86, 110.07, 68.44.

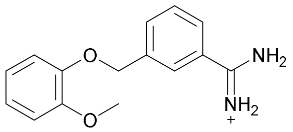

23

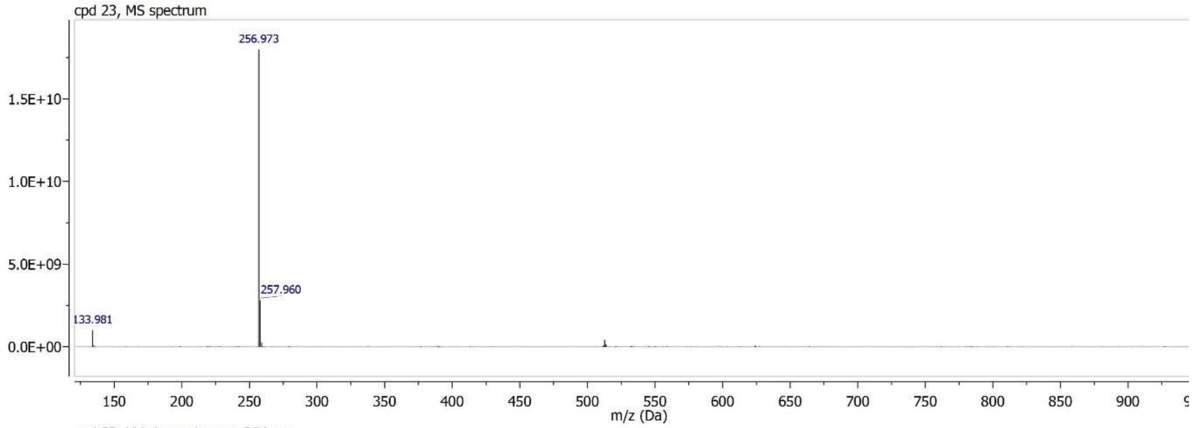

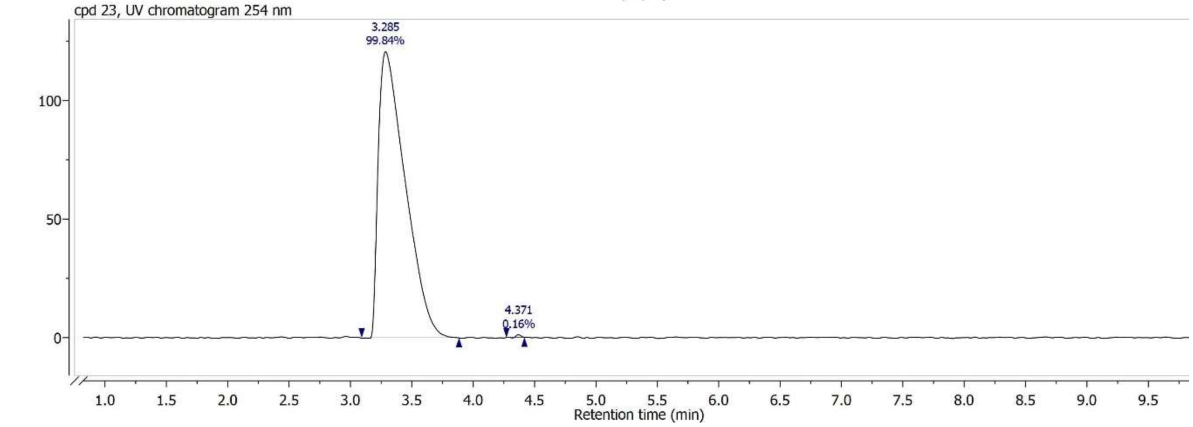

LC-ESI-MS (measured with Poroshell column, gradient 10–90% ACN in water + 0.1% formic acid over 10 min), top: mass spectrum, bottom: LC chromatogram at 254 nm. Calculated: m/z = 257.1 [M + H$^+$], found: m/z = 257.0 [M + H$^+$], purity 99.8%.

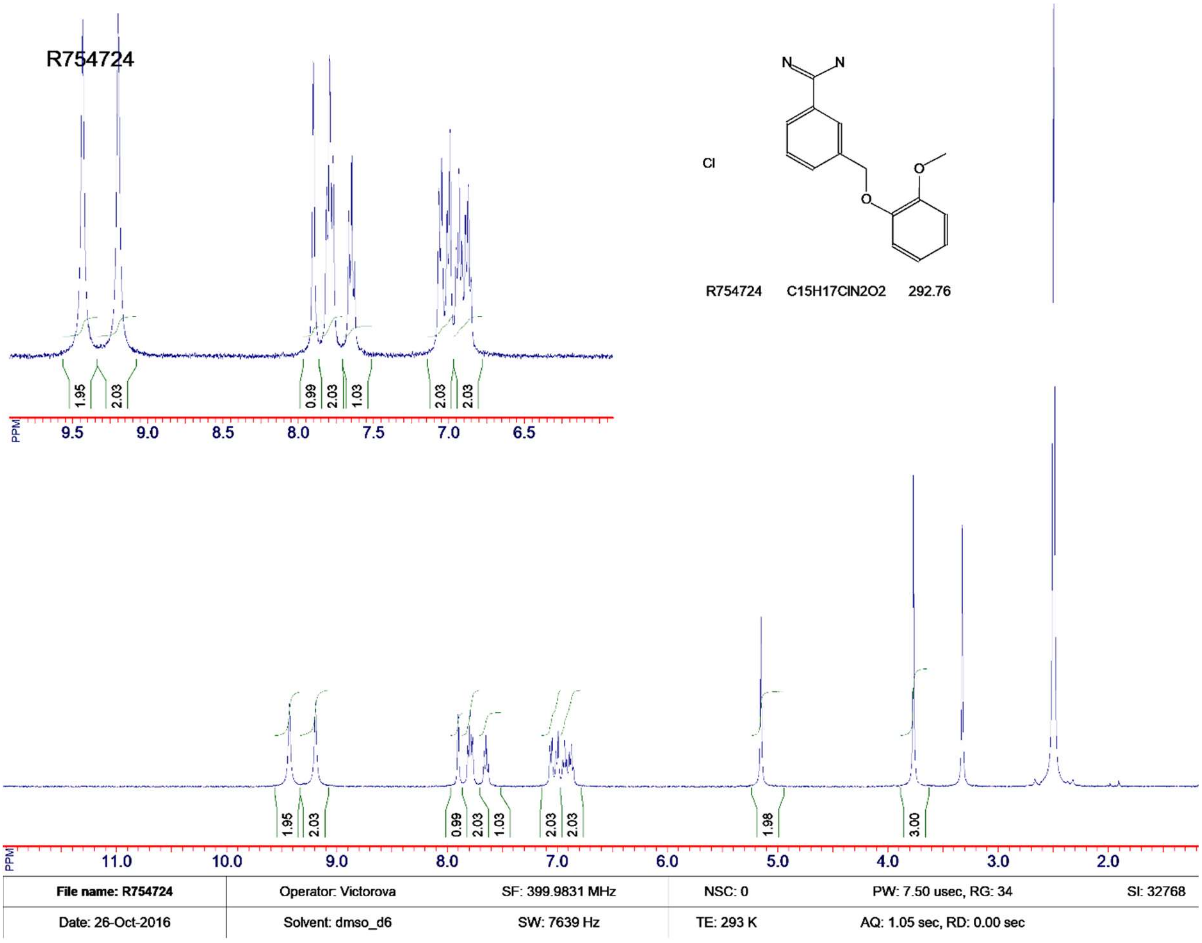

^{1}H NMR provided by Enamine Ltd.

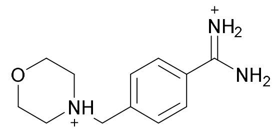

24

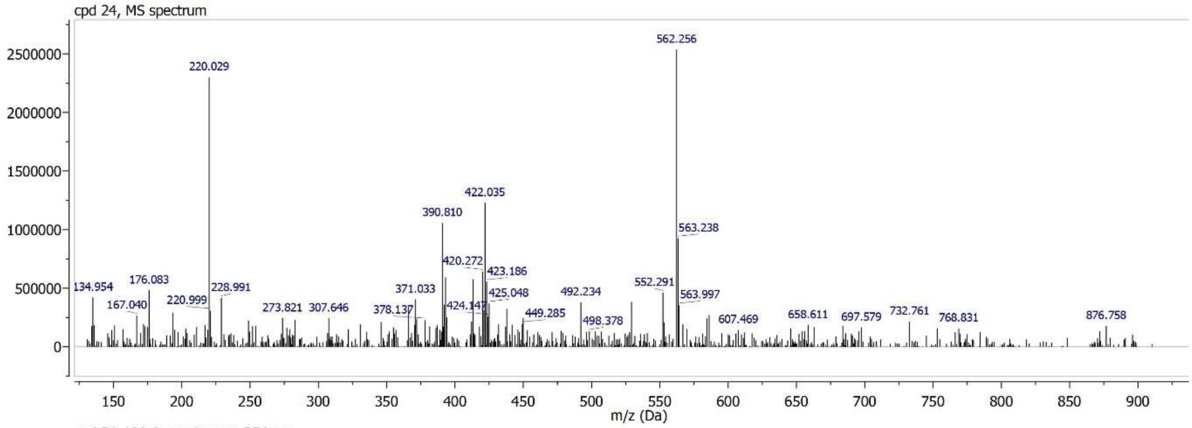

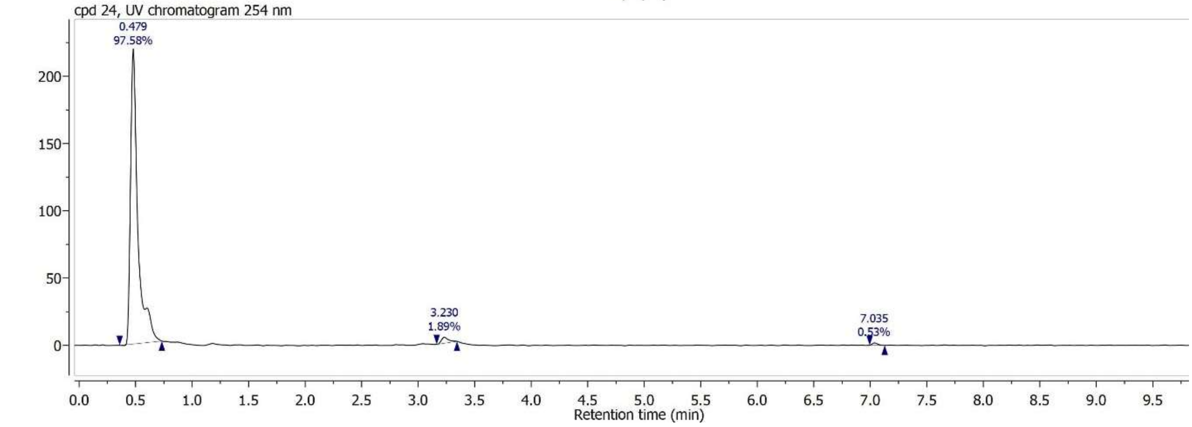

LC-ESI-MS (measured with Poroshell column, gradient 10–90% ACN in water + 0.1% formic acid over 10 min), top: mass spectrum, bottom: LC chromatogram at 254 nm. Calculated: m/z = 220.2 [M + H^+], found: m/z = 220.0 [M + H^+], purity 97.6%.

24

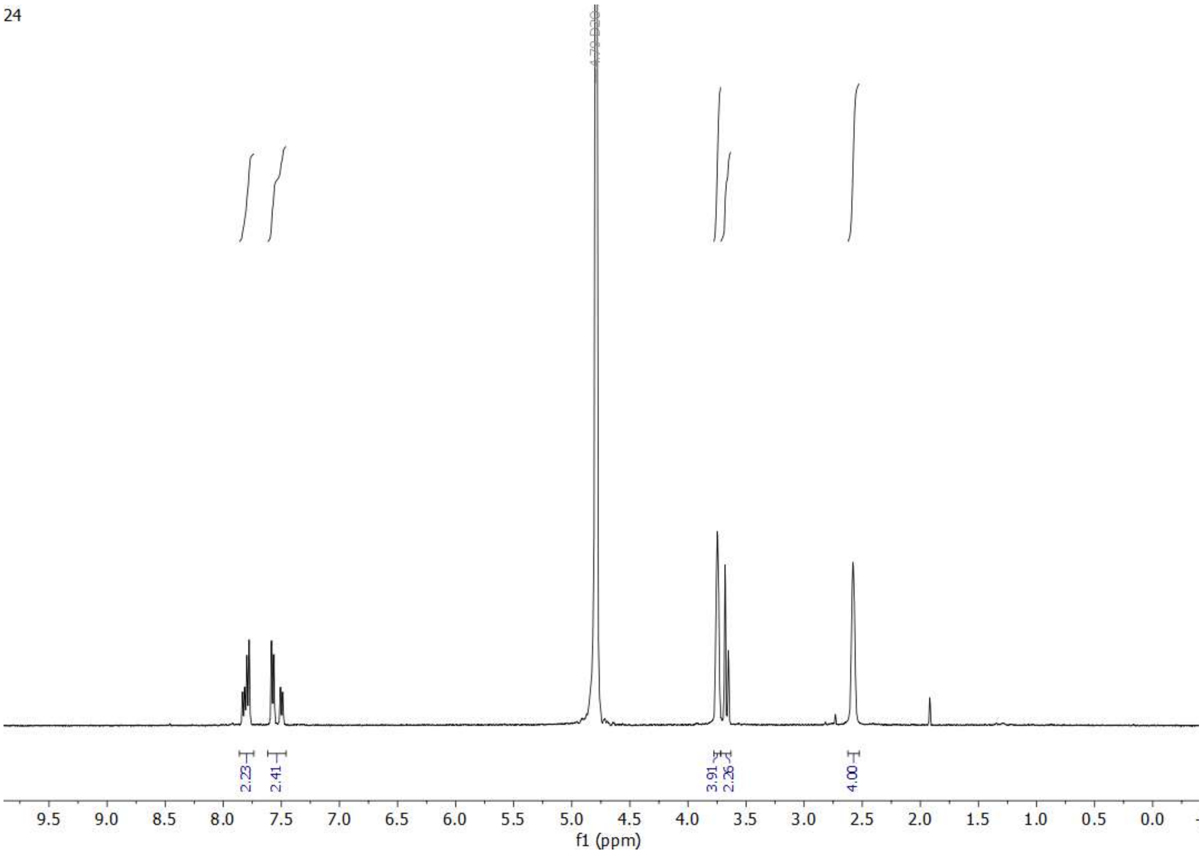

^{1}H NMR (400 MHz, Deuterium Oxide, TMS) δ_H 7.86 – 7.75 (2H, m), 7.60 – 7.46 (2H, m), 3.79 – 3.72 (4H, m), 3.70 – 3.63 (2H, m), 2.65 – 2.51 (4H, m).

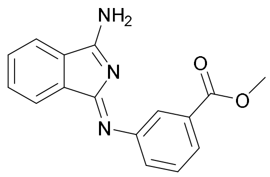

25

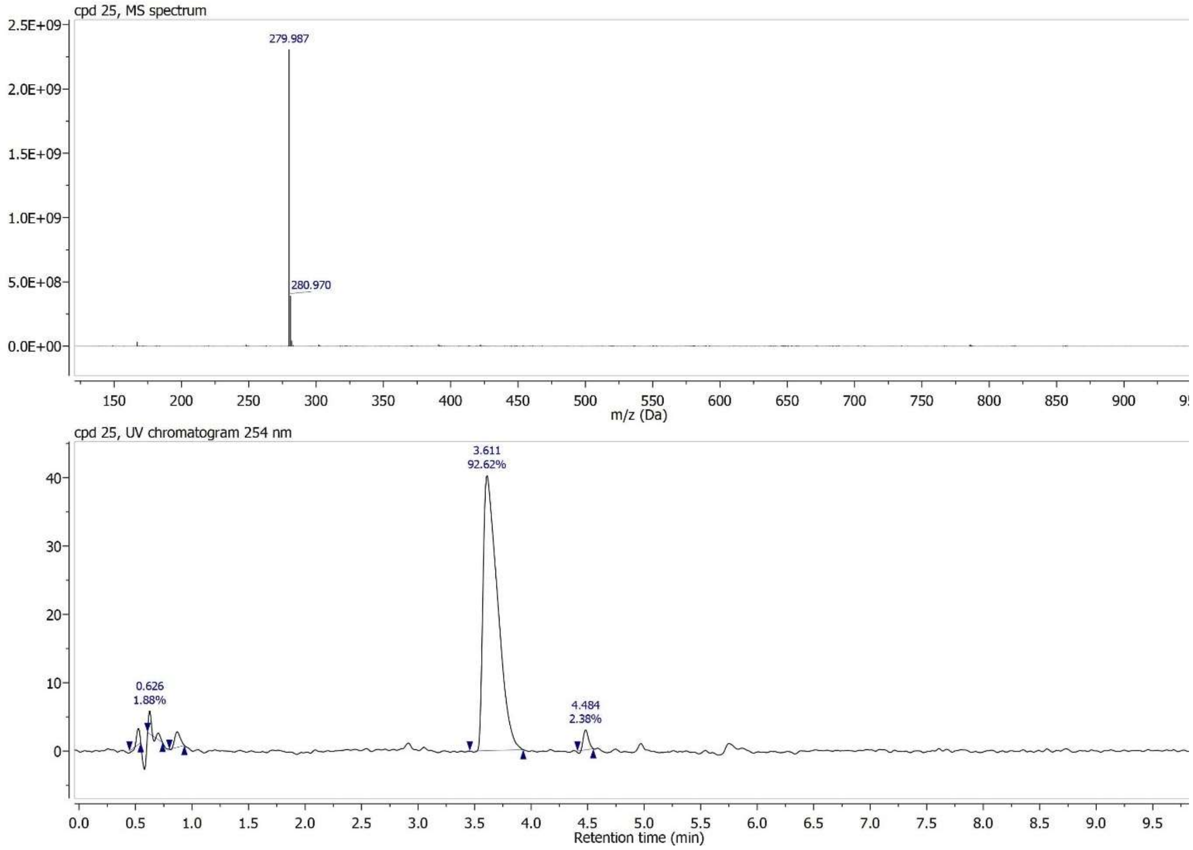

LC-ESI-MS (measured with Zorbax column, gradient 10–90% ACN in water + 0.1% formic acid over 10 min), top: mass spectrum, bottom: LC chromatogram at 254 nm. Calculated: m/z = 280.1 [M + H^+], found: m/z = 280.0 [M + H^+], purity 92.6%.

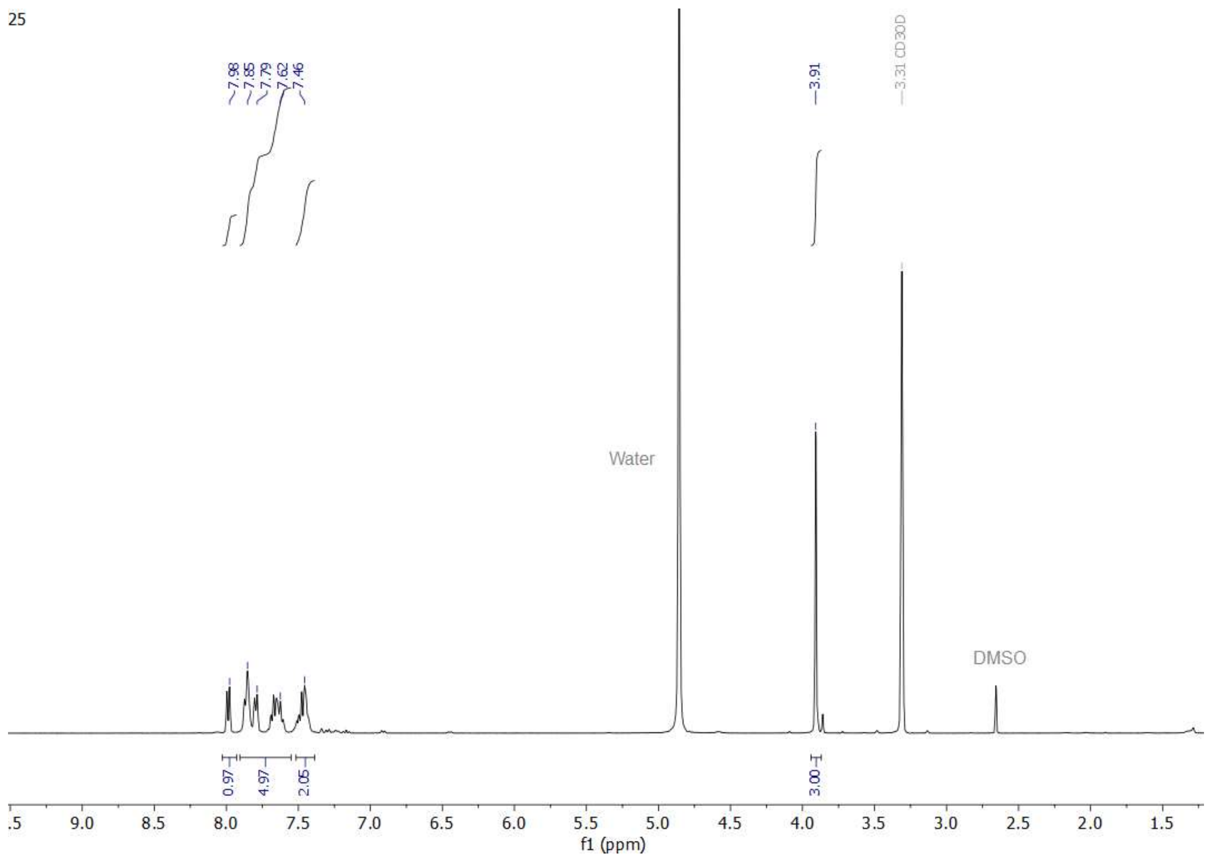

^{1}H NMR (400 MHz, Methanol-*d*$_4$, TMS) δ_H 7.99 (1H, d, *J* = 7.2 Hz), 7.91 – 7.55 (5H, m), 7.56 – 7.37 (2H, m), 3.91 (3H, s).

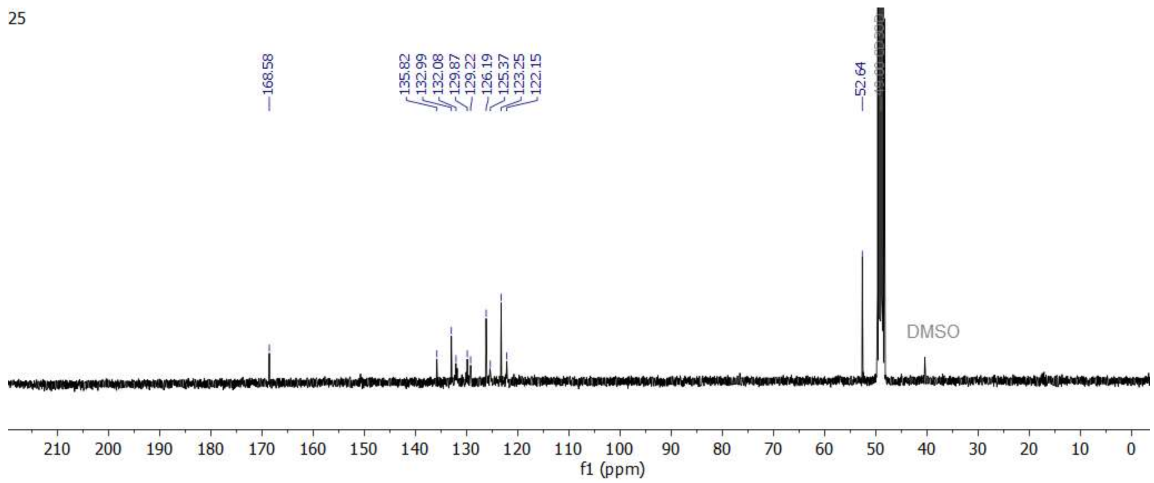

^{13}C NMR (101 MHz, Methanol-*d*$_4$, TMS) δ_C 168.58, 135.82, 132.99, 132.08, 129.87, 129.22, 126.19, 125.37, 123.25, 122.15, 52.64.

5. References

1 D. Schmit, U. Milewicz, M. A. Boerneke, S. Burley, K. Walsworth, J. Um, D. Hecht, T. Hermann and B. M. Bergdahl, *Aust J Chem*, 2020, **73**, 212–221.

2 M. A. Parker, E. Satkiewicz, T. Hermann and B. M. Bergdahl, *Molecules*, 2011, **16**, 281–290.
